# The effect of comorbidities on diagnostic interval for lung cancer in England: a cohort study using electronic health record data

**DOI:** 10.1038/s41416-024-02824-2

**Published:** 2024-08-23

**Authors:** Imogen Rogers, Max Cooper, Anjum Memon, Lindsay Forbes, Harm van Marwijk, Elizabeth Ford

**Affiliations:** 1 Department of Primary Care and Public Health, Brighton and Sussex Medical School, Falmer, UK; 2 Centre for Health Service Studies, University of Kent, Canterbury, UK

**Keywords:** Lung cancer, Diagnosis, Comorbidities

## Abstract

**Background:**

Comorbid conditions may delay lung cancer diagnosis by placing demand on general practioners’ time reducing the possibility of prompt cancer investigation (“competing demand conditions”), or by offering a plausible non-cancer explanation for signs/symptoms (“alternative explanation conditions”).

**Method:**

Patients in England born before 1955 and diagnosed with incident lung cancer between 1990 and 2019 were identified in the Clinical Practice Research Datalink and linked hospital admission and cancer registry data. Diagnostic interval was defined as time from first presentation in primary care with a relevant sign/symptom to the diagnosis date. 14 comorbidities were classified as ten “competing demand“ and four “alternative explanation” conditions. Associations with diagnostic interval were investigated using multivariable linear regression models.

**Results:**

Complete data were available for 11870 lung cancer patients. In adjusted analyses diagnostic interval was longer for patients with “alternative explanation” conditions, by 31 and 74 days in patients with one and ≥2 conditions respectively versus those with none. Number of “competing demand” conditions did not remain in the final adjusted regression model for diagnostic interval.

**Conclusions:**

Conditions offering alternative explanations for lung cancer symptoms are associated with increased diagnostic intervals. Clinical guidelines should incorporate the impact of alternative and competing causes upon delayed diagnosis.

## Introduction

Lung cancer is the leading cause of cancer mortality in the UK [1]. Lung cancer survival rates are relatively poor in the UK compared to other high income countries [2], with an age-standardised 5 year survival estimate of 13% in 2010–14, compared to 17% in France and 18% in Germany [3]. This is partly due to later stage at diagnosis [4]. More than 70% of patients in the UK are diagnosed at stage 3 or 4 [1], and in a comparative study of non-small-cell lung cancer patients aged 66 y and over only 15% of patients in England were diagnosed at Stage 1, compared to 25% in the United States [5] Delay between a patient first noticing a symptom and diagnosis may occur before presentation to primary care, in primary care or in secondary care [6]. Shortening the overall diagnostic interval (DI) i.e., the time between first presentation in primary care with a potential cancer symptom and cancer diagnosis, might limit the proportion of patients diagnosed at a later stage [7, 8] and improve survival [9].

Primary care consultation rates generally increase before cancer diagnosis [10]—this may represent a “missed opportunity” to decrease DI. The number of pre-referral consultations varies by tumour site, and is relatively high for lung cancer, with around 30% of lung cancer patients having multiple consultations as compared to <10% of melanoma or breast cancer patients [11]. Current NHS guidelines for suspected lung cancer recommend cancer pathway referral for a specialist secondary care appointment within two weeks for patients with unexplained haemoptysis or chest x-ray results suggestive of cancer, or chest x-ray within 2 weeks for patients aged over 40 y with either one or two other potential cancer symptoms, depending on the patient’s smoking history [12].

There is evidence that the DI for cancer may be increased by the presence of comorbidities [13, 14]. A recent review found that the presence of pre-existing diseases is associated with increased DIs across a range of cancers including leukaemia, myeloma, oesophageal, colorectal and laryngeal cancers [13]. It has been suggested that comorbidities can be grouped into two categories affecting DI via distinct mechanisms [14]: (1) “competing demands” i.e., conditions that are unrelated to the cancer but place a burden on the general practitioner’s (GP) time and (2) “alternative explanations” i.e., conditions that provide plausible alternative explanations for cancer signs/symptoms in the diagnostic process and delay referral for cancer investigation in secondary care as a result. For lung cancer potential “alternative explanation” comorbidities include common chronic conditions such as asthma and chronic obstructive pulmonary disease (COPD). One qualitative analysis of significant event audits of lung cancer diagnoses from 92 general practices in England found that the presence of asthma or COPD was recognised by GPs to have the potential to mask lung cancer symptoms and result in diagnostic delay [15]. Similarly, in a case series of lung cancer patients in Denmark it was noted that attribution by the GP of lung cancer symptoms to existing disease was associated with prolonged diagnostic delay for some patients [16]. In an analysis of the Surveillance, Epidemiology and End Results programme (SEER) data on lung cancer patients in the US, Nadpara et al found a marked association between presence of comorbidities as assessed by Charlson score and DI [17], and COPD and asthma were both found to be associated with delayed lung cancer referral in a study in Morocco [18]. Pearson et al. found a positive association between comorbidities and secondary care diagnostic interval among English lung cancer patients [19]. An experimental study of different case vignettes of possible lung and colorectal cancer presented to physicians in the UK found that the presence of an alternative explanation decreased the likelihood of a prompt referral for cancer investigation [20]. However, there is limited published evidence on the association between the presence of comorbidities and DI in representative samples of lung cancer patients in the UK.

In the paper, we investigated the associations between comorbidities and DI in a large cohort of patients with lung cancer in the UK. We hypothesised that the presence of one or more comorbidities would increase the DI, and that this effect would be more marked for comorbidities, such as chronic respiratory conditions, which might offer an alternative explanation for lung cancer symptoms. We also aimed to assess whether any associations between DI and comorbidities were moderated by the presenting cancer sign/symptom or the patient’s smoking status, and to investigate the effect of a number of other measures of patient characteristics i.e., body mass index (BMI) and alcohol drinking, and area-based measures of social deprivation.

## Methods

This study used routinely collected data from general practice (i.e., primary care) consultations in England from the UK Clinical Practice Research Datalink (CPRD) [21] dataset, and linked data [22] at the individual patient level with the cancer registrations, secondary care admissions and Office for National Statistics data on deaths and social deprivation.

### Clinical Practice Research Datalink (CPRD)

The CPRD contains pseudonymised healthcare records from over 20 million people. The data are derived from the longitudinal electronic health records (EHRs) kept at the primary care practice. CPRD is divided into two databases, Gold and Aurum according to the clinical record-keeping software used at the practice—CPRD Gold data was used in this study. Information on diagnoses, symptoms, consultations, referrals, prescriptions, test results and other patient characteristics such as BMI, smoking and alcohol drinking habits is available. Most clinical data within CPRD Gold are recorded using Read codes [23], a structured hierarchical coding system with each code representing a health-related concept (eg. diagnoses or symptoms).

### Hospital episodes statistics (HES)

The linked HES data [24] used included medical records from admission and day case procedures but not data from outpatient appointments (where diagnostic information is recorded in <5% of attendances [25]) or emergency department visits without admission. Diagnoses are recorded using ICD-10 codes.

### Cancer registry

The cancer registry data is held by the National Cancer Registration and Analysis Service [26]. New cases of cancer are recorded based on information from sources including health care providers and death certificates. Cancer diagnoses are recorded using ICD-10 codes.

### Office for National Statistics Death data

Dates of death of the lung cancer patients were obtained from the ONS [27] and were used to identify patients where date of diagnosis was on or after date of death.

### Index of multiple deprivation

Data on index of multiple deprivation (IMD) was also available based on the GP practice postcode [28].

### Clinical code lists

Clinical code lists used to define cases of lung cancer (including cancers of the trachea and bronchus), symptoms, comorbidities, ethnic group, and other patient characteristics are available in the supplementary information. Code lists were drawn up by review of published code lists [29] and by searching the medical and product dictionaries supplied by CPRD.

### Study population and representativeness

Data were available on 800,018 patients, i.e., all patients in CPRD Gold born in 1954 or earlier who were still registered with a GP on their 65th birthday and who were eligible for linkage with the cancer registry, hospital episodes statistics, Office for National Statistics and IMD data (eligibility for linkage was determined by consent to the linkage process by the GP practice and the availability of a valid NHS number [30]). From this sample primary lung cancer cases were identified from 3 data sources, the CPRD primary care data, and the linked hospital episodes statistics and cancer registry data, using lists of Read codes or ICD-10 codes as appropriate. The CPRD data has been shown to be broadly representative of the UK population in terms of age, sex, and ethnicity when compared to the UK census and to the Health Survey for England in terms of BMI [31]. However, given the changing ethnic make-up of the UK over time, and the fact our sample was drawn from those patients born in 1954 or earlier, the proportion of white patients was relatively higher than if a younger age range of patients had been included. In addition, among women the proportion of lung cancer cases diagnosed at age 70 y or over increased substantially over the study period [32], so the study cohort will be less representative of the total population of female lung cancer patients in the earlier time periods.

### Variable definitions

Diagnostic interval: DI was defined as the time between date of first presentation in primary care with a clinical problem suggestive of possible lung cancer, to the date of diagnosis (signs/symptoms and other clinical indicators up to 12 months before diagnosis were considered in line with a number of other studies of cancer DI conducted using the CPRD data [14, 33, 34]).

Presenting clinical problem: Signs/symptoms and other clinical problems considered to indicate possible lung cancer were cough, haemoptysis, loss of appetite, fatigue, dyspnoea, weight loss, thrombocytosis, chest infection, finger clubbing and lymphadenopathy—these were based on the National Institute for Health and Care Excellence (NICE) guidelines for suspected cancer recognition and referral [12] and on discussion with a GP (author MC). Due to small numbers of patients first presenting with finger clubbing, lymphadenopathy and appetite loss these were grouped together as “Other”, patients presenting with more than one clinical indicator initially were categorised as “Multiple”.

Date of diagnosis: this was taken to be the first date of an assigned diagnostic code for lung cancer present across the 3 data sources i.e., the CPRD primary care data, the HES data and the Cancer Registry data. Cancer Registry data were only available until the end of 2016, so for patients diagnosed between 2017 and 2019 date of diagnosis was taken as the first date of a diagnostic code for lung cancer in either the HES or the CPRD.

Multimorbidity: Following the classification from Mounce et al. [14] we considered the number of two classes of comorbidity diagnoses for each patient 1) “competing demand” (CD) conditions unrelated to lung cancer that place demand on the physician’s time and might reduce the focus on possible lung cancer symptoms 2) “alternative explanation” (AE) conditions i.e., conditions to which some lung cancer symptoms could reasonably be attributed. For the CD conditions we included coronary heart disease (CHD), depression/anxiety, heart failure, hypertension, chronic kidney disease (CKD), osteoporosis, dementia, serious mental illness (SMI), epilepsy and diabetes. For the AE conditions we included COPD, asthma, chronic fatigue syndrome (CFS), and receipt of a prescription for an Angiotensin-converting enzyme (ACE) inhibitor – the latter was included as cough is a common side effect of ACE inhibitors. As these were mostly chronic, non-resolving conditions, the presence of a diagnostic code at any point in the medical record, excluding the 12 months before diagnosis, was taken to indicate that the patient had the condition, an absence of a code was taken to indicate the absence of the condition (the 12 months prior to cancer diagnosis were excluded as we were interested in the effect of pre-existing comorbidities on DI). As depression/anxiety may resolve, only diagnoses in the 3 years prior to cancer diagnosis were taken to indicate the presence of the condition. For ACE inhibitor prescription, the relevant timeframe was taken to be any point in the 2 years prior to diagnosis.

Other variables: other factors considered included sex, age at diagnosis and year of diagnosis (categorised as shown in Table 1), usual consultation frequency measured as total number of consultations involving contact with a GP from 24 to 12 months before diagnosis, increase in consultation frequency in the year before diagnosis (yes/no) (12 month periods were chosen for comparison of consultation frequency as there is a substantial seasonal variation [35]), presenting clinical problem, IMD quintiles based on the GP practice address, and additional patient characteristics including smoking status (ever smoker versus non-smoker), alcohol drinking status (former, current or non-drinker), and BMI category (underweight or BMI < 18.5 kgm^−2^, normal weight or BMI of 18.5 to <25 kgm^−2^, overweight or BMI of 25 to <30 kgm^−2^, and obese or BMI ≥ 30 kgm^−2^). For BMI, smoking and alcohol drinking the most recent available measure was used, excluding measures in the 6 months before diagnosis as it was felt these might have been affected by the course of the disease. The patient’s ethnic group was also considered—there was substantial missing data in this variable and very small numbers in any ethnic group except for white (<1% in total) so the variable was grouped as white and other.

**Table 1:** Characteristics of the study population and univariate associations with diagnostic interval.

	*n* (%)	Median (IQR) diagnostic interval (days)	*p* value^a^
Sex			
Male	6884 (58.0)	133 (43, 263)	<0.001
Female	4986 (42.0)	152 (51, 278)	
Age (y)	76.2 (7.5)^b^	0.705 (0.144)^c^	<0.001
Year of diagnosis			
<2000	835 (7.0)	121 (42, 261)	<0.001
2000–2004	2586 (21.8)	117 (38, 251)	
2005–2009	3663 (30.9)	134 (44, 266)	
2010–2014	3325 (28.0)	168 (55, 287)	
2015–2019	1461 (12.3)	161 (56, 280)	
Number of CD conditions			
None	3958 (33.3)	101 (36, 241)	<0.001
One	4017 (33.8)	146 (49, 272)	
Two	2500 (21.1)	162 (55, 279)	
Three or more	1395 (11.8)	196 (80, 298)	
Number of AE conditions			
None	5785 (48.7)	90 (34, 224)	<0.001
One	4242 (35.7)	164 (56, 281)	
Two or more	1843 (15.5)	246 (137, 320)	
Number of consultations			
Usual frequency (per y)	10 (5, 17)^d^	2.41 (0.09)^c^	<0.001
Increase before diagnosis			
Yes 1	9039 (76.1)	139 (47, 269)	0.853
No 0	2831 (23.9)	143 (43, 273)	
IMD			
1	1321 (11.1)	1.44 (0.79)^c^	0.070
2	1795 (15.1)		
3	2156 (18.2)		
4	2824 (23.8)		
5	3774 (31.8)		
Presenting clinical problem			
Cough	3112 (26.2)	146 (51, 276)	<0.001
Dyspnoea	2582 (21.8)	172 (58, 285)	
Chest infection	2271 (19.1)	173 (68, 285)	
Chest pain	1014 (8.5)	113 (44, 245)	
Fatigue	728 (6.1)	146 (49, 266)	
Thrombocytosis	622 (5.2)	92 (35, 249)	
Multiple	545 (4.6)	133 (35, 268)	
Haemoptysis	450 (3.8)	37 (21, 83)	
Weight loss	399 (3.4)	56 (24, 184)	
Other	147 (1.2)	48 (26, 158)	
Ethnic Group			
White	10625 (89.5)	146 (49, 273)	0.100
Other	101 (0.9)	186 (70, 278)	
Missing	1144 (9.6)		
Smoking status			
Current or former smoker	10047 (84.6)	150 (49, 276)	<0.001
Non-smoker	1366 (11.5)	117 (43, 237)	
Missing	457 (3.9)		
BMI category			
Underweight	489 (4.1)	198 (72, 298)	<0.001
Normal weight	4351 (36.7)	147 (47, 274)	
Overweight	3687 (31.1)	135 (47, 268)	
Obese	1898 (16.0)	156 (55, 280)	
Missing	1445 (12.2)		
Alcohol drinking			
Current drinker	7939 (66.9)	140 (47, 268)	<0.001
Former drinker	1319 (11.1)	182 (64, 294)	
Non drinker	1186 (10.0)	156 (53, 281)	
Missing	1426 (12.0)		

^a^*p* value by Mann–Whitney *U* test or Kruskal–Wallis as appropriate b - mean (sd).

^b^mean (sd).

^c^B(SE).

^d^median (IQR).

### Exclusions

The following groups of patients were excluded from analyses: (1) patients with a current registration date <2 y before lung cancer diagnosis (2) patients who did not present with a relevant complaint in the year prior to diagnosis meaning DI could not be calculated (3) patients with a clinical code for chemo- or radio-therapy in the CPRD data prior to the diagnosis date (in these cases it was unclear when the cancer diagnosis was made) (4) patients with a diagnosis date after their death date (5) patients from practices with an up-to-standard date (the date from which data from a practice is considered to be of adequate quality for research) <1 y before their diagnosis date.

### Statistical analyses

Our outcome variable was DI in days—we aimed to investigate how this was associated with number of CD and AE conditions allowing for presenting clinical problem, patient characteristics and other variables. The DI distribution was skewed, therefore results of univariate analyses report the median DI in each category. Age, usual consultation frequency and IMD were treated as continuous variables, other predictor variables were categorised as shown in Table 1. Initially, univariate associations between potential predictors and DI were investigated using Mann–Whitney *U* Test and Kruskal–Wallis test as appropriate for categorical variables and univariate linear regression for continuous variables. Multiple linear regression models were chosen for the multivariable analyses for ease of interpretability, as the data were skewed a number of diagnostic tests and additional analyses were run to assess the effect of non-normality on the results obtained. A series of regression models were produced using the following procedure:

Model 1: All variables with minimal (<5%) missing data (sex, age and calendar year at diagnosis, number of CD and AE comorbidities, IMD, usual consultation frequency, increase in consultation frequency, and presenting symptom) were entered into an initial model, and a final model selected using both backwards and bidirectional stepwise selection based on Akaike Information Criterion (AIC) [36].

Model 2: The effect of adding variables with significant amounts of missing data (ethnicity, BMI category, smoking status, alcohol drinking) to the final selected Model 1 was then investigated.

Model 3: The effect of adding interaction terms to the model between AE comorbidities and smoking, between presenting symptom and smoking and between AE comorbidities and presenting symptom was also investigated—all three interactions were added to Model 2 initially and backwards stepwise selection was used to select those that were significant at *p* < 0.05.

Model 4: As a final step we repeated the backwards selection procedure used in Model 1 replacing the grouped variables for CD and AE conditions with all the individual comorbidities as separate dummy variables in the initial model. This was in order to identify any individual comorbidities with a particularly marked effect on DI.

## Results

**Fig. 1:**
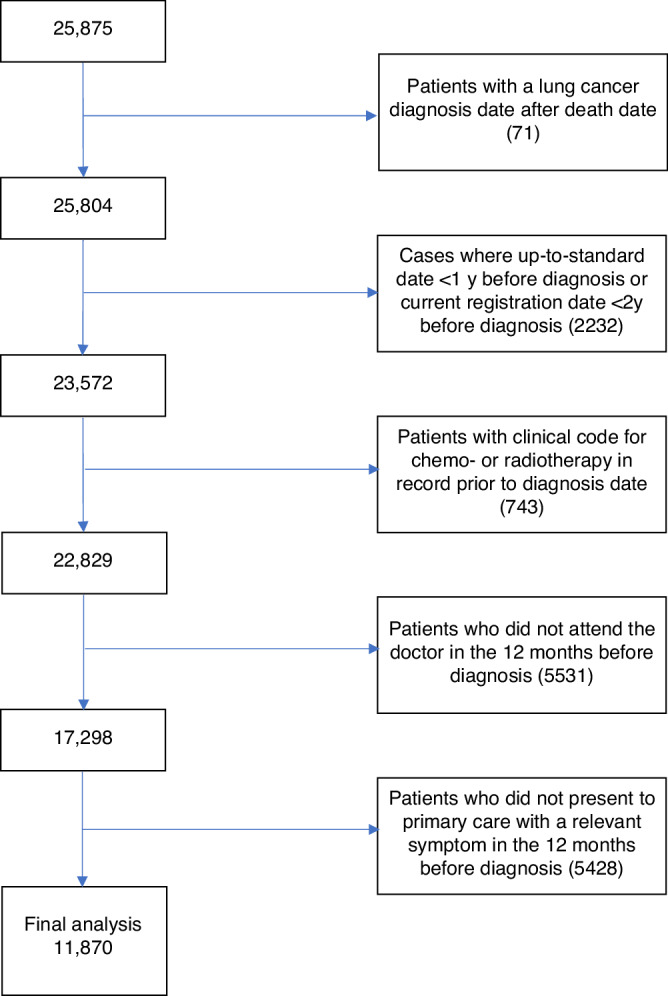
Flow chart of sample selection. Flow chart showing number of included patients and exclusions.

Figure 1 shows a flow chart of the process of patient selection. 25875 lung cancer patients were identified in total across the 3 data sources (the CPRD primary care data and the linked HES and cancer registration data). Following exclusions, 11870 of these presented with a relevant sign/symptom allowing calculation of DI, these patients formed our final sample for analysis. Descriptive data on these patients are given in Table 1. Median (IQR) DI across all included patients was 140 (46, 270) days.

### Univariate analyses

In univariate analyses, all the potential predictor variables were significantly associated with DI with the exception of ethnic group and increase in consultation rate before diagnosis (Table 1). Presenting sign/symptom was associated with large differences in DI which varied from a median of 37 days in patients presenting with haemoptysis to 173 days in those presenting with chest infection. DI was also substantially higher in patients with more CD conditions (median of 196 days in those with three or more conditions versus 101 days in those with none) and those with more AE conditions (median of 246 days in those with two or more conditions versus 90 days in those with none). DI was also somewhat higher in females, in ever- versus never smokers, in underweight patients, and among former drinkers.

### Median DI stratified by number of AE and CD conditions

**Fig. 2:**
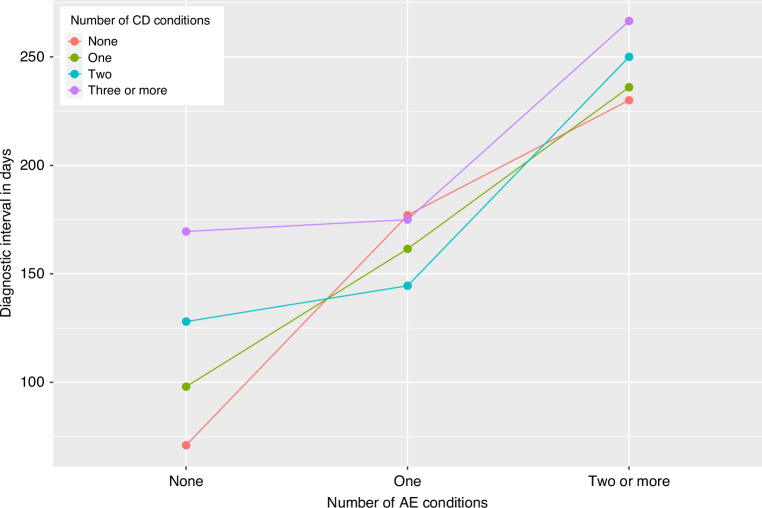
Median diagnostic interval by number of AE and CD conditions. Median diagnostic interval stratified by grouped number of Alternative Explanation and Competing Demand conditions.

Figure 2 shows the median DI across number of AE conditions stratified by number of CD conditions. Median DI increased by number of AE conditions irrespective of number of CD conditions. For patients with no AE conditions median DI increased with number of CD conditions, however, for those with one or more AE conditions, DI was similar across strata of CD conditions.

### Multivariable analyses

Results of Models 1 and 2 are shown in Table 2. Regression coefficients shown in the table represent the mean change in DI in days for a unit change in the predictor, or for categorical variables the difference in days from the reference category.

**Table 2:** Results of linear regression model for diagnostic interval in days.

	Model 1 (*n* = 11870)		Model 2 (*n* = 9837)	
Predictor	Adjusted B (95% CI) (days)	*p*	Adjusted B (95% CI) (days)	*p*
Sex (female)	7.24 (3.25, 11.24)	<0.001	4.95 (0.45, 9.45)	0.031
Age at diagnosis (y)	0.355 (0.090, 0.620)	0.009	0.313 (0.015, 0.611)	0.040
Diagnosis year				
<2000	0.00	—	0.00	—
2000–2004	−12.82 (−21.35, −4.28)	0.003	−12.18 (−22.77, −1.59)	0.024
2005–2009	−10.70 (−19.00, −2.41)	0.011	−11.40 (−21.68, −1.13)	0.030
2010–2014	−7.91 (−16.40, 0.58)	0.068	−9.78 (−20.21, 0.65)	0.066
2015–2019	−17.30 (−26.86, −7.75)	<0.001	−19.36 (−30.75, −7.98)	<0.001
AE conditions				
None (reference)	0.00	—	0.00	—
One	30.55 (26.09, 35.00)	<0.001	27.94 (23.03, 32,85)	<0.001
Two or more	73.83 (67.80, 79.87)	<0.001	70.97 (64.45, 77.48)	<0.001
Background consultation frequency (visits/y)	2.03 (1.85, 2.22)	<0.001	1.98 (1.78, 2.18)	<0.001
Increase in frequency				
No increase (reference)	0.00	—	0.00	—
Increase	22.72 (17.78, 27.65)	<0.001	22.52 (17.11, 27.93)	<0.001
IMD	1.74 (0.30, 3.19)	0.018	1.60 (0.00, 3.20)	0.050
First symptom				
Cough (reference)	0.00	—	0.00	—
Dyspnoea	−7.67 (−13.46, −1.88)	0.009	−4.88 (−11.21, 1.46)	0.131
Chest infection	9.11 (3.18, 15.04)	0.003	11.05 (4.43, 17.67)	0.001
Chest pain	−17.79 (−25.51, −10.06)	<0.001	−19.55 (−28.00, −11.11)	<0.001
Fatigue	−9.10 (−17.92, −0.28)	0.043	−6.50 (−16.22, 3.23)	0.191
Thrombocytosis	−28.65 (−38.05, −19.25)	<0.001	−26.85 (−37.34, −16.35)	<0.001
Multiple	−17.79 (−27.73, −7.85)	<0.001	−17.30 (−28.06, −6.54)	0.002
Haemoptysis	−82.61 (−93.41, −71.81)	<0.001	−84.23 (−96.48, −71.98)	<0.001
Weight loss	−50.52 (−61.87, −39.17)	<0.001	−52.04 (−64.72, −39.37)	<0.001
Other	−60.97 (−78.99, −42.95)	<0.001	−66.03 (−86.21, −45.85)	<0.001
BMI Category				
Healthy weight (reference)			0.00	—
Underweight			23.35 (12.74, 33.95)	<0.001
Overweight			−5.89 (−10.85, −0.93)	0.020
Obese			−3.60 (−9.75, 2.55)	0.251
Smoking status				
Ever smoker (reference)			0.00	—
non-smoker			−11.43 (−18.63, −4.23)	0.002
Missing				
Drinking status				
Current drinker (reference)			0.00	—
Former drinker			6.68 (0.08, 13.28)	0.047
Non drinker			8.24 (1.10, 15.38)	0.024

Model 1: The backwards stepwise regression procedure resulted in the number of CD conditions being dropped from the initial linear regression model, all other variables were retained. DI was slightly higher for females than males (B of 7.2 (95%CI 3.3, 11.2) days) and increased slightly with age and decreased with more recent calendar year of diagnosis. Presenting clinical problem was strongly associated with DI. Patients presenting with haemoptysis or weight loss were diagnosed substantially more quickly (regression coefficient (B) of −82.6 (95%CI −93.4, −71.8) and −50.5 (−61.9, −39.2) days respectively) compared to patients presenting with cough. DI was greater in those with a higher usual consultation rate (by around 2 days per consultation), and an increase in consultation rate in the year before diagnosis was also associated with a longer DI in the multivariable analysis. DI was strongly associated with the presence of AE conditions, being 30.6 (26.1, 35.0) and 73.8 (67.8, 79.9) days higher among those with one and two or more AE conditions respectively compared to patients with none.

Model 2: When ethnicity was added to Model 1 there was no evidence of an association (*p* = 0.143). Given the relatively large amount of missing data and the very small numbers in any ethnic group other than white this variable was not included in subsequent analyses. The results of adding smoking status, BMI category and alcohol drinking to the model are shown in Table 2. There was evidence that diagnosis was delayed in underweight patients compared to patients in the normal BMI category (B of 23.4 (12.7, 34.0) days)—this was in contrast to the association of weight loss as a symptom with reduced DI. Diagnosis was slightly faster in non-smokers compared to ever−smokers (B −11.4 (−18.6, −4.2) days). There was also some evidence that DIs were longer for former drinkers (B 6.7(0.1, 13.3) days) and non-drinkers (B 8.2 (1.1, 15.4) days) compared to current drinkers. Adding these variables to the model slightly reduced the association of DI with sex, estimates for other predictors remained similar.

Model 3: Only the interaction between number of AE conditions and first symptom was retained in the backwards selection procedure (*p* < 0.001). Full results are shown in Table S1 in the supplementary information. Notably, there was an indication that DIs for patients with dyspnoea were longer in those with an AE condition (by 25 days).

Model 4: The comorbidities remaining in the final model are shown in Table 3. Three AE comorbidities remained in the model, COPD, asthma and ACE inhibitor prescription – these were associated with a 59.1, 30.6 and 7.7 day increase in DI respectively. CHD was also associated with a relatively large increase in DI (18.5 days), the other conditions remaining in the model were depression/anxiety, osteoporosis and epilepsy which were all associated with increased DI (by 12.5, 10.3 and 13.6 days, respectively), and diabetes which was associated with a 5.6 day decrease in DI.

**Table 3:** Association between individual comorbid conditions and diagnostic interval.

Comorbidity	Adjusted B^a^ (95% CI) in days (*n* = 11870)	*p*
CHD	18.46 (13.51, 23.41)	<0.001
Depression/anxiety	12.51 (4.88, 20.14)	0.001
Epilepsy	13.61 (−1.90, 29.12)	0.085
Diabetes	−5.56 (−11.53, 0.41)	0.068
Osteoporosis	10.28 (2.10, 18.46)	0.014
Asthma	30.61 (25.00, 36.22)	<0.001
COPD	59.07 (53.98, 64.16)	<0.001
ACE	7.73 (3.28, 12.17)	<0.001

^a^Regression coefficients are adjusted for age at diagnosis, year at diagnosis, usual consultation frequency, increase in consultation frequency in year before diagnosis, presenting clinical problem and other conditions shown in table.

### Additional analyses

As the distribution of DI was skewed Models 1 and 4 were rerun using the log and square root of DI—this did not improve the fit and so the original models using the untransformed DIs were retained. There was evidence of significant heteroskedasticity (*p* < 0.001 by Breusch–Pagan test) and so Models 1 and 4 were rerun using a weighted linear regression - the effect estimates and standard errors obtained were essentially unchanged (Table S2 in the supplementary information). Bias-corrected accelerated bootstrap confidence intervals were also constructed for the coefficients in Model 1 as this method is robust to non-normality [37]—the results were almost identical to those from the regression model (Table S3 in the supplementary information). Model 1 was repeated excluding patients with death certificate only diagnoses and those where date of diagnosis and date of death were the same, and the results obtained were very similar (Table S4 in the supplementary information). Model 1 was also repeated with the increase in consultation frequency in the year before diagnosis entered as a continuous variable rather than dichotomised to yes/no (Table S5 in the supplementary information). The increase in DI associated with a unit increase in yearly frequency of consultations in the year before diagnosis was 1.9 (95%CI 1.7, 2.1) days, other coefficients in the model remained similar.

Model 2 was repeated using data with missing values for BMI, smoking status and alcohol drinking imputed by multivariate imputation by chained equations using the MICE package in R [38]. Adding imputed data for missing values had a minimal effect on the regression coefficients. (Table S6 in the supplementary information).

Finally, for the 5884 patients for whom information on date of chest x-ray was available in the primary care data, we repeated Model 1 using the interval from first presentation in primary care with a relevant sign/symptom to chest x-ray (x-ray interval). This interval was included as it was felt it might be an indication of the primary care (as opposed to secondary care) element of the DI. The observed patterns of association between the predictor variables and x-ray interval were similar to those for DI, with the presence of one or two or more AE conditions being associated with a 21.8 and 60.7 day increase in x-ray interval respectively (Table S7 in the supplementary material).

## Discussion

### Summary

Patients with one or more comorbid conditions that could provide an alternative explanation for their lung cancer symptoms had substantially increased intervals from presentation in primary care to diagnosis, by 31 and 74 days for patients with one and two or more conditions respectively. The number of CD conditions did not remain in the final model selected by the stepwise procedure. More frequent GP consultations were also associated with a longer time from presentation in primary care to diagnosis. As expected, the “red flag” symptom of haemoptysis was associated with a substantially lower DI. DI was longer in smokers and in underweight patients, suggesting an association between other markers of poor health and delayed diagnosis. For individual conditions the greatest increase in DI was associated with COPD (59 days).

### Interpretation of results and comparison with literature

While a number of studies have considered overall multimorbidity or the effect of respiratory disorders in relation to lung cancer DI, we believe this is the first study of lung cancer to group comorbidities as AE and CD conditions. In the model where the comorbidities were considered individually, we found significant increases in DI for three of our suggested AE conditions, ie, COPD, asthma and ACE inhibitor prescription. The diagnostic delay for cancer we have observed for patients with COPD and asthma is in line with previous studies [16–18]. To our knowledge, this is the first study to show an association between ACE inhibitor prescription and increased DI. The mean increase (around 8 days) was relatively small but was independent of the presence of several common chronic conditions associated with ACE prescription. In the model where comorbidities were considered individually, we also found strong associations between CHD and depression/anxiety and increased DI. Although we considered CHD as a CD condition, both dyspnoea and chest pain are common symptoms of CHD, so it is possible that it may be acting as an AE condition. However, both CHD and depression/anxiety have also been found elsewhere to be positively associated with DI for colorectal cancer [14, 39].

It has been suggested that the difference in the average number of pre-referral consultations for different cancers relates to their typical symptom patterns, with patients with more specific symptoms (e.g., a breast lump) being referred sooner than those with less specific symptoms (e.g., fatigue or dyspnoea) [11, 40, 41]. However, there is relatively little information on the associations between presenting cancer symptom and DI [40]. We found that DI was lowest for patients presenting with haemoptysis, a key “red flag” symptom for cancer, and was also relatively low for weight loss. Similarly, Walter et al. found that haemoptysis was the symptom associated with the shortest DI in 963 patients with suspected lung cancer [42]. A number of other studies of both lung [33, 34, 43] and other cancers [33] have found shorter DI among patients with “alert/alarm” versus “vague/non-alert” symptoms [34, 44]. It seems likely that some cancer symptoms would be more likely than others to be attributed to comorbidities, but few studies have considered the influence of symptoms and AE conditions simultaneously. We found evidence of an interaction between AE conditions and presenting clinical problem on DI, notably an additional increase of 25 days in patients with both dyspnoea and an AE condition. This suggests that symptoms of dyspnoea may be particularly likely to be attributed to an existing comorbidity among patients with an AE condition.

An increase in consultation rate is a risk marker for cancer [10]. Consultation rates rise for patients with cancer around four to six months before diagnosis, with this increase occurring independently of usual consultation frequency [45]. However, high habitual consultation rates have been found to reduce the GP’s suspicion of cancer, particularly in older patients [46]. Multimorbidity is strongly associated with GP consultation rates [47], and has been found to contribute to the rate of GP burnout [48]. In our study, both higher usual consultation frequency and an increase during the year before diagnosis were strongly positively associated with longer DI in the multivariable analyses. The positive association between the increase in consultation rates in the year before diagnosis may reflect an increase in diagnostic investigations undertaken in primary care. Frequently attending patients may have had a referral made for investigation of another serious illness regarded as a more likely explanation for their symptoms by the GP. However, the association with higher usual consultation rate in the pre-diagnostic period is unlikely to be explained in this way. It is also possible that the positive associations between consultation rates and DI may result from a focus on psychological or anxiety-related, rather than physical, explanations for symptoms [49] (which would accord with the delay in diagnosis associated with a depression/anxiety diagnosis in this study).

Smoking is the key risk factor for lung cancer, and the NICE guidelines on lung cancer referral differ depending on the patient’s smoking status [12]. Somewhat counterintuitively we found that DI was longer in smokers. It is possible this may reflect a tendency by GPs to view the effects of smoking (rather than possible cancer) as an “alternative explanation” for symptoms such as breathlessness.. This apparent increase in DI among patients with a history of smoking is concerning, as there is evidence that they may also wait longer before presenting to primary care with lung cancer symptoms because of a fear of blame or stigma [50].

We also found an association between BMI and delayed cancer diagnosis, with DI being around 23 days longer in underweight compared to normal weight patients, although weight loss as a symptom was associated with more rapid diagnosis. While weight loss as a symptom is frequently included in analyses, there seems to be little other information in the literature on the association between pre-diagnostic BMI and DI. The number of underweight patients was relatively small so these results should be treated with caution, but it may be that weight loss as a possible cancer symptom is less easily recognised in patients who are already underweight.

Although the unadjusted median DIs increased over the study period, the adjusted DI, taking account of presenting sign/symptom and other factors, decreased in recent years. Referral guidelines changed over the study period, with a 2 week wait guideline for cancer referral being introduced in 2000 [51]. There have also been a number of public information campaigns over this timeframe resulting in increased awareness of early cancer symptoms [52], including the “Be Clear on Cancer” campaign [53]. Evidence suggests the proportion of patients presenting with different lung cancer symptoms has changed over time, with more patients presenting with cough, and fewer with haemoptysis [54]. Additionally, while the common and red flag symptoms for lung cancer (haemoptysis, cough, dyspnoea etc.) have been well-established for decades, there has been a gradual acceptance of other indicators, such as thrombocytosis over the study period. Access to a GP may also have changed over the study period – the number of GPs per capita in England increased between 1997 and 2009 but then fell between 2009 and 2018 [55].

Complex patterns of association between DI and cancer mortality have been observed, including apparently paradoxical associations between shorter DI and increased mortality, which may be explained by more severe symptoms from aggressive tumours leading to quicker diagnosis (“waiting time paradox”) [34, 56, 57]. Despite the inconsistent nature of observed associations between DI and mortality, there is no plausible mechanism by which increased DI can reduce mortality, and increased tumour size is associated with higher mortality within TNM stage group [58]. Faster diagnosis allows quicker referral to other services including palliative care, and there are other adverse consequences to longer DI, including increased patient anxiety [59].

### Strengths and limitations

A key strength of this study is the use of the CPRD and linked secondary care and cancer registration data, which covers a large, broadly representative sample of the English population. As the data are routinely collected the study covers data from groups that may be hard to recruit to traditional research studies such as elderly/frail patients [60], patients living in deprived areas and those with literacy problems, although the preponderance of white patients meant we were not able to conduct a meaningful analysis of the effect of ethnic group. Cancer cases were identified from three sources (NCRAS, HES and CPRD), increasing the completeness of case identification and the accuracy of date of diagnosis determination [61], this is particularly important for studies investigating the effect of comorbidity as missing cancer diagnoses in the CPRD were more likely for older patients with comorbidities [61]. We were also able to identify those cases with a death certificate only diagnosis (where it could be argued that the calculated DI is artificially truncated) and to confirm that excluding these patients from the analysis did not materially affect the results.

However, there are a number of potential issues with the use of routinely collected data for research. Symptom identification in this study was dependent upon the accuracy of recording by the GP. Some clinical problems will have been noted in the free text area of the clinical records which we were unable to access. In addition, it has been suggested that some GPs may only be prompted to record a code for a symptom when the decision to refer has been made [34]. Both these factors could result in an underestimate of DI. It seems probable that this would occur more often for symptoms potentially explicable by existing conditions, so this bias would be likely to result in a reduction in the observed association between AE conditions and DI with the data available in our study, rather than inflating it.

A further issue is the relatively high levels of missing data for smoking status, alcohol drinking and BMI. These data are unlikely to be missing completely at random, with sicker patients and those consulting more frequently tending to have more complete data [62], and a complete case analysis may introduce bias [63]. The quality of data recording in the CPRD will have been affected by the introduction of the Quality and Outcomes Framework in 2004 [64], a scheme that offered financial incentives to GPs to encourage reporting of key data items. As a result, the completeness of recording of variables such as smoking status, BMI and alcohol drinking has increased substantially since that time [31]. We repeated our analysis with imputed data for missing values for smoking, BMI and alcohol drinking using an imputation process that included key recommended variables (measures of healthcare utilisation, health status/comorbidities and socioeconomic status [65]). Our results remained essentially unchanged, suggesting it is unlikely that the missing data would have substantially altered the observed associations.

We based our definition of DI on symptoms occurring up to 12 months before diagnosis. Taking into account the large number of patients in the study, and the lack of access to any text notes in the clinical record, our only option was to determine date of first symptom algorithmically from the date of the first relevant Read code in the EHR in the 12 months preceding diagnosis. However, some patients who go on to develop lung cancer may have had symptoms such as cough or dyspnoea for many years, and this method did not allow us to capture other potential indicators of true first cancer symptoms, such as a change in the nature or severity of cough.

The 12 month cut-off will result in the exclusion of a small number of patients with true DIs longer than a year, conversely, increasing the DI beyond this point would have resulted in more patients with symptoms unrelated to their subsequent cancer being included. We believe 12 months offers a reasonable compromise based on the low predictive value of symptoms recorded before this point for lung cancer [66], and is in line with previous work on cancer DI [14, 33, 34].

We have focussed our analyses on the factors associated with the overall DI from presentation in primary care to diagnosis, on the basis that this is the health system interval most likely to be associated with patient outcomes. However, delays can occur in both primary and secondary care, and may have different predictive factors. Using data on date of chest x-ray in the primary care record as a proxy for primary care interval, we found a similar pattern of associations to using the overall DI, although as we did not have access to the HES diagnostic imaging data there will have been substantial missing information in this variable (we had information on x-ray interval for 49% of the lung cancer patients, while a previous analysis of CPRD using the HES DID data suggests that 85% of patients had a pre-diagnostic x-ray [67]).

## Conclusions and Clinical Implications

This is one of only a few studies on how multimorbidity (i.e., AE and CD conditions) might impact on DI for lung cancer patients – substantial increases were found for patients with conditions offering alternative explanations for lung cancer symptoms. These findings highlight the difficulty of recognising cancer symptoms in the face of multimorbidity, and support the recent proposal to roll out a screening programme in the UK to detect lung cancer sooner [68]. Further studies to determine factors predictive of lung cancer development among patients with AE conditions might enhance this programme, by identifying those who would benefit most from regular screening. DI was also longer for patients with higher consultation frequency suggesting competing demand on the GP’s time may impact on timely diagnosis. Our results also suggest that diagnosis may be delayed in lung cancer patients who smoke or are underweight. Clinical guidelines should highlight the potential of these factors to impact on timely cancer diagnosis.

## Supplementary information

Supplementary data

Clinical Code Lists

## Data Availability

The data used in this study are provided under licence from the UK Medicines and Healthcare Products Regulatory Agency.

## Code availability

Data preparation code is available on zenodo (10.5281/zenodo.11549893).
