# Supplementary material for: The effect of comorbidities on diagnostic interval for lung cancer in England: a cohort study using electronic health record data
Source: Br J Cancer. 2024 Aug 23;131(7):1147–57. doi: 10.1038/s41416-024-02824-2 (PMC11442666; doi:10.1038/s41416-024-02824-2)
Supplement: Supplementary file 1 — Supplementary data [file 41416_2024_2824_MOESM1_ESM.docx]

**Supplementary data**

Table S1. Regression model for diagnostic interval in days with interaction terms between AE conditions and first symptom and smoking status and first symptom.

| Predictor | Adjusted B in days (95% CI) | p |
| --- | --- | --- |
| Sex (female) | 5.00 (0.50, 9.50) | 0.030 |
| Age at diagnosis (y) | 0.328 (0.030, 0.626) | 0.031 |
| Diagnosis year |  |  |
| <2000 (reference) | 0.00 | — |
| 2000 – 2004 | -11.76 (-22.35, -1.17) | 0.030 |
| 2005 – 2009 | -10.58 (-20.85, -0.30) | 0.044 |
| 2010 – 2014 | -9.96 (-20.39, 0.48) | 0.062 |
| 2015 – 2019 | -20.16 (-31.56, -8.77) | <0.001 |
| AE conditions |  |  |
| None (reference) | 0.00 | — |
| One | 26.02 (16.86, 35.18) | <0.001 |
| Two or more | 79.65 (66.34, 92.97) | <0.001 |
| Background consultation frequency (visits/y) | 1.97 (1.77, 2.18) | <0.001 |
| Increase in frequency |  |  |
| No Increase (reference) | 0.00 | — |
| Increase | 22.76 (17.35, 28.17) | <0.001 |
| IMD | 1.63 (0.03, 3.23) | 0.045 |
| First symptom |  |  |
| Cough (reference) | 0.00 | — |
| Dyspnoea | -19.97 (-30.26, -9.68) | <0.001 |
| Chest infection | 17.60 (8.15, 27.05) | <0.001 |
| Chest pain | -12.95 (24.51, -1.39) | 0.028 |
| Fatigue | -1.18 (-14.55, 12.18) | 0.862 |
| Thrombocytosis | -26.18 (-40.48, -11.87) | <0.001 |
| Multiple | -30.99 (-47.94, -14.03) | <0.001 |
| Haemoptysis | -73.35 (-89.69, -57.01) | <0.001 |
| Weight loss | -47.87 (-65.12, -30.61) | <0.001 |
| Other | -60.72 (-87.32, -34.11) | <0.001 |
| BMI Category |  |  |
| Healthy Weight (reference) | 0.00 | — |
| Underweight | 23.72 (13.12, 34.32) | <0.001 |
| Overweight | -5.57 (-10.53, -0.62) | 0.028 |
| Obese | -3.33 (-9.48, 2.82) | 0.288 |
| Smoking status |  |  |
| Ever Smoker (reference) | 0.00 | — |
| Non-smoker | -11.06 (-18.26, -3.87) | 0.003 |
| Drinking status |  |  |
| Current drinker | 0.00 | — |
| Former drinker | 6.99 (0.40, 13.59) | 0.038 |
| Non drinker | 8.39 (1.25, 15.53) | 0.021 |
| AE conditions: first symptom interaction |  |  |
| One condition:Dyspnoea | 25.45 (11.18, 39.72) | <0.001 |
| Two or more conditions:Dyspnoea | 11.18 (-6.72, 29.08) | 0.221 |
| One condition:Chest infection | -6.51 (-20.92, 7.89) | 0.375 |
| Two or more conditions:Chest infection | -27.81 (-47.36, -8.26) | 0.005 |
| One condition:Chest pain | -13.01 (-31.29, 5.28) | 0.163 |
| Two or more conditions:Chest pain | -17.88 (-45.26, 9.50) | 0.201 |
| One condition:Fatigue | -4.66 (-25.67, 16.36) | 0.664 |
| Two or more conditions:Fatigue | -31.75 (-62.75, -0.75) | 0.045 |
| One condition:Thrombocytosis | -0.59 (-23.16, 21.97) | 0.959 |
| Two or more conditions:Thrombocytosis | -2.63 (-37.58, 32.33) | 0.883 |
| One condition:Multiple | 18.98 (-5.37, 43.35) | 0.127 |
| Two or more conditions:Multiple | 23.74 (-5.15, 52.63) | 0.107 |
| One condition:Haemoptysis | -13.35 (-39.83, 13.12) | 0.323 |
| Two or more conditions:Haemoptysis | -64.26 (-106.09, -22.43) | 0.003 |
| One condition:Weight loss | -3.33 (-30.86, 24.19) | 0.812 |
| Two or more conditions:Weight loss | -26.01 (-66.50, 14.47) | 0.208 |
| One condition:Other | -7.77 (-52.01, 36.48) | 0.731 |
| Two or more conditions:Other | -27.51 (-95.73, 40.71) | 0.429 |

-

Table S2. Weighted linear regression models for diagnostic interval in days.

|  | Model 1  (n = 11870) |  | Model 4  (n = 11870) |  |
| --- | --- | --- | --- | --- |
| Predictor | Adjusted B (95% CI) (days) | p | Adjusted B (95% CI) (days) | p |
| Sex (female) | 7.15 (3.16, 11.15) | <0.001 | 5.61 (1.53, 9.68) | 0.007 |
| Age at diagnosis (y) | 0.354 (0.090, 0.618) | 0.009 | 0.390 (0.128, 0.653) | 0.004 |
| **Diagnosis year** |  |  |  |  |
| <2000 (reference) | 0.00 | — | 0.00 | — |
| 2000 – 2004 | -13.17 (-21.59, -4.74) | 0.002 | -12.06 (-20.43, -3.69) | 0.005 |
| 2005 – 2009 | -11.30 (-19.51, -3.09) | 0.007 | -9.17 (-17.34, -1.00) | 0.028 |
| 2010 – 2014 | -9.14 (-17.57, -0.71) | 0.034 | -6.42 (-14.82, 1.98) | 0.134 |
| 2015 – 2019 | -18.09 (-27.60, -8.59) | <0.001 | -16.30 (-25.77, -6.82) | <0.001 |
| **AE conditions** |  |  |  |  |
| None (reference) | 0.00 | — |  |  |
| One | 30.20 (25.77, 34.62) | <0.001 |  |  |
| Two or more | 73.40 (67.15, 79.65) | <0.001 |  |  |
| Background consultation frequency (visits/y) | 2.17 (1.97, 2.36) | <0.001 | 1.98 (1.78, 2.18) | <0.001 |
| **Increase in frequency** | 23.22 (18.29, 28.15) | <0.001 | 23.60 (18.72, 28.47) | <0.001 |
| No Increase (reference) |  |  |  |  |
| Increase |  |  |  |  |
| **IMD** | 1.68 (0.24, 3.11) | 0.022 |  |  |
| **First symptom** |  |  |  |  |
| Cough (reference) | 0.00 | — | 0.00 | — |
| Dyspnoea | -7.95 (-13.78, -2.12) | 0.008 | -14.13 (-19.96, -8.29) | <0.001 |
| Chest infection | 9.49 (3.50, 15.48) | 0.002 | 7.39 (1.49, 13.29) | 0.014 |
| Chest pain | -17.45 (-25.09, -9.81) | <0.001 | -17.98 (-25.57, -10.39) | <0.001 |
| Fatigue | -8.48 (-17.28, 0.32) | 0.059 | -7.55 (-16.25, 1.16) | 0.089 |
| Thrombocytosis | -28.93 (-38.15, -19.72) | <0.001 | -26.23 (-35.42, -17.04) | <0.001 |
| Multiple | -18.51 (-28.40, -8.62) | <0.001 | -22.54 (-32.34, -12.73) | <0.001 |
| Haemoptysis | -81.54 (-91.56, -71.53) | <0.001 | -82.71 (-92.94, -72.49) | <0.001 |
| Weight loss | -50.09 (-60.96, -39.23) | <0.001 | -49.83 (-60.77, -38.89) | <0.001 |
| Other | -60.36 (-77.28, -43.45) | <0.001 | -58.90 (-76.08, -41.72) | <0.001 |
| **Individual conditions** |  |  |  |  |
| CHD |  |  | 18.38 (13.39, 23.37) | <0.001 |
| Depression/anxiety |  |  | 12.48 (4.75, 20.21) | 0.002 |
| Osteoporosis |  |  | 10.20 (1.90, 18.50) | 0.016 |
| Epilepsy |  |  | 13.88 (-1.75, 29.51) | 0.082 |
| Diabetes |  |  | -5.79 (-11.79, 0.20) | 0.058 |
| Asthma |  |  | 30.84 (25.12, 36.56) | <0.001 |
| COPD |  |  | 59.17 (53.98, 64.35) | <0.001 |
| ACE prescription |  |  | 7.90 (3.44, 12.37) | <0.001 |

Table S3. Regression coefficients and confidence intervals obtained by bias-corrected accelerated bootstrap regression.

|  | Model 1 |
| --- | --- |
| Predictor | Adjusted B^a^ (95% CI) |
| Sex (female) | 7.24 (3.32, 11.26) |
| Age at diagnosis (y) | 0.355 (0.092, 0.623) |
| Diagnosis year |  |
| <2000 (reference) | 0.00 |
| 2000 – 2004 | -12.81 (-21.28, -4.18) |
| 2005 – 2009 | -10.70 (-19.10, -2.43) |
| 2010 – 2014 | -7.91 (-16.53, 0.77) |
| 2015 – 2019 | -17.30 (-26.85, -7.47) |
| AE conditions |  |
| None (reference) | 0.00 |
| One | 30.55 (26.10, 35.08) |
| Two or more | 73.83 (67.90, 79.80) |
| Background consultation frequency (visits/y) | 2.03 (1.82, 2.24) |
| Increase in frequency | 22.72 (17.59, 27.66) |
| IMD | 1.74 (0.30, 3.19) |
| First symptom |  |
| Cough (reference) | 0.00 |
| Dyspnoea | -7.67 (-13.54, -1.91) |
| Chest infection | 9.11 (3.17, 15.15) |
| Chest pain | -17.79 (-25.55, -10.00) |
| Fatigue | -9.10 (-18.09, 0.03) |
| Thrombocytosis | -28.65 (-37.74, -19.14) |
| Multiple | -17.79 (-27.84, -7.52) |
| Haemoptysis | -82.61 (-91.01, -73.63) |
| Weight loss | -50.52 (-61.35, -39.18) |
| Other | -60.97 (-76.49, -44.45) |

Table S4. Results of linear regression model excluding patients with death certificate only diagnoses.

|  | Model 1  (n = 11215) | p |
| --- | --- | --- |
| Predictor | Adjusted B (95% CI) (days) |  |
| Sex (female) | 7.44 (3.33, 11.55) | <0.001 |
| Age at diagnosis (y) | 0.318 (0.043, 0.593) | 0.023 |
| Diagnosis year |  |  |
| <2000 (reference) | 0.00 | — |
| 2000 – 2004 | -14.99 (-23.84, -6.14) | <0.001 |
| 2005 – 2009 | -13.96 (-22.53, -5.39) | 0.001 |
| 2010 – 2014 | -10.53 (-19.29, -1.77) | 0.018 |
| 2015 – 2019 | -19.34 (-29.15, -9.53) | <0.001 |
| AE conditions |  |  |
| None (reference) | 0.00 | — |
| One | 30.48 (25.89, 35.06) | <0.001 |
| Two or more | 73.65 (67.45, 79.85) | <0.001 |
| Background consultation frequency (visits/y) | 2.10 (1.90, 2.29) | <0.001 |
| Increase in frequency |  |  |
| No Increase (reference) | 0.00 | — |
| Increase | 23.12 (18.04, 28.19) | <0.001 |
| IMD | 2.23 (0.74, 3.71) | 0.003 |
| First symptom |  |  |
| Cough (reference) | 0.00 | — |
| Dyspnoea | -7.93 (-13.88, -1.98) | 0.009 |
| Chest infection | 9.17 (3.07, 15.26) | 0.003 |
| Chest pain | -16.75 (-24.63, -8.87) | <0.001 |
| Fatigue | -8.48 (-17.57, 0.61) | 0.067 |
| Thrombocytosis | -28.23 (-37.94, -18.53) | <0.001 |
| Multiple | -15.20 (-25.38, -5.03) | 0.003 |
| Haemoptysis | -82.77 (-93.72, -71.81) | <0.001 |
| Weight loss | -51.31 (-63.21, -39.41) | <0.001 |
| Other | -62.78 (-81.61, -43.94) | <0.001 |

Table S5 Results of linear regression model with increase in consultation frequency added as a continuous variable.

|  | Model 1  (n = 11870) | p |
| --- | --- | --- |
| Predictor |  |  |
| Sex (female) | 6.92 (2.96, 10.87) | <0.001 |
| Age at diagnosis (y) | 0.311 (0.049, 0.573) | 0.020 |
| Diagnosis year |  |  |
| <2000 (reference) | 0.00 | — |
| 2000 – 2004 | -12.59 (-21.04, -4.15) | 0.003 |
| 2005 – 2009 | -13.10 (-21.31, -4.89) | 0.002 |
| 2010 – 2014 | -13.83 (-22.62, -5.40) | 0.001 |
| 2015 – 2019 | -23.22 (-32.71, -13.74) | <0.001 |
| AE conditions |  |  |
| None (reference) | 0.00 | — |
| One | 29.00 (24.59, 33.41) | <0.001 |
| Two or more | 71.87 (65.90, 77.85) | <0.001 |
| Background consultation frequency (visits/y) | 2.31 (2.13, 2.50) | <0.001 |
| Increase in frequency | 1.88 (1.68, 2.08) | <0.001 |
| IMD | 1.99 (0.56, 3.41) | 0.006 |
| First symptom |  |  |
| Cough (reference) | 0.00 | — |
| Dyspnoea | -7.92 (-13.66, -2.19) | 0.007 |
| Chest infection | 8.21 (2.34, 14.08) | 0.006 |
| Chest pain | -19.06 (-26.71, -11.43) | <0.001 |
| Fatigue | -10.19 (-18.92, -1.47) | 0.022 |
| Thrombocytosis | -32.17 (-41.48, -22.86) | <0.001 |
| Multiple | -16.15 (-25.98, -6.31) | 0.001 |
| Haemoptysis | -80.99 (-91.67, -70.30) | <0.001 |
| Weight loss | -50.59 (-61.83, -39.36) | <0.001 |
| Other | -61.42 (-79.25, -43.59) | <0.001 |

Table S6. Results of multiple linear regression model for diagnostic interval in days with imputed data for missing values for BMI, smoking status and alcohol drinking

|  | Model 3  (n = 11870) |  |
| --- | --- | --- |
| Predictor | Adjusted B (95% CI) in days | p |
| Sex (female) | 5.68 (1.58, 9.79) | 0.007 |
| Age at diagnosis (y) | 0.348 (0.078, 0.618) | 0.012 |
| Diagnosis year |  |  |
| <2000 | 0.00 | — |
| 2000 – 2004 | -13.42 (-21.96, -4.87) | 0.002 |
| 2005 – 2009 | -12.30 (-20.69, -3.91) | 0.004 |
| 2010 – 2014 | -9.47 (-18.10, -0.85) | 0.031 |
| 2015 – 2019 | -18.74 (-28.43, -9.04) | <0.001 |
| AE conditions |  |  |
| None (reference) | 0.00 | — |
| One | 29.87 (25.41, 34.32) | <0.001 |
| Two or more | 72.57 (66.51, 78.63) | <0.001 |
| Background consultation frequency (visits/y) | 2.02 (1.83, 2.21) | <0.001 |
| Increase in frequency |  |  |
| No Increase (reference) | 0.00 | — |
| Increase | 23.00 (18.07, 27.93) | <0.001 |
| IMD | 1.42 (-0.02, 2.87) | 0.054 |
| First symptom |  |  |
| Cough (reference) | 0.00 | — |
| Dyspnoea | -8.22 (-14.00, -2.43) | 0.005 |
| Chest infection | 8.51 (2.59, 14.44) | 0.005 |
| Chest pain | -18.34 (-26.06, -10.63) | <0.001 |
| Fatigue | -10.00 (-18.81, -1.20) | 0.026 |
| Thrombocytosis | -30.21 (-39.61, -20.81) | <0.001 |
| Multiple | -18.70 (-28.62, -8.78) | <0.001 |
| Haemoptysis | -83.27 (-94.06, -72.47) | <0.001 |
| Weight loss | -53.15 (-64.51, -41.80) | <0.001 |
| Other | -62.45 (-80.45, -44.45) | <0.001 |
|  |  |  |
| BMI Category |  |  |
| Healthy weight (reference) | 0.00 | — |
| Underweight | 23.84 (12.30, 35.38) | <0.001 |
| Overweight | -4.10 (-8.63, 0.43) | 0.076 |
| Obese | -2.62 (-8.68, 3.45) | 0.396 |
| Smoking status |  |  |
| Ever smoker (reference) | 0.00 | — |
| Non-smoker | -10.31 (-17.01, -3.60) | 0.003 |
| Alcohol drinking^h^ |  |  |
| Current drinker (reference) | 0.00 | — |
| Former drinker | 6.61 (-0.60, 13.81) | 0.072 |
| Non drinker | 7.95 (1.27, 14.63) | 0.020 |

Table S7. Results of linear regression model with interval from presentation in primary care to chest x-ray as the dependent variable.

|  | Model 1  (n = 5884) | p |
| --- | --- | --- |
| Predictor |  |  |
| Sex (female) | 11.96 (6.86, 17.05) | <0.001 |
| Age at diagnosis (y) | 0.315 (-0.026, 0.655) | 0.070 |
| Diagnosis year |  |  |
| <2000 (reference) | 0.00 | — |
| 2000 – 2004 | -6.20 (-17.53, 5.13) | 0.284 |
| 2005 – 2009 | -7.61 (-18.65, 3.42) | 0.176 |
| 2010 – 2014 | -12.48 (-23.71, -1.26) | 0.029 |
| 2015 – 2019 | -23.06 (-35.69, -10.42) | <0.001 |
| AE conditions |  |  |
| None (reference) | 0.00 | — |
| One | 21.78 (16.11, 27.43) | <0.001 |
| Two or more | 60.73 (52.86, 68.59) | <0.001 |
| Background consultation frequency (visits/y) | 1.12 (0.86, 1.38) | <0.001 |
| Increase in frequency | 17.47 (10.87, 24.07) | <0.001 |
| First symptom |  |  |
| Cough (reference) | 0.00 | — |
| Dyspnoea | -7.27 (-14.50, -0.02) | 0.049 |
| Chest infection | 16.84 (9.50, 24.18) | <0.001 |
| Chest pain | -9.85 (-19.44, -0.26) | 0.044 |
| Fatigue | 27.09 (14.55, 39.62) | <0.001 |
| Thrombocytosis | 5.42 (-8.60, 19.44) | 0.449 |
| Multiple | -14.84 (-26.63, -3.04) | 0.014 |
| Haemoptysis | -74.27 (-86.59, -61.94) | <0.001 |
| Weight loss | -45.59 (-60.31, -30.87) | <0.001 |
| Other | -29.57 (-56.60, -2.55) | 0.032 |
