# Supplementary material for: The effect of comorbidities on diagnostic interval for lung cancer in England: a cohort study using electronic health record data
Source: Br J Cancer. 2024 Aug 23;131(7):1147–57. doi: 10.1038/s41416-024-02824-2 (PMC11442666; doi:10.1038/s41416-024-02824-2)
Supplement: Supplementary file 2 — Clinical Code Lists [file 41416_2024_2824_MOESM2_ESM.docx]

Lung cancer Read Codes

| medcode | readcode | readterm |  |
| --- | --- | --- | --- |
| 3903 | B22z.00 | Malignant neoplasm of bronchus or lung NOS | |
| 2587 | B22z.11 | Lung cancer | |
| 13243 | B22..00 | Malignant neoplasm of trachea, bronchus and lung | |
| 25886 | B222100 | Malignant neoplasm of upper lobe of lung | |
| 12870 | B221.00 | Malignant neoplasm of main bronchus | |
| 10358 | B222.00 | Malignant neoplasm of upper lobe, bronchus or lung | |
| 12582 | B224100 | Malignant neoplasm of lower lobe of lung | |
| 31188 | B224.00 | Malignant neoplasm of lower lobe, bronchus or lung | |
| 21698 | B221z00 | Malignant neoplasm of main bronchus NOS | |
| 21770 | BBPX.00 | [M]Mesothelioma, unspecified | |
| 16723 | BB5S212 | [M]Bronchiolar carcinoma | |
| 31700 | B222000 | Malignant neoplasm of upper lobe bronchus | |
| 38961 | B22y.00 | Malignant neoplasm of other sites of bronchus or lung | |
| 33444 | B221100 | Malignant neoplasm of hilus of lung | |
| 39923 | B223100 | Malignant neoplasm of middle lobe of lung | |
| 20170 | B222.11 | Pancoast's syndrome | |
| 31268 | B223.00 | Malignant neoplasm of middle lobe, bronchus or lung | |
| 44169 | B222z00 | Malignant neoplasm of upper lobe, bronchus or lung NOS | |
| 18678 | B224000 | Malignant neoplasm of lower lobe bronchus | |
| 42569 | B2zz.00 | Malignant neoplasm of respiratory tract NOS | |
| 42566 | B224z00 | Malignant neoplasm of lower lobe, bronchus or lung NOS | |
| 9156 | BB1K.00 | [M]Oat cell carcinoma | |
| 34015 | BB5S200 | [M]Bronchiolo-alveolar adenocarcinoma | |
| 15221 | B220.00 | Malignant neoplasm of trachea | |
| 31573 | B23..00 | Malignant neoplasm of pleura | |
| 17391 | B221000 | Malignant neoplasm of carina of bronchus | |
| 40595 | Byu2000 | [X]Malignant neoplasm of bronchus or lung, unspecified | |
| 36371 | B225.00 | Malignant neoplasm of overlapping lesion of bronchus & lung | |
| 36530 | BB5S211 | [M]Alveolar cell carcinoma | |
| 41523 | B223000 | Malignant neoplasm of middle lobe bronchus | |
| 34742 | B23z.00 | Malignant neoplasm of pleura NOS | |
| 54134 | B223z00 | Malignant neoplasm of middle lobe, bronchus or lung NOS | |
| 37810 | B220z00 | Malignant neoplasm of trachea NOS | |
| 29283 | B2zy.00 | Malignant neoplasm of other site of respiratory tract | |
| 67970 | BB1L.00 | [M]Small cell carcinoma, fusiform cell type | |
| 103946 | B220100 | Malignant neoplasm of mucosa of trachea | |

Lung Cancer ICD-10 codes

| C33 |
| --- |
| C34.0 |
| C34.1 |
| C34.2 |
| C34.3 |
| C34.8 |
| C34.9 |
| D38.1 |

Smoking Read Codes

| medcode | readterm | smokstatus |
| --- | --- | --- |
| 33 | Never smoked tobacco | non-smoker |
| 60 | Current non-smoker | non-smoker |
| 90 | Ex smoker | ex-smoker |
| 93 | Cigarette smoker | current smoker |
| 776 | Stopped smoking | ex-smoker |
| 1822 | Very heavy smoker - 40+cigs/d | current smoker |
| 1823 | Smoker | current smoker |
| 1878 | Moderate smoker - 10-19 cigs/d | current smoker |
| 2111 | Health ed. - smoking | current smoker |
| 3568 | Heavy smoker - 20-39 cigs/day | current smoker |
| 6359 | Nicotine withdrawal | current or ex-smoker |
| 7622 | Smoking cessation advice | current or ex-smoker |
| 9045 | Advice on smoking | current smoker |
| 9833 | Nicotine replacement therapy | current or ex-smoker |
| 10184 | Pregnancy smoking advice | current smoker |
| 10211 | Smoking cessation milestones | ex-smoker |
| 10558 | Current smoker | current smoker |
| 10742 | Referral to stop-smoking clinic | current or ex-smoker |
| 10898 | Smoking free weeks | ex-smoker |
| 11356 | Seen by smoking cessation advisor | current or ex-smoker |
| 11527 | DNA - Did not attend smoking cessation clinic | current smoker |
| 11713 | Pack years | current or ex-smoker |
| 11788 | Non-smoker | non-smoker |
| 12240 | Trying to give up smoking | current or ex-smoker |
| 12878 | Date ceased smoking | ex-smoker |
| 12941 | Occasional smoker | current smoker |
| 12942 | Smoker - amount smoked | current smoker |
| 12943 | Cigar smoker | current smoker |
| 12944 | Light smoker - 1-9 cigs/day | current smoker |
| 12945 | Rolls own cigarettes | current smoker |
| 12946 | Ex-smoker - amount unknown | ex-smoker |
| 12947 | Pipe smoker | current smoker |
| 12951 | Smoking restarted | current smoker |
| 12952 | Smoking started | current smoker |
| 12953 | Attends stop smoking monitor. | current or ex-smoker |
| 12954 | [V]Tobacco use | current smoker |
| 12955 | Ex-moderate smoker (10-19/day) | ex-smoker |
| 12956 | Ex-heavy smoker (20-39/day) | ex-smoker |
| 12957 | Ex-light smoker (1-9/day) | ex-smoker |
| 12958 | Trivial smoker - < 1 cig/day | current smoker |
| 12959 | Ex-very heavy smoker (40+/day) | ex-smoker |
| 12960 | Tobacco consumption NOS | current smoker |
| 12961 | Ex-trivial smoker (<1/day) | ex-smoker |
| 12962 | Tobacco consumption unknown | current smoker |
| 12963 | Cigar consumption | current smoker |
| 12964 | Keeps trying to stop smoking | current smoker |
| 12965 | Cigarette consumption | current smoker |
| 12966 | Smoking reduced | current smoker |
| 12967 | Pipe tobacco consumption | current smoker |
| 13351 | Passive smoker | non-smoker |
| 16717 | Smokers' cough | current smoker |
| 18573 | Referral to smoking cessation advisor | current or ex-smoker |
| 18926 | Lifestyle advice regarding smoking | current smoker |
| 19485 | Stop smoking monitor.chck done | current or ex-smoker |
| 19488 | Ex cigar smoker | ex-smoker |
| 21637 | Stop smoking monitor admin.NOS | current or ex-smoker |
| 24529 | Nicotine replacement therapy refused | current smoker |
| 25106 | Nicotine replacement therapy provided free | current or ex-smoker |
| 26470 | Ex pipe smoker | ex-smoker |
| 28834 | Anti-smoking monitoring admin. | current or ex-smoker |
| 30423 | Thinking about stopping smoking | current smoker |
| 30762 | Not interested in stopping smoking | current smoker |
| 31114 | Ready to stop smoking | current smoker |
| 32083 | Stop smoking clinic admin. | current or ex-smoker |
| 32572 | Over the counter nicotine replacement therapy | current or ex-smoker |
| 32687 | Tobacco dependence | current smoker |
| 34126 | Negotiated date for cessation of smoking | current smoker |
| 35055 | [V]Tobacco abuse counselling | current smoker |
| 38112 | Smoking cessation programme start date | current smoker |
| 40417 | Stop smoking monitor default | current or ex-smoker |
| 40418 | Refuses stop smoking monitor | current smoker |
| 41042 | Smoking cessation advice provided by community pharmacist | current or ex-smoker |
| 41979 | Smoking restarted | current smoker |
| 42288 | Pack years | current or ex-smoker |
| 42722 | Stop smoking monitor 1st lettr | current or ex-smoker |
| 43433 | Toxic effect of tobacco and nicotine | current smoker |
| 46300 | Cigarette pack-years | current or ex-smoker |
| 46321 | Reason for restarting smoking | current smoker |
| 47273 | Motives for smoking scale | current smoker |
| 49418 | RFS - Reasons for smoking scale | current smoker |
| 52503 | No smokers in the household | non-smoker |
| 53101 | Stop smoking monitor verb.inv. | current or ex-smoker |
| 56144 | [X]Mental and behav dis due to use of tobacco: harmful use | current smoker |
| 57639 | Bupropion refused | current smoker |
| 58597 | Stop smoking monitor phone inv | current or ex-smoker |
| 59866 | Reasons for smoking scale | current smoker |
| 60720 | Stop smoking monitor 2nd lettr | current or ex-smoker |
| 61905 | [X]Mental and behavioural disorder due to use of tobacco | current smoker |
| 62686 | Minutes from waking to first tobacco consumption | current smoker |
| 63016 | [X]Bupropion causing adverse effects in therapeutic use | current or ex-smoker |
| 63299 | FTND - Fagerstrom test for nicotine dependence | current smoker |
| 63666 | Fagerstrom test for nicotine dependence | current smoker |
| 63717 | Bupropion contraindicated | current or ex-smoker |
| 63901 | Stop smoking monitoring delete | current or ex-smoker |
| 66387 | Stop smoking monitor 3rd lettr | current or ex-smoker |
| 66409 | Nicotine replacement therapy contraindicated | current or ex-smoker |
| 67178 | Nicotine replacement therapy provided by community pharmacis | current or ex-smoker |
| 68658 | Tobacco dependence NOS | current smoker |
| 70746 | Tobacco dependence, continuous | current smoker |
| 72700 | [V]Personal history of tobacco abuse | current or ex-smoker |
| 72706 | Tobacco dependence in remission | ex-smoker |
| 74907 | Smoking cessation therapy | current or ex-smoker |
| 81440 | Nicotine replacement therapy using nicotine patches | current or ex-smoker |
| 85247 | Nicotine replacement therapy using nicotine inhalator | current or ex-smoker |
| 85975 | Nicotine replacement therapy using nicotine gum | current or ex-smoker |
| 89464 | Nicotine replacement therapy using nicotine lozenges | current or ex-smoker |
| 90522 | Smoking cessation therapy NOS | current or ex-smoker |
| 91513 | Occasions for smoking scale | current smoker |
| 91708 | Other specified smoking cessation therapy | current or ex-smoker |
| 94958 | Smoking cessation drug therapy | current or ex-smoker |
| 95610 | Tobacco dependence, unspecified | current smoker |
| 96992 | Smoking cessation - enhanced services administration | current or ex-smoker |
| 97210 | Ex-cigarette smoker | ex-smoker |
| 97643 | Fagerstrom test for nicotine dependence | current smoker |
| 97973 | Maternal tobacco abuse | current smoker |
| 98137 | Brief intervention for smoking cessation | current or ex-smoker |
| 98154 | Referral to NHS stop smoking service | current or ex-smoker |
| 98177 | Non-smoker annual review - enhanced services administration | non-smoker |
| 98245 | Stop smoking face to face follow-up | current or ex-smoker |
| 98347 | Current smoker annual review - enhanced services admin | current smoker |
| 98447 | Ex-smoker annual review - enhanced services administration | ex-smoker |
| 98493 | Smoking cessatn monitor template complet - enhanc serv admin | current or ex-smoker |
| 99838 | Recently stopped smoking | ex-smoker |
| 100099 | Smoking cessation advice declined | current smoker |
| 100495 | Ex roll-up cigarette smoker | ex-smoker |
| 100963 | Ex-smoker annual review | ex-smoker |
| 101210 | Consent given for smoking cessation data sharing | current or ex-smoker |
| 101325 | Declin cons follow-up evaluation after smoking cess interven | current or ex-smoker |
| 101338 | Failed attempt to stop smoking | current smoker |
| 101385 | Consent given for follow-up by smoking cessation team | current or ex-smoker |
| 101634 | Consent given follow-up after smoking cessation intervention | current or ex-smoker |
| 101764 | Practice based smoking cessation programme start date | current or ex-smoker |
| 101851 | Declined consent for follow-up by smoking cessation team | current or ex-smoker |
| 101854 | Declined consent for smoking cessation data sharing | current or ex-smoker |
| 101878 | Non-smoker annual review | non-smoker |
| 102361 | Referral for smoking cessation service offered | current smoker |
| 102951 | Lost to smoking cessation follow-up | current or ex-smoker |
| 103507 | Stop smoking service opportunity signposted | current smoker |
| 104086 | Stop smoking invitation first SMS text message | current smoker |
| 104185 | Smoking cessation drug therapy declined | current smoker |
| 104230 | Smoking cessation programme declined | current smoker |
| 104310 | Current smoker annual review | current smoker |
| 105572 | Stop smoking invitation short message service text message | current smoker |
| 105710 | Smoking cessation 12 week follow-up | current or ex-smoker |
| 106359 | Referral to smoking cessation service | current smoker |
| 106384 | Stop smoking invitation second SMS text message | current smoker |
| 106385 | Stop smoking invitation third SMS text message | current smoker |
| 106391 | Referral to smoking cessation service declined | current smoker |
| 107792 | [X]Mental and behav dis due to use tobacco: dependence syndr | current smoker |
| 109716 | Issue of nicotine replacement therapy voucher | current smoker |
| 110692 | Varenicline smoking cessation therapy offered | current smoker |
| 111853 | [X]Mental & behav dis due to use tobacco: acute intoxication | current smoker |

BMI Read Codes

| medcode | readcode | readterm | category |
| --- | --- | --- | --- |
| 32914 | 22K3.00 | Body Mass Index low K/M2 | Underweight |
| 2135 | E271.00 | Anorexia nervosa | Underweight |
| 126 | 22A6.00 | O/E - Underweight | Underweight |
| 24496 | 22K6.00 | Body mass index less than 20 | Underweight |
| 8027 | 1467 | H/O: anorexia nervosa | Underweight |
| 12530 | R034800 | [D]Underweight | Underweight |
| 34929 | Eu50100 | [X]Atypical anorexia nervosa | Underweight |
| 28946 | 22K1.00 | Body Mass Index normal K/M2 | Healthy weight |
| 44291 | 22K8.00 | Body mass index 20-24 - normal | Healthy weight |
| 23376 | 22A3.00 | O/E - weight within 10% ideal | Healthy weight |
| 43472 | 66CK.00 | Target weight reached | Healthy weight |
| 52703 | 212Q.00 | Obesity resolved | Healthy weight |
| 107231 | 22KA.00 | Target body mass index | Healthy weight |
| 9015 | 22K4.00 | Body mass index index 25-29 - overweight | Overweight |
| 2839 | 22A4.11 | O/E - overweight | Overweight |
| 16404 | 22A4.00 | O/E - weight 10-20% over ideal | Overweight |
| 103499 | 22AA.00 | Overweight | Overweight |
| 102150 | 66CM.00 | Risk health associ overweight and obesity, at increased risk | Overweight |
| 102514 | 66CN.00 | Risk health associated overweight and obesity, at high risk | Overweight |
| 104724 | 66CL.00 | Risk health associa overweight obesity, at no increased risk | Overweight |
| 32974 | 22A5.00 | O/E - weight > 20% over ideal | Obese |
| 430 | C380.00 | Obesity | Obese |
| 28937 | 22K2.00 | Body Mass Index high K/M2 | Obese |
| 13278 | 22K5.00 | Body mass index 30+ - obesity | Obese |
| 11461 | 66C..00 | Obesity monitoring | Obese |
| 6713 | 8CA4011 | Patient advised to lose weight | Obese |
| 22556 | 22K7.00 | Body mass index 40+ - severely obese | Obese |
| 25951 | 8HHH.00 | Refer to weight management programme | Obese |
| 12445 | ZG53100 | Patient advised to lose weight | Obese |
| 3176 | 66C4.00 | Has seen dietician - obesity | Obese |
| 29538 | 66C2.00 | Follow-up obesity assessment | Obese |
| 8854 | C380300 | Morbid obesity | Obese |
| 38658 | 66C1.00 | Initial obesity assessment | Obese |
| 47439 | 9OKA.00 | Obesity monitoring check done | Obese |
| 40153 | 66CZ.00 | Obesity monitoring NOS | Obese |
| 17470 | 6878 | Obesity screen | Obese |
| 16196 | 1444 | H/O: obesity | Obese |
| 49409 | 9OK4.00 | Obesity monitoring 1st letter | Obese |
| 52034 | 9OK1.00 | Attends obesity monitoring | Obese |
| 22695 | C380400 | Central obesity | Obese |
| 38632 | 66C6.00 | Treatment of obesity started | Obese |
| 10728 | ZC2CM00 | Dietary advice for obesity | Obese |
| 32843 | 9OK..00 | Obesity monitoring admin. | Obese |
| 11401 | C38z000 | Simple obesity NOS | Obese |
| 17477 | ZV65319 | [V]Dietary counselling in obesity | Obese |
| 66406 | C38..00 | Obesity and other hyperalimentation | Obese |
| 67516 | 9OK2.00 | Refuses obesity monitoring | Obese |
| 52036 | 9OK3.00 | Obesity monitoring default | Obese |
| 38059 | C380200 | Extreme obesity with alveolar hypoventilation | Obese |
| 38799 | C380000 | Obesity due to excess calories | Obese |
| 25968 | C380500 | Generalised obesity | Obese |
| 24755 | C38y.11 | Pickwickian syndrome | Obese |
| 70950 | 9OK7.00 | Obesity monitoring verbal inv. | Obese |
| 52735 | 9OKZ.00 | Obesity monitoring admin.NOS | Obese |
| 67517 | 9OK8.00 | Obesity monitor phone invite | Obese |
| 103574 | C38y011 | Obesity hypoventilation syndrome | Obese |
| 21744 | 9OK..11 | Obesity clinic administration | Obese |
| 55586 | 9OK5.00 | Obesity monitoring 2nd letter | Obese |
| 17444 | 66CE.00 | Reason for obesity therapy - occupational | Obese |
| 38294 | C38y000 | Pickwickian syndrome | Obese |
| 17897 | ZV77800 | [V]Screening for obesity | Obese |
| 55585 | 9OK6.00 | Obesity monitoring 3rd letter | Obese |
| 64712 | 66C5.00 | Treatment of obesity changed | Obese |
| 102647 | 9hN0.00 | Excepted from obesity quality indicators: patient unsuitable | Obese |
| 49250 | C380100 | Drug-induced obesity | Obese |
| 69757 | Cyu7000 | [X]Other obesity | Obese |
| 70898 | C38z.00 | Obesity and other hyperalimentation NOS | Obese |
| 105638 | 9hN1.00 | Excepted from obesity quality indicators: informed dissent | Obese |
| 48330 | L161.12 | Maternal obesity syndrome | Obese |
| 103212 | 9hN..00 | Exception reporting: obesity quality indicators | Obese |
| 104421 | C380700 | Lifelong obesity | Obese |
| 106010 | 66CP.00 | Risk health associ overweight and obesity, at very high risk | Obese |
| 52782 | Cyu7.00 | [X]Obesity and other hyperalimentation | Obese |
| 104129 | C380600 | Adult-onset obesity | Obese |

Alcohol Drinking Read Codes

| medcode | readcode | readterm | drinkingstatus |
| --- | --- | --- | --- |
| 27 | 136..00 | alcohol consumption | current |
| 322 | 1364 | Moderate drinker - 3-6u/day | current |
| 385 | 1362.11 | Drinks rarely | current |
| 669 | E250000 | Nondependent alcohol abuse, unspecified | current |
| 749 | 1362.12 | Drinks occasionally | current |
| 956 | 136J.00 | Social drinker | current |
| 967 | 1367 | Stopped drinking alcohol | former |
| 1399 | E23..12 | Alcohol problem drinking | current |
| 1476 | E010.12 | Delirium tremens | current |
| 1618 | 1365 | Heavy drinker - 7-9u/day | current |
| 2081 | E23..11 | Alcoholism | current |
| 2082 | E01y000 | Alcohol withdrawal syndrome | current |
| 2083 | 8BA8.00 | Alcohol detoxification | current |
| 2084 | E23..00 | Alcohol dependence syndrome | current |
| 2689 | 136G.00 | Beer drinker | current |
| 2925 | F375.00 | Alcoholic polyneuropathy | current |
| 3216 | J611.00 | Acute alcoholic hepatitis | current |
| 3782 | E250.14 | Intoxication - alcohol | current |
| 4447 | 1361.12 | Non-drinker alcohol | never |
| 4500 | E011000 | Korsakov's alcoholic psychosis | current |
| 4506 | J153.00 | Alcoholic gastritis | current |
| 4743 | J612.00 | Alcoholic cirrhosis of liver | current |
| 4915 | G555.00 | Alcoholic cardiomyopathy | current |
| 5611 | Eu10.00 | [X]Mental and behavioural disorders due to use of alcohol | current |
| 5740 | E230.00 | Acute alcoholic intoxication in alcoholism | current |
| 5758 | Eu10212 | [X]Chronic alcoholism | current |
| 6169 | E23z.00 | Alcohol dependence syndrome NOS | current |
| 6467 | Eu10511 | [X]Alcoholic hallucinosis | current |
| 7123 | ZV11300 | [V]Personal history of alcoholism | current |
| 7545 | ZV4KC00 | [V] Alcohol use | current |
| 7602 | J617000 | Chronic alcoholic hepatitis | current |
| 7692 | 8CAM.00 | Patient advised about alcohol | current |
| 7746 | E250.00 | Nondependent alcohol abuse | current |
| 7885 | J613.00 | Alcoholic liver damage unspecified | current |
| 7943 | J617.00 | Alcoholic hepatitis | current |
| 8030 | ZV6D600 | [V]Alcohol abuse counselling and surveillance | current |
| 8363 | G852300 | Oesophageal varices in alcoholic cirrhosis of the liver | current |
| 8388 | ZV57A00 | [V]Alcohol rehabilitation | current |
| 8430 | 1462 | h/o: alcoholism | former |
| 8999 | 136P.00 | Heavy drinker | current |
| 9169 | R103.00 | [D]Alcohol blood level excessive | current |
| 9489 | 9NN2.00 | Under care of community alcohol team | current |
| 9508 | Eu10011 | [X]Acute alcoholic drunkenness | current |
| 9849 | 8H7p.00 | Referral to community alcohol team | current |
| 10161 | 2577.11 | O/E - alcoholic breath | current |
| 10458 | ZV79100 | [v]screening for alcoholism | current |
| 10463 | U209.00 | [x]intent self poison/exposure to alcohol | current |
| 10658 | ZRLfD12 | honos item 3 - alcohol/drug problem | current |
| 10691 | J610.00 | Alcoholic fatty liver | current |
| 11106 | E011100 | Korsakov's alcoholic psychosis with peripheral neuritis | current |
| 11140 | ZG23100 | Advice on alcohol consumption | current |
| 11263 | SM01100 | wood alcohol causing toxic effect | current |
| 11491 | 6792 | Health ed. - alcohol | current |
| 11670 | Eu10611 | [X]Korsakov's psychosis, alcohol induced | current |
| 11740 | 9k1..00 | Alcohol misuse - enhanced services administration | current |
| 12271 | E250.11 | Drunkenness NOS | current |
| 12353 | Eu10500 | [X]Mental & behav dis due to use alcohol: psychotic disorder | current |
| 12442 | 66e..00 | alcohol disorder monitoring | current |
| 12554 | 8HHe.00 | Referral to community drug and alcohol team | current |
| 12949 | 1361 | Teetotaller | never |
| 12950 | U800.00 | [x]eviden of alcohl involv blood alcohl level <20 mg/100 ml | current |
| 12968 | 136H.00 | Drinks beer and spirits | current |
| 12969 | 136I.00 | Drinks wine | current |
| 12970 | 1361.11 | Non drinker alcohol | never |
| 12971 | 136F.00 | Spirit drinker | current |
| 12972 | 1363 | Light drinker - 1-2u/day | current |
| 12974 | E250200 | Nondependent alcohol abuse, episodic | current |
| 12975 | 1362 | Trivial drinker - <1u/day | current |
| 12976 | 1369 | Suspect alcohol abuse - denied | current |
| 12977 | 1366 | Very heavy drinker - >9u/day | current |
| 12978 | 1368 | alcohol consumption unknown | current |
| 12979 | 136M.00 | Current non drinker | never |
| 12980 | 136N.00 | Light drinker | current |
| 12981 | 136Z.00 | alcohol consumption nos | current |
| 12982 | 136K.00 | Alcohol intake above recommended sensible limits | current |
| 12983 | 136E.00 | Ex-very heavy drinker-(>9u/d) | former |
| 12984 | 136Q.00 | Very heavy drinker | current |
| 12985 | 136O.00 | Moderate drinker | current |
| 15503 | T901.00 | accidental poisoning by other ethyl alcohol and its products | current |
| 16225 | E010.00 | Alcohol withdrawal delirium | current |
| 16237 | E01..00 | Alcoholic psychoses | current |
| 16587 | ZV11311 | [V]Problems related to lifestyle alcohol use | current |
| 17149 | ZV70L00 | [v]blood-alcohol and blood-drug test | current |
| 17259 | Eu10411 | [X]Delirium tremens, alcohol induced | current |
| 17266 | 63C7.00 | maternal alcohol abuse | current |
| 17330 | J613000 | Alcoholic hepatic failure | current |
| 17607 | Eu10514 | [X]Alcoholic psychosis NOS | current |
| 17777 | E250.13 | Inebriety NOS | current |
| 18156 | 13Y8.00 | Alcoholics anonymous | current |
| 18252 | T90..00 | accidental poisoning by alcohol, nec | current |
| 18636 | E011200 | Wernicke-Korsakov syndrome | current |
| 18711 | 67H0.00 | Lifestyle advice regarding alcohol | current |
| 19217 | SM0..00 | Alcohol causing toxic effect | current |
| 19401 | 136R.00 | Binge drinker | current |
| 19489 | 6892 | alcohol consumption screen | current |
| 19493 | 136D.00 | Ex-heavy drinker - (7-9u/day) | former |
| 19494 | 136S.00 | Hazardous alcohol use | current |
| 19495 | 136C.00 | Ex-moderate drinker - (3-6u/d) | former |
| 20407 | E014.11 | Drunkenness - pathological | current |
| 20514 | Eu10300 | [X]Mental and behav dis due to use alcohol: withdrawal state | current |
| 20762 | E011.00 | Alcohol amnestic syndrome | current |
| 21412 | TJH3.00 | adverse reaction to alcohol deterrents | current |
| 21624 | E230200 | Episodic acute alcoholic intoxication in alcoholism | current |
| 21650 | 8H35.00 | Admitted to alcohol detoxification centre | current |
| 21713 | J612000 | Alcoholic fibrosis and sclerosis of liver | current |
| 21879 | Eu10100 | [X]Mental and behav dis due to use of alcohol: harmful use | current |
| 22277 | E010.11 | DTs - delirium tremens | current |
| 22707 | ZRBJ.00 | Drinking problem scale | current |
| 22933 | 136A.00 | Ex-trivial drinker (<1u/day) | former |
| 23610 | E250100 | Nondependent alcohol abuse, continuous | current |
| 23978 | U81..00 | [X]Evid of alcohol involv determind by level of intoxication | current |
| 24064 | E231100 | Continuous chronic alcoholism | current |
| 24485 | E231300 | Chronic alcoholism in remission | former |
| 24735 | 2577 | O/E - breath - alcohol smell | current |
| 24984 | J671000 | Alcohol-induced chronic pancreatitis | current |
| 25110 | E013.00 | Alcohol withdrawal hallucinosis | current |
| 26106 | E231200 | Episodic chronic alcoholism | current |
| 26323 | Eu10711 | [X]Alcoholic dementia NOS | current |
| 26471 | 136B.00 | Ex-light drinker - (1-2u/day) | former |
| 26472 | 136L.00 | Alcohol intake within recommended sensible limits | current |
| 27342 | E012.11 | Alcoholic dementia NOS | current |
| 27518 | E250.12 | Hangover (alcohol) | current |
| 27670 | L255300 | Maternal care for (suspected) damage to fetus from alcohol | current |
| 28150 | E250z00 | Nondependent alcohol abuse NOS | current |
| 28780 | Eu10211 | [X]Alcohol addiction | current |
| 29691 | 8G32.00 | Aversion therapy - alcoholism | current |
| 30036 | U409.00 | [x]poisoning/exposure, ? intent, to alcohol | current |
| 30162 | Eu10513 | [X]Alcoholic paranoia | current |
| 30404 | E015.00 | Alcoholic paranoia | current |
| 30460 | Z4B1.00 | Alcoholism counselling | current |
| 30604 | F25B.00 | Alcohol-induced epilepsy | current |
| 30695 | 136T.00 | Harmful alcohol use | current |
| 31443 | E231.00 | Chronic alcoholism | current |
| 31569 | E250300 | Nondependent alcohol abuse in remission | current |
| 31605 | U1A9.00 | [x]accident poisoning/exposure to alcohol | current |
| 31742 | F394100 | Alcoholic myopathy | current |
| 32454 | U806.00 | [x]eviden of alcoh involv blood alcoh level 120-199mg/100ml | current |
| 32850 | ZR1F.00 | alcohol use disorders identification test | current |
| 32927 | Eu10800 | [X]Alcohol withdrawal-induced seizure | current |
| 32964 | 66e0.00 | alcohol abuse monitoring | current |
| 33635 | E231z00 | Chronic alcoholism NOS | current |
| 33670 | E01y.00 | Other alcoholic psychosis | current |
| 33839 | F144000 | Cerebellar ataxia due to alcoholism | current |
| 35330 | 9k11.00 | Alcohol consumption counselling | current |
| 35859 | 67A5.00 | pregnancy alcohol advice | current |
| 36296 | E230z00 | Acute alcoholic intoxication in alcoholism NOS | current |
| 36499 | SM0z.00 | alcohol causing toxic effect nos | current |
| 36687 | SLH3.00 | alcohol deterrent poisoning | current |
| 36748 | F11x011 | Alcoholic encephalopathy | current |
| 37174 | T902.00 | accidental poisoning by methyl alcohol | current |
| 37264 | 8CE1.00 | Alcohol leaflet given | current |
| 37605 | E231.11 | Dipsomania | current |
| 37691 | Eu10712 | [X]Chronic alcoholic brain syndrome | current |
| 37946 | E012000 | Chronic alcoholic brain syndrome | current |
| 38061 | 1B1c.00 | Alcohol induced hallucinations | current |
| 39327 | Eu10200 | [X]Mental and behav dis due to use alcohol: dependence syndr | current |
| 39726 | U808.00 | [x]eviden alcoh involv blood alcoh level 240mg/100ml or more | current |
| 39738 | U813.00 | [x]evid alcoh invl determ by levl intox very sev alcoh intox | current |
| 39799 | Eu10600 | [X]Mental and behav dis due to use alcohol: amnesic syndrome | current |
| 40530 | E230000 | Acute alcoholic intoxication, unspecified, in alcoholism | current |
| 40541 | T900.00 | accidental poisoning by alcoholic beverages | current |
| 40602 | ZR1E.11 | ads - alcohol dependence scale | current |
| 41251 | ZRa1111 | bmast - brief michigan alcoholism screening test | current |
| 41638 | U209000 | [x]int self poison/exposure to alcohol at home | current |
| 41920 | E011z00 | Alcohol amnestic syndrome NOS | current |
| 41983 | Z191.00 | Alcohol detoxification | current |
| 42305 | ZRk6.00 | severity of alcohol dependence questionnaire | current |
| 43193 | E231000 | Unspecified chronic alcoholism | current |
| 43813 | 9EQ..11 | police:venesect-alcohol | current |
| 44019 | U80..00 | [x]evidence of alcohl involv determin by blood alcohl level | current |
| 44299 | Eu10000 | [X]Mental & behav dis due to use alcohol: acute intoxication | current |
| 44686 | U812.00 | [x]evid alcoh invol determ by level of intox sev alcoh intox | current |
| 44783 | 1D19.00 | Pain in lymph nodes after alcohol consumption | current |
| 45169 | Eu10y00 | [X]Men & behav dis due to use alcohol: oth men & behav dis | current |
| 45557 | ZR31.00 | cage questionnaire | current |
| 46677 | Z191100 | Alcohol withdrawal regime | current |
| 46848 | ZRBJ.11 | DPS - Drinking problem scale | current |
| 47123 | 9k14.00 | Alcohol counselling by other agencies | current |
| 47555 | F11x000 | Cerebral degeneration due to alcoholism | current |
| 47907 | U807.00 | [x]eviden of alcoh involv blood alcoh level 200-239mg/100ml | current |
| 48241 | U60H311 | [x] adverse reaction to alcohol deterrents | current |
| 48514 | SM00100 | denatured alcohol causing toxic effect | current |
| 48545 | 9k13.00 | alcohol questionnaire completed | current |
| 50507 | ZR1E.00 | alcohol dependence scale | current |
| 53139 | T90y.00 | accidental poisoning by other alcohols | current |
| 53428 | U804.00 | [x]eviden of alcohl involv blood alcohl level 80-99mg/100ml | current |
| 54209 | ZC2H.00 | Advice to change alcohol intake | current |
| 54504 | U805.00 | [x]eviden of alcoh involv blood alcoh level 100-119mg/100ml | current |
| 54505 | E012.00 | Other alcoholic dementia | current |
| 55415 | T90z.00 | accidental poisoning by alcohol nos | current |
| 55536 | SM00z00 | ethyl alcohol causing toxic effect nos | current |
| 56410 | 7P22100 | Delivery of rehabilitation for alcohol addiction | current |
| 56441 | ZRa1.00 | michigan alcoholism screening test | current |
| 56947 | E230100 | Continuous acute alcoholic intoxication in alcoholism | current |
| 57202 | U801.00 | [x]eviden of alcohl involv blood alcohl level 20-39mg/100ml | current |
| 57242 | U803.00 | [x]eviden of alcohl involv blood alcohl level 60-79mg/100ml | current |
| 57714 | E230.11 | Alcohol dependence with acute alcoholic intoxication | current |
| 57939 | E014.00 | Pathological alcohol intoxication | current |
| 59079 | U802.00 | [x]eviden of alcohl involv blood alcohl level 40-59mg/100ml | current |
| 59414 | U209z00 | [x]intent self poison alcohol unspecif place | current |
| 59574 | E230300 | Acute alcoholic intoxication in remission, in alcoholism | current |
| 59776 | ZRVK.00 | last six months of drinking questionnaire | current |
| 59873 | ZR1F.11 | audit - alcohol use disorders identification test | current |
| 60752 | T903300 | accidental poisoning by secondary propyl alcohol | current |
| 61187 | U1A9z00 | [x]accid poison/expos to alcohol unspecif place | current |
| 61190 | U409z00 | [x]pois/expos ?intent to alcohol unspecif place | current |
| 61383 | Z191200 | Planned reduction of alcohol consumption | current |
| 61583 | ZV70411 | [v]medicolegal blood alcohol test | current |
| 61750 | ZR3f.00 | comprehensive drinker profile | current |
| 62000 | Eu10700 | [X]Men & behav dis due alcoh: resid & late-onset psychot dis | current |
| 62299 | ZRk9.11 | sadd - short alcohol dependence data | current |
| 62300 | ZRk9.00 | short alcohol dependence data | current |
| 63306 | U1A9000 | [x]accident poison/exposure to alcohol at home | current |
| 63457 | ZR3f.11 | cdp - comprehensive drinker profile | current |
| 63529 | 9k12.00 | Alcohol misuse - enhanced service completed | current |
| 63876 | U1A9400 | [x]accid poison/expos alcohol in street/highway | current |
| 64101 | Eu10400 | [X]Men & behav dis due alcohl: withdrawl state with delirium | current |
| 64389 | Eu10z00 | [X]Ment & behav dis due use alcohol: unsp ment & behav dis | current |
| 64396 | SM03000 | amyl alcohol causing toxic effect | current |
| 64409 | Z191400 | Self-monitoring of alcohol intake | current |
| 65754 | C150500 | Alcohol-induced pseudo-Cushing's syndrome | current |
| 65932 | Eu10512 | [X]Alcoholic jealousy | current |
| 65980 | ZR1G.00 | alcohol use inventory | current |
| 66019 | L254.11 | Suspect fetal damage from maternal alcohol | current |
| 66552 | ZRR..00 | inventory of drinking situations | current |
| 66699 | PK83.00 | Fetus and newborn affected by maternal use of alcohol | current |
| 66831 | ZRk6.11 | sadq - severity of alcohol dependence questionnaire | current |
| 67651 | E01z.00 | Alcoholic psychosis NOS | current |
| 68111 | E01yz00 | Other alcoholic psychosis NOS | current |
| 68159 | U409000 | [x]poison/exposure ?intent, to alcohol at home | current |
| 69331 | ZRa1.11 | mast - michigan alcoholism screening test | current |
| 69407 | U409200 | [x]pois/exp ?intent alcohol school/pub admin area | current |
| 69691 | Eu10213 | cage questionnaire | current |
| 70161 | 388j.00 | cage questionnaire | current |
| 70939 | ZRaU.00 | munich alcoholism test | current |
| 72757 | Q007100 | fetus/neonate affected by placental/breast transfer alcohol | current |
| 73480 | U811.00 | [x]evid alcoh invol determ by level of intox mod alcoh intox | current |
| 73876 | U60H300 | [x]alcohol deterrents caus adverse effects in therapeut use | current |
| 84218 | 13ZY.00 | Disqualified from driving due to excess alcohol | current |
| 88997 | SM01.00 | methyl alcohol causing toxic effect | current |
| 90714 | 9k15.00 | alcohol screen - audit completed | current |
| 92908 | U1A9500 | [x]accid poison/expos alcohol trade/service area | current |
| 93415 | 136V.00 | alcohol units per week | current |
| 93624 | 388u.00 | fast alcohol screening test | current |
| 94485 | 9k18.00 | alcohol screen - audit pc completed | current |
| 94553 | 8HkG.00 | Referral to specialist alcohol treatment service | current |
| 94669 | 8IA7.00 | alcohol consumption screening test declined | current |
| 94670 | 136W.00 | Alcohol misuse | current |
| 94838 | 38D3.00 | alcohol use disorders identification test | current |
| 94963 | 9k16.00 | alcohol screen - fast alcohol screening test completed | current |
| 95181 | Z191211 | Alcohol reduction programme | current |
| 95410 | SyuG000 | [x]toxic effect of other alcohols | current |
| 95650 | ZC22100 | Advice to change drink intake | current |
| 95663 | 9k17.00 | alcohol screen - audit c completed | current |
| 95744 | 38D4.00 | alcohol use disorder identificatn test consumptn questionnre | current |
| 95944 | 9k19.00 | alcohol assesment declined - enhanced services admin | current |
| 96053 | 9k1A.00 | Brief intervention for excessive alcohol consumptn completed | current |
| 96054 | 9k1B.00 | Extended intervention for excessive alcohol consumptn complt | current |
| 96107 | 38D2.00 | single alcohol screening questionnaire | current |
| 96219 | U409400 | [x]pois/expos ?intent alcohol in street/highway | current |
| 96259 | 9k19.11 | alcohol assessment declined | current |
| 96993 | 8HkJ.00 | Referral to alcohol brief intervention service | current |
| 97085 | U209y00 | [x]int self poison alcohol other spec place | current |
| 97126 | 136X.00 | alcohol units consumed on heaviest drinking day | current |
| 97163 | ZC22200 | Advice to change alcoholic drink intake | current |
| 97261 | 8IAF.00 | Brief intervention for excessive alcohol consumpion declined | current |
| 97309 | 8CAv.00 | Advised to contact primary care alcohol worker | current |
| 97501 | 38D5.00 | alcoh use disor id test piccinelli consumption questionnaire | current |
| 97680 | 8IAJ.00 | Declined referral to specialist alcohol treatment service | current |
| 99877 | 136b.00 | Feels should cut down drinking | current |
| 99985 | ZRa1100 | brief michigan alcoholism screening test | current |
| 100493 | 38Df.00 | five-shot questionnaire on heavy drinking | current |
| 100989 | 4I91.11 | breath alcohol level | current |
| 101426 | T901z00 | accidental poisoning by ethyl alcohol nos | current |
| 101543 | SM0y.00 | other alcohol causing toxic effect | current |
| 101718 | 136Y.00 | Drinks in morning to get rid of hangover | current |
| 102086 | SM02200 | rubbing alcohol causing toxic effect | current |
| 102121 | 9EQ..12 | police:venesect-alcohol | current |
| 102247 | 8IAt.00 | Extended interven for excessive alcohol consumption declined | current |
| 102321 | SM03100 | butyl alcohol causing toxic effect | current |
| 102448 | 136c.00 | Higher risk drinking | current |
| 102564 | 8CAM000 | Advised to abstain from alcohol consumption | current |
| 102577 | 38Dz.00 | severity of alcohol dependence questionnaire | current |
| 102665 | 136a.00 | Increasing risk drinking | current |
| 102770 | 38Dz.11 | sadq - severity of alcohol dependence questionnaire | current |
| 103069 | U1A9200 | [x]acc poison/expos alcohol school/pub admin area | current |
| 103230 | 136d.00 | Lower risk drinking | current |
| 103459 | 8IEA.00 | Referral to community alcohol team declined | current |
| 103698 | U80z.00 | [x]evid alcoh invol detrm by pres alcoh in bld levl not spec | current |
| 104109 | ZC67111 | Drinks only | current |
| 104611 | J670800 | Alcohol-induced acute pancreatitis | current |
| 104702 | U1A9300 | [x]accid pois/expos alcohol in sport/athletic area | current |
| 104734 | U1A9y00 | [x]accid pois/expos to alcohol other spec place | current |
| 105144 | 67K6.00 | cycle of change stage, alcohol | current |
| 105273 | U810.00 | [x]evid alcoh invol determ by levl of intox mild alcoh intox | current |
| 106018 | SM02.00 | isopropyl alcohol causing toxic effect | current |
| 106560 | 136e.00 | declines to state current alcohol consumption | current |
| 108644 | 9NJz.00 | In-house alcohol detoxification | current |
| 109105 | 8W2..00 | Refer to MH services deferred until alcohol misuse resolved | current |
| 109108 | 2126C00 | Alcohol dependence resolved | former |
| 109241 | 8IH4.00 | Alcohol Use Disorders Identification Test declined | current |
| 109668 | 8CdK.00 | Specialist alcohol treatment service signposted | current |
| 109675 | 9EVD.00 | Hospital alcohol liaison team report received | current |
| 109800 | 9NzA.00 | Hospital attendance related to personal alcohol consumption | current |
| 110382 | 9NgzH00 | Withdrawn from alcohol detoxification programme | current |
| 110494 | 8BAu.00 | Alcohol harm reduction programme | current |
| 110624 | 8BAs.00 | Alcohol relapse prevention | current |
| 110671 | Z191300 | Controlled drinking regime | current |
| 110911 | T901000 | Accidental poisoning by denatured alcohol | current |
| 111683 | Z9KF400 | Removal of alcohol | current |

Chronic Obstructive Pulmonary Disease Read Codes

| readcode | medcode | readterm |
| --- | --- | --- |
| H3...00 | 1001 | Chronic obstructive pulmonary disease |
| H31..00 | 3243 | Chronic bronchitis |
| H310.00 | 25603 | Simple chronic bronchitis |
| H310000 | 15626 | Chronic catarrhal bronchitis |
| H310z00 | 61118 | Simple chronic bronchitis NOS |
| H311.00 | 11150 | Mucopurulent chronic bronchitis |
| H311000 | 40159 | Purulent chronic bronchitis |
| H311100 | 37959 | Fetid chronic bronchitis |
| H311z00 | 61513 | Mucopurulent chronic bronchitis NOS |
| H312.00 | 27819 | Obstructive chronic bronchitis |
| H312000 | 5798 | Chronic asthmatic bronchitis |
| H312011 | 5909 | Chronic wheezy bronchitis |
| H312100 | 14798 | Emphysematous bronchitis |
| H312300 | 26125 | Bronchiolitis obliterans |
| H312z00 | 44525 | Obstructive chronic bronchitis NOS |
| H313.00 | 24248 | Mixed simple and mucopurulent chronic bronchitis |
| H31y.00 | 66043 | Other chronic bronchitis |
| H31y100 | 45089 | Chronic tracheobronchitis |
| H31yz00 | 68066 | Other chronic bronchitis NOS |
| H31z.00 | 15157 | Chronic bronchitis NOS |
| H32..00 | 794 | Emphysema |
| H320.00 | 26306 | Chronic bullous emphysema |
| H320000 | 56860 | Segmental bullous emphysema |
| H320100 | 68662 | Zonal bullous emphysema |
| H320200 | 60188 | Giant bullous emphysema |
| H320300 | 99536 | Bullous emphysema with collapse |
| H320z00 | 23492 | Chronic bullous emphysema NOS |
| H321.00 | 46578 | Panlobular emphysema |
| H322.00 | 10980 | Centrilobular emphysema |
| H32y.00 | 40788 | Other emphysema |
| H32y000 | 92955 | Acute vesicular emphysema |
| H32y100 | 70787 | Atrophic (senile) emphysema |
| H32y111 | 59263 | Acute interstitial emphysema |
| H32y200 | 63479 | MacLeod's unilateral emphysema |
| H32yz00 | 16410 | Other emphysema NOS |
| H32z.00 | 33450 | Emphysema NOS |
| H36..00 | 10863 | Mild chronic obstructive pulmonary disease |
| H37..00 | 10802 | Moderate chronic obstructive pulmonary disease |
| H38..00 | 9876 | Severe chronic obstructive pulmonary disease |
| H3y..11 | 67040 | Other specified chronic obstructive pulmonary disease |
| H3z..11 | 37247 | Chronic obstructive pulmonary disease NOS |

Asthma Read Codes

| readcode | medcode | readterm |
| --- | --- | --- |
| 173A.00 | 5867 | Exercise induced asthma |
| 173c.00 | 22752 | Occupational asthma |
| 173d.00 | 73522 | Work aggravated asthma |
| 1O2..00 | 11370 | Asthma confirmed |
| 2126200 | 10996 | Asthma resolved |
| 212G.00 | 11839 | Asthma resolved |
| 663j.00 | 10487 | Asthma - currently active |
| 663V000 | 3458 | Occasional asthma |
| 663V100 | 3018 | Mild asthma |
| 663V200 | 13065 | Moderate asthma |
| 663V300 | 3366 | Severe asthma |
| H312000 | 5798 | Chronic asthmatic bronchitis |
| H33..00 | 78 | Asthma |
| H33..11 | 1555 | Bronchial asthma |
| H330.00 | 7146 | Extrinsic (atopic) asthma |
| H330.11 | 2290 | Allergic asthma |
| H330.12 | 1208 | Childhood asthma |
| H330.13 | 15248 | Hay fever with asthma |
| H330.14 | 7731 | Pollen asthma |
| H330000 | 14777 | Extrinsic asthma without status asthmaticus |
| H330011 | 5627 | Hay fever with asthma |
| H330100 | 27926 | Extrinsic asthma with status asthmaticus |
| H330111 | 6707 | Extrinsic asthma with asthma attack |
| H330z00 | 45782 | Extrinsic asthma NOS |
| H331.00 | 5267 | Intrinsic asthma |
| H331.11 | 3665 | Late onset asthma |
| H331000 | 29325 | Intrinsic asthma without status asthmaticus |
| H331100 | 58196 | Intrinsic asthma with status asthmaticus |
| H331111 | 18323 | Intrinsic asthma with asthma attack |
| H331z00 | 45073 | Intrinsic asthma NOS |
| H332.00 | 25796 | Mixed asthma |
| H334.00 | 40823 | Brittle asthma |
| H33z.00 | 4442 | Asthma unspecified |
| H33z.11 | 32727 | Hyperreactive airways disease |
| H33z200 | 12987 | Late-onset asthma |
| H33zz00 | 16070 | Asthma NOS |
| H33zz11 | 4606 | Exercise induced asthma |
| H33zz12 | 21232 | Allergic asthma NEC |
| H35y600 | 93353 | Sequoiosis (red-cedar asthma) |
| H35y700 | 39478 | Wood asthma |
| H47y000 | 47684 | Detergent asthma |

Angiotensin-converting enzyme inhibitors

| prodcode | productname |
| --- | --- |
| 65 | Lisinopril 10mg tablets |
| 69 | Lisinopril 20mg tablets |
| 78 | Lisinopril 5mg tablets |
| 80 | Ramipril 5mg capsules |
| 82 | Ramipril 10mg capsules |
| 97 | Perindopril erbumine 4mg tablets |
| 147 | Ramipril 1.25mg capsules |
| 196 | Enalapril 5mg tablets |
| 277 | Lisinopril 2.5mg tablets |
| 448 | Enalapril 2.5mg tablets |
| 593 | Perindopril erbumine 2mg tablets |
| 633 | Fosinopril 10mg tablets |
| 654 | Ramipril 2.5/5mg/10mg capsule |
| 709 | Ramipril 2.5mg capsules |
| 756 | Ramipril 10mg tablets |
| 761 | Ramipril 1.25mg tablets |
| 1021 | Innozide 20mg/12.5mg tablets (Organon Pharma (UK) Ltd) |
| 1121 | Captopril 12.5mg tablets |
| 1143 | Captopril 25mg tablets |
| 1144 | Capoten 25mg tablets (Bristol-Myers Squibb Pharmaceuticals Ltd) |
| 1299 | Enalapril 10mg tablets |
| 1520 | Capozide 25mg/50mg tablets (Bristol-Myers Squibb Pharmaceuticals Ltd) |
| 1807 | Captopril 50mg tablets |
| 1904 | Enalapril 20mg tablets |
| 2982 | Zestoretic 20- 20mg+12.5mg Tablet (AstraZeneca UK Ltd) |
| 3069 | Acepril 25mg tablets (Bristol-Myers Squibb Pharmaceuticals Ltd) |
| 3203 | Capozide LS Tablet (E R Squibb and Sons Ltd) |
| 3310 | Capoten 12.5mg tablets (Bristol-Myers Squibb Pharmaceuticals Ltd) |
| 3720 | Zestril 2.5mg tablets (AstraZeneca UK Ltd) |
| 3839 | Capoten 50mg tablets (Bristol-Myers Squibb Pharmaceuticals Ltd) |
| 3929 | Quinapril 10mg tablets |
| 4103 | Trandolapril 1mg capsules |
| 4571 | Staril 10mg tablets (Bristol-Myers Squibb Pharmaceuticals Ltd) |
| 5047 | Trandolapril 2mg capsules |
| 5159 | Quinapril 20mg tablets |
| 5189 | Enalapril 20mg / Hydrochlorothiazide 12.5mg tablets |
| 5275 | Tritace 2.5mg capsules (Sanofi) |
| 5612 | Coversyl 2mg tablets (Servier Laboratories Ltd) |
| 5735 | Tritace 5mg capsules (Sanofi) |
| 5800 | Coversyl 4mg tablets (Servier Laboratories Ltd) |
| 5861 | Fosinopril 20mg tablets |
| 6078 | Perindopril erbumine 8mg tablets |
| 6261 | Tritace 1.25mg tablets (Sanofi) |
| 6288 | Ramipril 5mg tablets |
| 6314 | Ramipril 2.5mg tablets |
| 6359 | Zestoretic 10- 10mg+12.5mg Tablet (AstraZeneca UK Ltd) |
| 6362 | Tritace 5mg tablets (Sanofi) |
| 6364 | Tritace 2.5mg tablets (Sanofi) |
| 6408 | Tanatril 5mg tablets (Northumbria Pharma Ltd) |
| 6468 | Lisinopril 20mg / Hydrochlorothiazide 12.5mg tablets |
| 6765 | Quinapril 5mg tablets |
| 6786 | Lisinopril 10mg / Hydrochlorothiazide 12.5mg tablets |
| 6794 | Perindopril erbumine 4mg / Indapamide 1.25mg tablets |
| 6806 | Zestril 10mg tablets (Atnahs Pharma UK Ltd) |
| 6807 | Zestril 5mg tablets (Atnahs Pharma UK Ltd) |
| 7314 | Accupro 5mg tablets (Pfizer Ltd) |
| 7419 | Trandolapril 500microgram capsules |
| 8025 | Gopten 1mg capsules (Abbott Laboratories Ltd) |
| 8026 | Gopten 2mg capsules (Abbott Laboratories Ltd) |
| 8105 | Innovace 20mg tablets (Organon Pharma (UK) Ltd) |
| 8106 | Innovace 2.5mg tablets (Organon Pharma (UK) Ltd) |
| 8268 | Zestril 20mg tablets (Atnahs Pharma UK Ltd) |
| 8800 | Innovace 5mg tablets (Organon Pharma (UK) Ltd) |
| 8830 | Innovace 10mg tablets (Organon Pharma (UK) Ltd) |
| 9646 | Tritace 1.25mg capsules (Aventis Pharma) |
| 9693 | Tritace 10mg capsules (Sanofi) |
| 9731 | Quinapril 40mg tablets |
| 9764 | Carace 20 Tablet (Bristol-Myers Squibb Pharmaceuticals Ltd) |
| 9915 | Tritace 10mg tablets (Sanofi) |
| 9948 | Trandolapril 4mg capsules |
| 10882 | Carace 2.5mg tablets (Bristol-Myers Squibb Pharmaceuticals Ltd) |
| 10902 | Captopril 50mg with Hydrochlorothiazide 25mg tablets |
| 11133 | Hydrochlorothiazide with captopril 25mg with 50mg Tablet |
| 11197 | Innovace melt 5mg Wafer (Merck Sharp & Dohme Ltd) |
| 11351 | Co-zidocapt 25mg/50mg tablets |
| 11561 | Co-zidocapt 12.5mg/25mg tablets |
| 11567 | Ramipril 5mg with felodipine 5mg modified-release tablet |
| 11641 | Captopril 25mg with Hydrochlorothiazide 12.5mg tablets |
| 11937 | Ramipril 2.5mg/5ml oral suspension |
| 11965 | Ramipril 2.5mg with felodipine 2.5mg modified-release tablet |
| 11983 | Perindopril erbumine 4mg/5ml oral suspension |
| 11987 | Lisinopril 5mg/5ml oral solution |
| 12313 | Carace 20mg tablets (Bristol-Myers Squibb Pharmaceuticals Ltd) |
| 12411 | Cilazapril 500microgram tablets |
| 12412 | Cilazapril 2.5mg tablets |
| 12574 | Cilazapril 1mg tablets |
| 12815 | Tanatril 10mg tablets (Northumbria Pharma Ltd) |
| 12858 | Imidapril 10mg tablets |
| 13026 | Cilazapril 5mg tablets |
| 13589 | Staril 20mg tablets (Bristol-Myers Squibb Pharmaceuticals Ltd) |
| 13755 | Enalapril 10mg wafer |
| 14228 | Coversyl Plus tablets (Servier Laboratories Ltd) |
| 14387 | Carace 5mg tablets (Bristol-Myers Squibb Pharmaceuticals Ltd) |
| 14477 | Accupro 10mg tablets (Pfizer Ltd) |
| 14478 | Accupro 20mg tablets (Pfizer Ltd) |
| 14960 | Coversyl 8mg tablets (Servier Laboratories Ltd) |
| 15031 | Accuretic 10mg/12.5mg tablets (Pfizer Ltd) |
| 15085 | Innovace Titration pack (Merck Sharp & Dohme Ltd) |
| 15096 | Accupro 40mg tablets (Pfizer Ltd) |
| 15108 | Quinapril 10mg / Hydrochlorothiazide 12.5mg tablets |
| 15121 | Moexipril 7.5mg tablets |
| 15135 | Hydrochlorothiazide with captopril 12.5mg with 25mg Tablet |
| 15605 | Cilazapril 250micrograms tablets |
| 15958 | Captopril 2mg tablets |
| 16196 | Vascace 5mg tablets (Roche Products Ltd) |
| 16197 | Vascace 2.5mg tablets (Roche Products Ltd) |
| 16212 | Vascace 1mg tablets (Roche Products Ltd) |
| 16701 | Carace 10mg tablets (Bristol-Myers Squibb Pharmaceuticals Ltd) |
| 16708 | Enalapril titration pack |
| 16710 | Gopten 500microgram capsules (Abbott Laboratories Ltd) |
| 16924 | Imidapril 5mg tablets |
| 17006 | Triapin 5mg/5mg modified-release tablets (Sanofi) |
| 17120 | Moexipril 15mg tablets |
| 17474 | Felodipine 5mg modified-release / Ramipril 5mg tablets |
| 17624 | Captopril 5mg/5ml oral suspension |
| 17633 | Captopril 3mg/5ml oral solution |
| 17655 | Carace 10 Tablet (Bristol-Myers Squibb Pharmaceuticals Ltd) |
| 18219 | Imidapril 20mg tablets |
| 18223 | Trandolapril with verapamil 2mg + 180mg Modified-release capsule |
| 18263 | Acezide 25mg/50mg tablets (Bristol-Myers Squibb Pharmaceuticals Ltd) |
| 18269 | Acepril 12.5mg tablets (Bristol-Myers Squibb Pharmaceuticals Ltd) |
| 18325 | Acepril 50mg tablets (Bristol-Myers Squibb Pharmaceuticals Ltd) |
| 19198 | Lisinopril 20mg tablets (Teva UK Ltd) |
| 19204 | Lisinopril 5mg tablets (Teva UK Ltd) |
| 19208 | Enalapril 10mg tablets (Actavis UK Ltd) |
| 19223 | Lisinopril 10mg tablets (Teva UK Ltd) |
| 20188 | Enalapril 2.5mg wafer |
| 20579 | Tarka modified-release capsules (Abbott Laboratories Ltd) |
| 20849 | Tensopril 12.5mg tablets (Teva UK Ltd) |
| 20975 | Lisinopril 7.5mg/5ml oral suspension |
| 21053 | Vascace 500microgram tablets (Roche Products Ltd) |
| 21162 | Felodipine 2.5mg modified-release / Ramipril 2.5mg tablets |
| 21231 | Caralpha 20mg/12.5mg tablets (Actavis UK Ltd) |
| 21943 | Kaplon 12.5mg tablets (Teva UK Ltd) |
| 22439 | Ednyt 20mg Tablet (Dominion Pharma) |
| 22708 | Enalapril 5mg wafer |
| 23252 | Pralenal 10 tablets (Opus Pharmaceuticals Ltd) |
| 23478 | Tensopril 50mg tablets (Teva UK Ltd) |
| 23642 | Vascace 0.25mg Tablet (Roche Products Ltd) |
| 24041 | Enalapril 20mg wafer |
| 24482 | Captomex 50mg tablets (Actavis UK Ltd) |
| 25998 | Captomex 12.5mg tablets (Actavis UK Ltd) |
| 26995 | Kaplon 25mg tablets (Teva UK Ltd) |
| 27871 | Innovace melt 10mg Wafer (Merck Sharp & Dohme Ltd) |
| 28127 | Enalapril 2.5mg tablets (Teva UK Ltd) |
| 28438 | Triapin 2.5mg/2.5mg modified-release tablets (Sanofi) |
| 28486 | Captopril 6.25mg/5ml oral suspension |
| 28586 | Lopace 5mg capsules (Discovery Pharmaceuticals) |
| 28724 | Perdix 7.5mg tablets (UCB Pharma Ltd) |
| 28725 | Perdix 15mg tablets (UCB Pharma Ltd) |
| 28820 | Captomex 25mg tablets (Actavis UK Ltd) |
| 28902 | Odrik 2mg capsules (Aventis Pharma) |
| 29130 | Gopten 4mg capsules (Abbott Laboratories Ltd) |
| 29530 | Innovace melt 2.5mg Wafer (Merck Sharp & Dohme Ltd) |
| 29627 | Lopace 2.5mg capsules (Discovery Pharmaceuticals) |
| 30039 | Tensopril 25mg tablets (Teva UK Ltd) |
| 30921 | Lisinopril 2.5mg tablets (Teva UK Ltd) |
| 31307 | Odrik 500microgram capsules (Aventis Pharma) |
| 31587 | Innovace melt 20mg Wafer (Merck Sharp & Dohme Ltd) |
| 31716 | Enalapril 20mg tablets (Accord Healthcare Ltd) |
| 31810 | Odrik 1mg capsules (Aventis Pharma) |
| 32048 | Kaplon 50mg tablets (Teva UK Ltd) |
| 32166 | Capto-co 25mg+50mg Tablet (IVAX Pharmaceuticals UK Ltd) |
| 32241 | Enalapril 10mg tablets (A A H Pharmaceuticals Ltd) |
| 32514 | Ecopace 25mg tablets (Advanz Pharma) |
| 32560 | Tanatril 20mg tablets (Northumbria Pharma Ltd) |
| 32597 | Lisinopril 10mg tablets (Sandoz Ltd) |
| 32857 | Ramipril 1.25mg capsules (Teva UK Ltd) |
| 32934 | Lopace 10mg capsules (Discovery Pharmaceuticals) |
| 33057 | Ednyt 5mg Tablet (Dominion Pharma) |
| 33078 | Enalapril 20mg tablets (A A H Pharmaceuticals Ltd) |
| 33095 | Perindopril erbumine 4mg tablets (A A H Pharmaceuticals Ltd) |
| 33336 | Captopril 5mg/5ml Oral suspension (Eldon Laboratories) |
| 33353 | Lisinopril 20mg / Hydrochlorothiazide 12.5mg tablets (Teva UK Ltd) |
| 33646 | Captopril 12.5mg Tablet (Generics (UK) Ltd) |
| 33811 | Ramipril 2.5mg capsules (Ranbaxy (UK) Ltd) |
| 33894 | Ramipril 10mg capsules (Teva UK Ltd) |
| 33977 | Lisinopril 10mg tablets (Viatris UK Healthcare Ltd) |
| 34357 | Ramipril 10mg capsules (Genus Pharmaceuticals Ltd) |
| 34382 | Ramipril 5mg capsules (Zentiva Pharma UK Ltd) |
| 34390 | Ramipril 5mg capsules (Genus Pharmaceuticals Ltd) |
| 34400 | Enalapril 5mg Tablet (Dowelhurst Ltd) |
| 34412 | Ramipril 5mg capsules (Teva UK Ltd) |
| 34429 | Ramipril 5mg capsules (Viatris UK Healthcare Ltd) |
| 34431 | Ramipril 2.5mg capsules (Zentiva Pharma UK Ltd) |
| 34432 | Ramipril 2.5mg capsules (Genus Pharmaceuticals Ltd) |
| 34453 | Enalapril 20mg tablets (Viatris UK Healthcare Ltd) |
| 34471 | Lisinopril 5mg tablets (Viatris UK Healthcare Ltd) |
| 34490 | Ramipril 2.5mg capsules (Teva UK Ltd) |
| 34505 | Ramipril 2.5mg capsules (Sandoz Ltd) |
| 34528 | Ramipril 2.5mg capsules (A A H Pharmaceuticals Ltd) |
| 34539 | Ramipril 5mg capsules (Sandoz Ltd) |
| 34540 | Ramipril 5mg capsules (A A H Pharmaceuticals Ltd) |
| 34544 | Captopril 12.5mg Tablet (IVAX Pharmaceuticals UK Ltd) |
| 34562 | Captopril 25mg Tablet (IVAX Pharmaceuticals UK Ltd) |
| 34567 | Ramipril 2.5mg capsules (Viatris UK Healthcare Ltd) |
| 34583 | Ramipril 10mg Capsule (Dexcel-Pharma Ltd) |
| 34589 | Ramipril 5mg Capsule (Dexcel-Pharma Ltd) |
| 34651 | Ramipril 10mg capsules (Viatris UK Healthcare Ltd) |
| 34652 | Ramipril 5mg Capsule (Sovereign Medical Ltd) |
| 34657 | Ramipril 10mg capsules (Zentiva Pharma UK Ltd) |
| 34696 | Lisinopril 20mg tablets (Viatris UK Healthcare Ltd) |
| 34698 | Ramipril 1.25mg capsules (Zentiva Pharma UK Ltd) |
| 34710 | Ramipril 10mg capsules (Sandoz Ltd) |
| 34712 | Enalapril 20mg tablets (Kent Pharma (UK) Ltd) |
| 34719 | Captopril 50mg Tablet (Generics (UK) Ltd) |
| 34732 | Ramipril 2.5mg Capsule (Dexcel-Pharma Ltd) |
| 34768 | Enalapril 20mg tablets (IVAX Pharmaceuticals UK Ltd) |
| 34798 | Enalapril 20mg tablets (Sandoz Ltd) |
| 34799 | Lisinopril 20mg tablets (Zentiva Pharma UK Ltd) |
| 34877 | Ramipril 10mg Capsule (Sovereign Medical Ltd) |
| 34893 | Ramipril 10mg Capsule (IVAX Pharmaceuticals UK Ltd) |
| 34936 | Captopril 25mg Tablet (Lagap) |
| 34937 | Captopril 50mg Tablet (IVAX Pharmaceuticals UK Ltd) |
| 34943 | Ramipril 10mg capsules (A A H Pharmaceuticals Ltd) |
| 34952 | Enalapril 10mg tablets (Viatris UK Healthcare Ltd) |
| 34953 | Enalapril 20mg tablets (Zentiva Pharma UK Ltd) |
| 35007 | Ramipril 10mg/5ml oral suspension |
| 35302 | Captopril 12.5mg/5ml oral suspension |
| 35731 | Perindopril erbumine 8mg tablets (A A H Pharmaceuticals Ltd) |
| 35794 | Enalapril 5mg tablets (A A H Pharmaceuticals Ltd) |
| 36742 | Captopril 2mg/5ml oral suspension |
| 36753 | Ednyt 10mg Tablet (Dominion Pharma) |
| 37080 | Enalapril 5mg/5ml oral solution |
| 37087 | Enalapril 5mg/5ml oral suspension |
| 37655 | Captopril 25mg tablets (Teva UK Ltd) |
| 37710 | Lisinopril 10mg / Hydrochlorothiazide 12.5mg tablets (Teva UK Ltd) |
| 37778 | Lisinopril 5mg/5ml oral suspension |
| 37908 | Coversyl Arginine Plus 5mg/1.25mg tablets (Servier Laboratories Ltd) |
| 37930 | Perindopril arginine 5mg tablets |
| 37964 | Perindopril arginine 2.5mg tablets |
| 37965 | Coversyl Arginine 5mg tablets (Servier Laboratories Ltd) |
| 37971 | Perindopril arginine 10mg tablets |
| 37978 | Perindopril arginine 5mg / Indapamide 1.25mg tablets |
| 38026 | Coversyl Arginine 10mg tablets (Servier Laboratories Ltd) |
| 38034 | Coversyl Arginine 2.5mg tablets (Servier Laboratories Ltd) |
| 38285 | Perindopril erbumine 4mg tablets (Teva UK Ltd) |
| 38308 | Ramipril 2.5/5mg/10mg tablet |
| 38510 | Perindopril erbumine 4mg tablets (Apotex UK Ltd) |
| 38854 | Quinapril 20mg/5ml oral solution |
| 38899 | Quinil 10mg tablets (Tillomed Laboratories Ltd) |
| 38995 | Zestoretic 20 tablets (Atnahs Pharma UK Ltd) |
| 39137 | Zestoretic 10 tablets (Atnahs Pharma UK Ltd) |
| 39147 | Carace 20 Plus tablets (Organon Pharma (UK) Ltd) |
| 39227 | Capozide LS 12.5mg/25mg tablets (Bristol-Myers Squibb Pharmaceuticals Ltd) |
| 39242 | Carace 10 Plus tablets (Organon Pharma (UK) Ltd) |
| 39355 | Tritace 10mg Tablet (Sterwin Medicines) |
| 39421 | Tritace titration pack tablets (Sanofi) |
| 39512 | Captopril 25mg/5ml oral suspension |
| 40355 | Quinil 5mg tablets (Tillomed Laboratories Ltd) |
| 40384 | Ramipril 10mg tablets (A A H Pharmaceuticals Ltd) |
| 41417 | Enalapril 2.5mg tablets (A A H Pharmaceuticals Ltd) |
| 41522 | Lisopress 20mg tablets (Teva UK Ltd) |
| 41532 | Lisopress 5mg tablets (Teva UK Ltd) |
| 41538 | Lisopress 2.5mg tablets (Teva UK Ltd) |
| 41573 | Lisopress 10mg tablets (Teva UK Ltd) |
| 41617 | Captopril 25mg tablets (Actavis UK Ltd) |
| 41633 | Captopril 12.5mg tablets (Actavis UK Ltd) |
| 41694 | Enalapril 2.5mg tablets (IVAX Pharmaceuticals UK Ltd) |
| 41743 | Captopril 50mg tablets (Teva UK Ltd) |
| 41746 | Enalapril 10mg tablets (Sandoz Ltd) |
| 42081 | Tritace 1.25mg Tablet (Sterwin Medicines) |
| 42285 | Quinil 40mg tablets (Tillomed Laboratories Ltd) |
| 42723 | Pralenal 5 tablets (Opus Pharmaceuticals Ltd) |
| 42894 | Enalapril 10mg tablets (Teva UK Ltd) |
| 42901 | Enalapril 5mg tablets (Teva UK Ltd) |
| 42902 | Enalapril 20mg tablets (Teva UK Ltd) |
| 42908 | Enalapril 5mg tablets (IVAX Pharmaceuticals UK Ltd) |
| 43012 | Perindopril erbumine oral solution |
| 43411 | Enalapril 5mg tablets (Sandoz Ltd) |
| 43412 | Lisinopril 2.5mg tablets (A A H Pharmaceuticals Ltd) |
| 43413 | Lisinopril 20mg tablets (A A H Pharmaceuticals Ltd) |
| 43416 | Lisinopril 10mg tablets (A A H Pharmaceuticals Ltd) |
| 43418 | Lisinopril 5mg tablets (A A H Pharmaceuticals Ltd) |
| 43432 | Captopril 6.25mg tablets |
| 43507 | Captopril 25mg Tablet (Generics (UK) Ltd) |
| 43563 | Enalapril 2.5mg tablets (Zentiva Pharma UK Ltd) |
| 43566 | Lisinopril 2.5mg tablets (Sandoz Ltd) |
| 43649 | Captopril 25mg tablets (A A H Pharmaceuticals Ltd) |
| 43813 | Perindopril erbumine 2mg tablets (Actavis UK Ltd) |
| 44527 | Captopril 5mg/ml oral solution sugar free |
| 44657 | Ednyt 2.5mg Tablet (Dominion Pharma) |
| 45217 | Enalapril 5mg tablets (Kent Pharma (UK) Ltd) |
| 45228 | Captopril capsules |
| 45264 | Ramipril 1.25mg capsules (Accord Healthcare Ltd) |
| 45300 | Lisinopril 10mg tablets (Actavis UK Ltd) |
| 45319 | Perindopril erbumine 2mg tablets (A A H Pharmaceuticals Ltd) |
| 45324 | Lisinopril 20mg tablets (Actavis UK Ltd) |
| 45337 | Lisinopril 5mg tablets (Actavis UK Ltd) |
| 45340 | Ramipril 10mg Capsule (Actavis UK Ltd) |
| 45554 | Ramipril 5mg/5ml oral solution |
| 45816 | Lisinopril 5mg tablets (Almus Pharmaceuticals Ltd) |
| 45938 | Perindopril erbumine 8mg tablets (Teva UK Ltd) |
| 46365 | Quinil 20mg tablets (Tillomed Laboratories Ltd) |
| 46851 | Captopril 5mg/5ml oral solution |
| 46890 | Ramipril 5mg/5ml oral suspension |
| 46951 | Captopril 12.5mg tablets (A A H Pharmaceuticals Ltd) |
| 46957 | Captopril 12.5mg tablets (Tillomed Laboratories Ltd) |
| 46974 | Enalapril 5mg tablets (Viatris UK Healthcare Ltd) |
| 46975 | Lisinopril 5mg tablets (Sandoz Ltd) |
| 46979 | Lisinopril 20mg tablets (Sandoz Ltd) |
| 47021 | Ramipril 2.5mg/5ml oral solution sugar free |
| 47159 | Lisinopril 10mg tablets (Almus Pharmaceuticals Ltd) |
| 47998 | Ramipril 2.5mg capsules (Accord Healthcare Ltd) |
| 48008 | Ramipril 5mg capsules (Accord Healthcare Ltd) |
| 48049 | Perindopril erbumine 2mg tablets (Viatris UK Healthcare Ltd) |
| 48053 | Ramipril 2.5mg capsules (Almus Pharmaceuticals Ltd) |
| 48098 | Perindopril arginine 4mg with Indapamide 1.25mg tablet |
| 48180 | Perindopril erbumine 4mg tablets (Sandoz Ltd) |
| 48214 | Perindopril erbumine 4mg tablets (Actavis UK Ltd) |
| 49164 | Ramipril 10mg capsules (Accord Healthcare Ltd) |
| 49491 | Perindopril erbumine 2mg tablets (Consilient Health Ltd) |
| 50334 | Enalapril 4mg/5ml oral suspension |
| 50347 | Coversyl Arginine 5mg tablets (Waymade Healthcare Plc) |
| 50402 | Perindopril 2mg Tablet (Servier Laboratories Ltd) |
| 50509 | Ramipril 10mg/5ml oral solution |
| 50607 | Perindopril arginine 2mg with Indapamide 625 micrograms tablet |
| 50780 | Enalapril 2mg/5ml oral solution |
| 50863 | Enalapril 5mg/5ml oral solution (Drug Tariff Special Order) |
| 51258 | Coversyl Arginine Plus 5mg/1.25mg tablets (DE Pharmaceuticals) |
| 51433 | Lisinopril 20mg tablets (Tillomed Laboratories Ltd) |
| 51701 | Ramipril 5mg capsules (Bristol Laboratories Ltd) |
| 51714 | Ramipril 2.5mg capsules (Alliance Healthcare (Distribution) Ltd) |
| 51807 | Coversyl Arginine 5mg tablets (DE Pharmaceuticals) |
| 52010 | Enalapril 10mg tablets (Alliance Healthcare (Distribution) Ltd) |
| 52088 | Lisinopril 5mg tablets (Phoenix Healthcare Distribution Ltd) |
| 52197 | Ramipril 5mg capsules (Sigma Pharmaceuticals Plc) |
| 52293 | Captopril 2mg capsules |
| 52399 | Ramipril 1.25mg capsules (Kent Pharma (UK) Ltd) |
| 52407 | Ramipril 10mg capsules (Kent Pharma (UK) Ltd) |
| 52499 | Captopril 25mg/5ml oral solution |
| 52882 | Enalapril 5mg/5ml oral suspension sugar free |
| 53058 | Perindopril erbumine 8mg tablets (Sandoz Ltd) |
| 53271 | Lisinopril 10mg tablets (Alliance Healthcare (Distribution) Ltd) |
| 53551 | Lisinopril 20mg tablets (Phoenix Healthcare Distribution Ltd) |
| 53612 | Ramipril 10mg tablets (Alliance Healthcare (Distribution) Ltd) |
| 53621 | Ramipril 2.5mg capsules (Bristol Laboratories Ltd) |
| 53719 | Enalapril 20mg tablets (Alliance Healthcare (Distribution) Ltd) |
| 53820 | Lisinopril 5mg tablets (Arrow Generics Ltd) |
| 53915 | Enalapril 5mg tablets (Dexcel-Pharma Ltd) |
| 54037 | Lisinopril 10mg tablets (Relonchem Ltd) |
| 54201 | Lisinopril 20mg / Hydrochlorothiazide 12.5mg tablets (Almus Pharmaceuticals Ltd) |
| 54283 | Lisinopril 5mg/5ml oral suspension (Special Order) |
| 54288 | Lisinopril 10mg tablets (Arrow Generics Ltd) |
| 54298 | Ramipril 2.5mg capsules (Arrow Generics Ltd) |
| 54345 | Trandolapril 4mg capsules (Arrow Generics Ltd) |
| 54512 | Lisinopril Oral solution |
| 54544 | Captopril 25mg/5ml oral suspension |
| 54620 | Ramipril 2.5mg capsules (Sigma Pharmaceuticals Plc) |
| 54733 | Perindopril erbumine 8mg tablets (Consilient Health Ltd) |
| 54899 | Perindopril erbumine 2mg tablets (Teva UK Ltd) |
| 54928 | Lisinopril 10mg tablets (Bristol Laboratories Ltd) |
| 54941 | Ramipril 5mg capsules (Alliance Healthcare (Distribution) Ltd) |
| 54942 | Perindopril erbumine 8mg tablets (Viatris UK Healthcare Ltd) |
| 54986 | Perindopril erbumine 8mg/5ml oral suspension |
| 55002 | Lisinopril 20mg tablets (Accord Healthcare Ltd) |
| 55299 | Ramipril 1.25mg capsules (A A H Pharmaceuticals Ltd) |
| 55399 | Lisinopril 20mg / Hydrochlorothiazide 12.5mg tablets (A A H Pharmaceuticals Ltd) |
| 55456 | Lisinopril 5mg tablets (Alliance Healthcare (Distribution) Ltd) |
| 55588 | Lisinopril 20mg tablets (Sigma Pharmaceuticals Plc) |
| 55639 | Lisinopril 10mg tablets (Accord Healthcare Ltd) |
| 55798 | Ramipril 5mg capsules (Waymade Healthcare Plc) |
| 55896 | Lisinopril 2.5mg tablets (Actavis UK Ltd) |
| 55903 | Enalapril 10mg tablets (Dexcel-Pharma Ltd) |
| 56013 | Ramipril 2.5mg capsules (Waymade Healthcare Plc) |
| 56038 | Ramipril 10mg tablets (Pfizer Ltd) |
| 56079 | Perindopril tosilate 10mg tablets |
| 56129 | Ramipril 5mg capsules (Kent Pharma (UK) Ltd) |
| 56148 | Ramipril 1.25mg tablets (Kent Pharma (UK) Ltd) |
| 56157 | Perindopril tosilate 5mg / Indapamide 1.25mg tablets |
| 56162 | Perindopril erbumine 4mg tablets (Consilient Health Ltd) |
| 56169 | Ramipril 10mg capsules (Arrow Generics Ltd) |
| 56244 | Lisinopril 20mg / Hydrochlorothiazide 12.5mg tablets (Tillomed Laboratories Ltd) |
| 56279 | Lisinopril 2.5mg/5ml oral solution |
| 56356 | Ramipril 10mg capsules (Alliance Healthcare (Distribution) Ltd) |
| 56472 | Perindopril erbumine 4mg tablets (Kent Pharma (UK) Ltd) |
| 56473 | Perindopril erbumine 2mg tablets (Sigma Pharmaceuticals Plc) |
| 56505 | Zestril 5mg tablets (Lexon (UK) Ltd) |
| 56506 | Coversyl 2mg tablets (Dowelhurst Ltd) |
| 56508 | Coversyl 4mg tablets (Dowelhurst Ltd) |
| 56509 | Capoten 12.5mg tablets (Dowelhurst Ltd) |
| 56510 | Zestril 20mg tablets (Sigma Pharmaceuticals Plc) |
| 56516 | Perindopril erbumine 2mg tablets (Sandoz Ltd) |
| 56704 | Ramipril 1.25mg capsules (Alliance Healthcare (Distribution) Ltd) |
| 56763 | Ramipril 10mg capsules (Phoenix Healthcare Distribution Ltd) |
| 56850 | Ecopace 12.5mg tablets (Advanz Pharma) |
| 56855 | Ramipril 10mg capsules (Sigma Pharmaceuticals Plc) |
| 57048 | Lisinopril 10mg tablets (Zentiva Pharma UK Ltd) |
| 57073 | Ramipril 1.25mg capsules (Waymade Healthcare Plc) |
| 57235 | Ramipril 1.25mg tablets (Sandoz Ltd) |
| 57333 | Perindopril tosilate 5mg tablets |
| 57346 | Ramipril 10mg capsules (Waymade Healthcare Plc) |
| 57378 | Enalapril 2mg/5ml oral suspension |
| 57539 | Zestoretic 10 tablets (Sigma Pharmaceuticals Plc) |
| 57588 | Zestril 2.5mg tablets (Mawdsley-Brooks & Company Ltd) |
| 57658 | Ramipril 1.25mg tablets (A A H Pharmaceuticals Ltd) |
| 57701 | Perindopril erbumine 8mg tablets (Actavis UK Ltd) |
| 57801 | Perindopril erbumine 4mg tablets (Glenmark Pharmaceuticals Europe Ltd) |
| 57864 | Ramipril 5mg tablets (Sigma Pharmaceuticals Plc) |
| 57882 | Enalapril 2.5mg/5ml oral suspension |
| 57944 | Perindopril tosilate 2.5mg tablets |
| 58195 | Captopril 12.5mg/5ml oral solution |
| 58258 | Lisinopril 2.5mg/5ml oral suspension |
| 58294 | Lisinopril 5mg tablets (Accord Healthcare Ltd) |
| 58451 | Lisinopril 2.5mg tablets (Almus Pharmaceuticals Ltd) |
| 58461 | Lisinopril 2.5mg tablets (Kent Pharma (UK) Ltd) |
| 58682 | Lisinopril 2.5mg tablets (Viatris UK Healthcare Ltd) |
| 58751 | Enalapril 1.25mg/5ml oral suspension |
| 58843 | Perindopril erbumine 2mg tablets (Kent Pharma (UK) Ltd) |
| 58863 | Lisinopril 10mg tablets (Phoenix Healthcare Distribution Ltd) |
| 58871 | Lisinopril 10mg tablets (Waymade Healthcare Plc) |
| 58874 | Perindopril erbumine 2mg tablets (Somex Pharma) |
| 59109 | Lisinopril 5mg tablets (Tillomed Laboratories Ltd) |
| 59111 | Lisinopril 20mg tablets (Alliance Healthcare (Distribution) Ltd) |
| 59557 | Ramipril 2.5mg capsules (Kent Pharma (UK) Ltd) |
| 59603 | Ramipril 2.5mg capsules (Phoenix Healthcare Distribution Ltd) |
| 59699 | Captopril 5mg/5ml oral solution sugar free |
| 59770 | Perindopril erbumine 4mg tablets (Milpharm Ltd) |
| 59788 | Ramipril 10mg capsules (Bristol Laboratories Ltd) |
| 59790 | Perindopril erbumine 8mg tablets (Accord Healthcare Ltd) |
| 59915 | Captopril 25mg/5ml oral solution sugar free |
| 59972 | Perindopril erbumine 2mg tablets (Alliance Healthcare (Distribution) Ltd) |
| 59996 | Enalapril 20mg tablets (Milpharm Ltd) |
| 60010 | Lisinopril 10mg tablets (Kent Pharma (UK) Ltd) |
| 60065 | Perindopril erbumine 4mg tablets (Sigma Pharmaceuticals Plc) |
| 60067 | Perindopril erbumine 4mg / Amlodipine 5mg tablets |
| 60097 | Lisinopril 2.5mg tablets (Zentiva Pharma UK Ltd) |
| 60143 | Enalapril 5mg tablets (Medreich Plc) |
| 60232 | Lisinopril 5mg tablets (Zentiva Pharma UK Ltd) |
| 60309 | Lisinopril 5mg tablets (Relonchem Ltd) |
| 60349 | Noyada 25mg/5ml oral solution (Martindale Pharmaceuticals Ltd) |
| 60684 | Perindopril erbumine 4mg / Amlodipine 10mg tablets |
| 60730 | Ramipril 5mg capsules (Phoenix Healthcare Distribution Ltd) |
| 60744 | Perindopril erbumine 8mg / Amlodipine 5mg tablets |
| 60757 | Trandolapril 500microgram capsules (Teva UK Ltd) |
| 60823 | Noyada 5mg/5ml oral solution (Martindale Pharmaceuticals Ltd) |
| 61067 | Ramipril 5mg capsules (Almus Pharmaceuticals Ltd) |
| 61117 | Perindopril erbumine 4mg/5ml oral solution |
| 61133 | Enalapril 10mg tablets (Phoenix Healthcare Distribution Ltd) |
| 61262 | Lisinopril 20mg tablets (Bristol Laboratories Ltd) |
| 61270 | Perindopril erbumine 4mg tablets (Accord Healthcare Ltd) |
| 61292 | Quinapril 40mg tablets (Viatris UK Healthcare Ltd) |
| 61339 | Ramipril 10mg capsules (Almus Pharmaceuticals Ltd) |
| 61499 | Ramipril 2.5mg tablets (Accord Healthcare Ltd) |
| 61693 | Perindopril erbumine 8mg tablets (Milpharm Ltd) |
| 61694 | Ramipril 5mg tablets (Zentiva Pharma UK Ltd) |
| 61985 | Ramipril 1.25mg tablets (Teva UK Ltd) |
| 62036 | Ramipril 5mg tablets (Waymade Healthcare Plc) |
| 62039 | Ramipril 1.25mg tablets (Zentiva Pharma UK Ltd) |
| 62564 | Lisinopril 10mg/5ml oral solution |
| 62860 | Enalapril 5mg tablets (DE Pharmaceuticals) |
| 62918 | Ramipril 2.5mg/5ml oral solution |
| 62958 | Ramipril 5mg tablets (Teva UK Ltd) |
| 63010 | Ramipril 10mg tablets (Phoenix Healthcare Distribution Ltd) |
| 63030 | Lisinopril 10mg tablets (DE Pharmaceuticals) |
| 63149 | Perindopril erbumine 8mg / Amlodipine 10mg tablets |
| 63322 | Enalapril 10mg tablets (Almus Pharmaceuticals Ltd) |
| 63442 | Ramipril 2.5mg tablets (Teva UK Ltd) |
| 63559 | Lisinopril 20mg tablets (Kent Pharma (UK) Ltd) |
| 63594 | Generic Tritace titration pack tablets |
| 63824 | Lisinopril 10mg/5ml oral suspension |
| 64055 | Ramipril 2.5mg/5ml oral solution sugar free (Waymade Healthcare Plc) |
| 64062 | Enalapril 1mg/5ml oral suspension |
| 64602 | Perindopril erbumine 2mg tablets (Waymade Healthcare Plc) |
| 64739 | Captopril 25mg/5ml oral solution (Special Order) |
| 64877 | Enalapril 2.5mg tablets (Dexcel-Pharma Ltd) |
| 64902 | Lisinopril 5mg/5ml oral solution sugar free |
| 65102 | Lisinopril 10mg tablets (Sigma Pharmaceuticals Plc) |
| 65273 | Perindopril erbumine 4mg tablets (Viatris UK Healthcare Ltd) |
| 65389 | Gopten 500microgram capsules (Waymade Healthcare Plc) |
| 65416 | Lisinopril 5mg tablets (Lupin Healthcare (UK) Ltd) |
| 65443 | Ramipril 1.25mg Tablet (Sovereign Medical Ltd) |
| 65536 | Lisinopril 2.5mg tablets (Alliance Healthcare (Distribution) Ltd) |
| 65570 | Trandolapril 4mg capsules (Teva UK Ltd) |
| 65599 | Ramipril 5mg tablets (A A H Pharmaceuticals Ltd) |
| 65749 | Ramipril 5mg capsules (Ennogen Pharma Ltd) |
| 65936 | Ramipril 5mg capsules (DE Pharmaceuticals) |
| 65983 | Lisinopril 2.5mg tablets (Lupin Healthcare (UK) Ltd) |
| 65985 | Lisinopril 2.5mg tablets (DE Pharmaceuticals) |
| 66060 | Perindopril erbumine 2mg tablets (DE Pharmaceuticals) |
| 66162 | Ramipril 10mg tablets (Teva UK Ltd) |
| 66329 | Ramipril oral solution |
| 66558 | Lisinopril 5mg tablets (DE Pharmaceuticals) |
| 66597 | Captopril 10mg/5ml oral suspension |
| 66622 | Lisinopril 20mg tablets (DE Pharmaceuticals) |
| 66623 | Trandolapril 2mg capsules (A A H Pharmaceuticals Ltd) |
| 66669 | Ramipril 10mg capsules (DE Pharmaceuticals) |
| 66772 | Lisinopril 2.5mg tablets (Waymade Healthcare Plc) |
| 66895 | Enalapril 10mg/5ml oral suspension |
| 67075 | Lisinopril 2.5mg tablets (Mawdsley-Brooks & Company Ltd) |
| 67194 | Lisinopril 2.5mg tablets (Bristol Laboratories Ltd) |
| 67269 | Coversyl 2mg tablets (Waymade Healthcare Plc) |
| 67307 | Staril 20mg tablets (Dowelhurst Ltd) |
| 67719 | Ramipril 5mg capsules (Mawdsley-Brooks & Company Ltd) |
| 67741 | Ramipril 1.25mg capsules (Almus Pharmaceuticals Ltd) |
| 67767 | Lisinopril 10mg / Hydrochlorothiazide 12.5mg tablets (Almus Pharmaceuticals Ltd) |
| 67789 | Perindopril erbumine 2mg tablets (Accord Healthcare Ltd) |
| 67795 | Lisinopril 20mg tablets (Almus Pharmaceuticals Ltd) |
| 68021 | Perindopril erbumine 4mg tablets (Alliance Healthcare (Distribution) Ltd) |
| 68094 | Lisinopril 5mg tablets (Sigma Pharmaceuticals Plc) |
| 68192 | Ramipril 5mg capsules (Brown & Burk UK Ltd) |
| 68247 | Lisinopril 5mg tablets (Bristol Laboratories Ltd) |
| 68372 | Ramipril 5mg tablets (Accord Healthcare Ltd) |
| 68381 | Perindopril erbumine 4mg tablets (Mawdsley-Brooks & Company Ltd) |
| 68480 | Ramipril 10mg capsules (Mawdsley-Brooks & Company Ltd) |
| 68496 | Enalapril 10mg tablets (Kent Pharma (UK) Ltd) |
| 68759 | Perindopril erbumine 2mg tablets (Milpharm Ltd) |
| 69016 | Perindopril erbumine 8mg tablets (DE Pharmaceuticals) |
| 69074 | Lisinopril 20mg tablets (Relonchem Ltd) |
| 69192 | Captopril oral solution |
| 69269 | Lisinopril 20mg tablets (Waymade Healthcare Plc) |
| 69288 | Ramipril 10mg capsules (Brown & Burk UK Ltd) |
| 69599 | Captopril 500micrograms/5ml oral suspension |
| 69600 | Captopril 1mg/5ml oral suspension |
| 70072 | Ramipril 1.25mg capsules (Mawdsley-Brooks & Company Ltd) |
| 70667 | Lisinopril 5mg tablets (Mawdsley-Brooks & Company Ltd) |
| 70709 | Ramipril 10mg tablets (Accord Healthcare Ltd) |
| 70916 | Perindopril erbumine 8mg tablets (Glenmark Pharmaceuticals Europe Ltd) |
| 70917 | Perindopril erbumine 2mg tablets (Glenmark Pharmaceuticals Europe Ltd) |
| 70994 | Captopril 12.5mg tablets (Sandoz Ltd) |
| 71004 | Perindopril erbumine 8mg tablets (Accord Healthcare Ltd) |
| 71025 | Ramipril 1.25mg tablets (APC Pharmaceuticals & Chemicals (Europe) Ltd) |
| 71040 | Ramipril 5mg tablets (Pfizer Ltd) |
| 71068 | Ramipril 2.5mg tablets (APC Pharmaceuticals & Chemicals (Europe) Ltd) |
| 71115 | Zestoretic 10 tablets (Waymade Healthcare Plc) |
| 71277 | Captopril 7.5mg/5ml oral suspension |
| 71491 | Ramipril 2.5mg capsules (Ennogen Pharma Ltd) |
| 71562 | Lisinopril 10mg Tablet (Niche Generics Ltd) |
| 71668 | Enalapril 20mg tablets (Almus Pharmaceuticals Ltd) |
| 71737 | Enalapril 2.5mg tablets (Almus Pharmaceuticals Ltd) |
| 72017 | Enalapril 1.25mg/5ml oral solution |
| 72038 | Lisinopril 2.5mg tablets (Relonchem Ltd) |
| 72295 | Perindopril 2mg Tablet (Neo Laboratories Ltd) |
| 72336 | Lisinopril 5mg tablets (Waymade Healthcare Plc) |
| 72341 | Ramipril 2.5mg capsules (Wockhardt UK Ltd) |
| 72842 | Ramipril 10mg capsules (Wockhardt UK Ltd) |
| 72941 | Perindopril erbumine 1mg/5ml oral suspension |
| 73389 | Enalapril 2.5mg tablets (DE Pharmaceuticals) |
| 73459 | Ramipril 2.5mg/5ml oral solution sugar free (A A H Pharmaceuticals Ltd) |
| 73484 | Tritace 5mg Tablet (Sterwin Medicines) |
| 73617 | Enalapril 1.5mg/5ml oral suspension |
| 73659 | Captopril 50mg tablets (Kent Pharma (UK) Ltd) |
| 73672 | Lisinopril 10mg tablets (Milpharm Ltd) |
| 73716 | Lisinopril 2.5mg tablets (Crescent Pharma Ltd) |
| 74008 | Zestoretic 20 tablets (Waymade Healthcare Plc) |
| 74040 | Zestoretic 20 tablets (Lexon (UK) Ltd) |
| 74066 | Ramipril 10mg tablets (Tillomed Laboratories Ltd) |
| 74155 | Lisinopril 5mg tablets (Milpharm Ltd) |
| 74209 | Trandolapril 1mg capsules (Accord Healthcare Ltd) |
| 74237 | Enalapril 25mg/5ml oral solution |
| 74417 | Captopril 20mg/5ml oral suspension |
| 74618 | Ramipril 2.5mg capsules (Mawdsley-Brooks & Company Ltd) |
| 74627 | Captopril 25mg Tablet (C P Pharmaceuticals Ltd) |
| 74632 | Ramipril 5mg Tablet (Sovereign Medical Ltd) |
| 74874 | Zestoretic 20 tablets (DE Pharmaceuticals) |
| 75021 | Perindopril erbumine 4mg tablets (Ranbaxy (UK) Ltd) |
| 75024 | Perindopril erbumine 4mg tablets (Phoenix Healthcare Distribution Ltd) |
| 75410 | Ramipril 2.5mg capsules (Brown & Burk UK Ltd) |
| 75847 | Perindopril erbumine 8mg/5ml oral solution |
| 76100 | Lisinopril 5mg tablets (Kent Pharma (UK) Ltd) |
| 76128 | Lisinopril 20mg tablets (Lupin Healthcare (UK) Ltd) |
| 76433 | Captopril 1.5mg capsules |
| 76486 | Enalapril 2.5mg tablets (Viatris UK Healthcare Ltd) |
| 76548 | Ramipril 1.25mg capsules (Phoenix Healthcare Distribution Ltd) |
| 76567 | Ramipril 1.25mg tablets (Sigma Pharmaceuticals Plc) |
| 76619 | Enalapril 20mg tablets (Dexcel-Pharma Ltd) |
| 76784 | Ramipril 5mg capsules (Wockhardt UK Ltd) |
| 76920 | Enalapril 20mg / Hydrochlorothiazide 12.5mg tablets (Tillomed Laboratories Ltd) |
| 77046 | Captopril 3mg oral powder sachets |
| 77116 | Quinapril 5mg tablets (Alliance Healthcare (Distribution) Ltd) |
| 77129 | Ramipril 10mg capsules (Ennogen Pharma Ltd) |
| 77271 | Enalapril 20mg Tablet (Neo Laboratories Ltd) |
| 77315 | Enalapril 2.5mg/5ml oral solution |
| 77316 | Enalapril 4mg/5ml oral solution |
| 77345 | Innovace 10mg tablets (Dowelhurst Ltd) |
| 77361 | Capoten 25mg tablets (Waymade Healthcare Plc) |
| 77364 | Capoten 25mg tablets (Stephar (U.K.) Ltd) |
| 77378 | Zestril 10mg tablets (Dowelhurst Ltd) |
| 77400 | Capoten 25mg tablets (Mawdsley-Brooks & Company Ltd) |
| 77401 | Zestril 2.5mg tablets (Waymade Healthcare Plc) |
| 77402 | Zestril 5mg tablets (Dowelhurst Ltd) |
| 77407 | Zestril 5mg tablets (Waymade Healthcare Plc) |
| 77410 | Capoten 50mg tablets (Dowelhurst Ltd) |
| 77415 | Capoten 50mg tablets (Waymade Healthcare Plc) |
| 77457 | Capozide 25mg/50mg tablets (Dowelhurst Ltd) |
| 77486 | Ramipril 2.5mg tablets (Alliance Healthcare (Distribution) Ltd) |
| 77597 | Captopril 50mg/5ml oral suspension |
| 77615 | Ramipril 1.25mg/5ml oral solution |
| 77665 | Perindopril erbumine 8mg tablets (Somex Pharma) |
| 77690 | Enalapril 10mg tablets (IVAX Pharmaceuticals UK Ltd) |
| 77831 | Enalapril 500micrograms/5ml oral solution |
| 78169 | Ramipril 10mg tablets (IVAX Pharmaceuticals UK Ltd) |
| 78245 | Ramipril 2.5mg capsules (DE Pharmaceuticals) |
| 78252 | Lisinopril 20mg/5ml oral suspension |
| 78262 | Lisinopril 10mg / Hydrochlorothiazide 12.5mg tablets (Waymade Healthcare Plc) |
| 78524 | Lisinopril 10mg tablets (Tillomed Laboratories Ltd) |
| 78785 | Tritace 2.5mg Tablet (Sterwin Medicines) |
| 78808 | Lisinopril 5mg tablets (Ranbaxy (UK) Ltd) |
| 78887 | Enalapril 20mg / Hydrochlorothiazide 12.5mg tablets (A A H Pharmaceuticals Ltd) |
| 79005 | Perindopril erbumine 4mg tablets (Waymade Healthcare Plc) |
| 79006 | Perindopril erbumine 4mg tablets (Somex Pharma) |
| 79078 | Ramipril 10mg tablets (Niche Generics Ltd) |
| 79206 | Lisinopril 2.5mg tablets (Phoenix Healthcare Distribution Ltd) |
| 79334 | Captopril 500micrograms/5ml oral solution |
| 79361 | Lisinopril 2.5mg Tablet (Niche Generics Ltd) |
| 79380 | Ramipril 1.25mg Capsule (Sovereign Medical Ltd) |
| 79406 | Quinapril 10mg tablets (Actavis UK Ltd) |
| 79484 | Ramipril 1.25mg capsules (Sigma Pharmaceuticals Plc) |
| 79485 | Perindopril erbumine 8mg tablets (Kent Pharma (UK) Ltd) |
| 79531 | Perindopril tosilate 5mg tablets (Teva UK Ltd) |
| 79828 | Perindopril 4mg Tablet (Servier Laboratories Ltd) |
| 79849 | Ramipril 1.25mg capsules (Wockhardt UK Ltd) |
| 80051 | Enalapril 25mg/5ml oral suspension |
| 80208 | Coversyl Arginine 10mg tablets (DE Pharmaceuticals) |
| 80451 | Ramipril 1.25mg capsules (Ennogen Pharma Ltd) |
| 80498 | Enalapril oral liquid |
| 80520 | Ramipril 1.25mg capsules (Brown & Burk UK Ltd) |
| 80522 | Lisinopril 5mg/5ml oral solution sugar free (Alliance Healthcare (Distribution) Ltd) |
| 80853 | Perindopril erbumine 2mg tablets (Tillomed Laboratories Ltd) |
| 81049 | Perindopril erbumine 2mg/5ml oral suspension |
| 81148 | Lisinopril 10mg tablets (Mawdsley-Brooks & Company Ltd) |
| 81175 | Ecopace 50mg tablets (Advanz Pharma) |
| 81449 | Enalapril 1mg/5ml oral solution |
| 81590 | Co-zidocapt 25mg/50mg tablets (Tillomed Laboratories Ltd) |
| 81825 | Ramipril 2.5mg tablets (Viatris UK Healthcare Ltd) |
| 81877 | Zestril 20mg tablets (CST Pharma Ltd) |
| 81936 | Enalapril 20mg/5ml oral suspension |
| 82033 | Trandolapril 4mg capsules (Viatris UK Healthcare Ltd) |
| 82036 | Lisinopril 10mg / Hydrochlorothiazide 12.5mg tablets (A A H Pharmaceuticals Ltd) |
| 82348 | Trandolapril 500microgram capsules (Accord Healthcare Ltd) |
| 82363 | Enalapril 5mg tablets (Accord Healthcare Ltd) |
| 82455 | Trandolapril 2mg capsules (Accord Healthcare Ltd) |
| 82499 | Innovace 5mg tablets (Dowelhurst Ltd) |
| 82505 | Capozide 25mg/50mg tablets (Waymade Healthcare Plc) |
| 82515 | Innovace 20mg tablets (Dowelhurst Ltd) |
| 82557 | Accupro 5mg tablets (Waymade Healthcare Plc) |
| 82606 | Lisinopril 20mg tablets (Arrow Generics Ltd) |
| 82813 | Ramipril 2.5mg capsules (Crescent Pharma Ltd) |
| 83451 | Enalapril 5mg tablets (Almus Pharmaceuticals Ltd) |
| 84039 | Lisinopril 2.5mg tablets (Medihealth (Northern) Ltd) |
| 84153 | Ramipril 1.25mg capsules (Medihealth (Northern) Ltd) |
| 84294 | Lisinopril 5mg/5ml oral solution sugar free (Rosemont Pharmaceuticals Ltd) |
| 84750 | Lisinopril 10mg tablets (Lupin Healthcare (UK) Ltd) |
| 85046 | Lisinopril 10mg tablets (Medihealth (Northern) Ltd) |
| 85392 | Quinapril 40mg tablets (Teva UK Ltd) |
| 85546 | Lisinopril 20mg/5ml oral solution |
| 85631 | Lisoretic 10mg/12.5mg tablets (Bristol Laboratories Ltd) |
| 86076 | Ramipril 2.5mg tablets (Zentiva Pharma UK Ltd) |
| 86648 | Ramipril 2.5mg capsules (Milpharm Ltd) |
| 19690 | Verapamil 180mg modified-release / Trandolapril 2mg capsules |

Chronic Fatigue Syndrome

| medcode | readcode | readterm |
| --- | --- | --- |
| 4546 | F286.00 | Chronic fatigue syndrome |
| 1042 | R007400 | [D]Postviral (asthenic) syndrome |
| 7529 | F286.11 | CFS - Chronic fatigue syndrome |
| 6190 | F286.12 | Postviral fatigue syndrome |
| 1040 | F286.15 | Myalgic encephalomyelitis |
| 9127 | F286.14 | Post-viral fatigue syndrome |
| 6552 | F286.16 | ME - Myalgic encephalomyelitis |
| 9656 | Eu46011 | [X]Fatigue syndrome |
| 12411 | R007411 | [D]Post viral debility |
| 20414 | 1684.13 | C/O - postviral syndrome |
| 27877 | F286.13 | PVFS - Postviral fatigue syn |
| 99807 | 8HkW.00 | Referral to chronic fatigue syndrome specialist team |
| 97140 | 8Q1..00 | Activity management for chronic fatigue syndrome |
| 100414 | 8HlL.00 | Referral for chronic fatigue syndrome activity management |
| 98512 | F286000 | Mild chronic fatigue syndrome |
| 100464 | 8HkW.11 | Referral to myalgic encephalomyelitis specialist team |
| 97688 | 8E7B.00 | Graded exercise therapy |
| 97284 | F286100 | Moderate chronic fatigue syndrome |
| 98734 | F286200 | Severe chronic fatigue syndrome |
| 101140 | 8H7q000 | Referral for graded exercise therapy |
| 104407 | 8HlL.11 | Referral for myalgic encephalopathy activity management |

Coronary Heart Disease

| readcode | medcode | readterm |
| --- | --- | --- |
| G3...00 | 240 | Ischaemic heart disease |
| G3...11 | 24783 | Arteriosclerotic heart disease |
| G3...12 | 20416 | Atherosclerotic heart disease |
| G3...13 | 1792 | IHD - Ischaemic heart disease |
| G31..00 | 27951 | Other acute and subacute ischaemic heart disease |
| G310.00 | 23579 | Postmyocardial infarction syndrome |
| G310.11 | 15661 | Dressler's syndrome |
| G311.00 | 36523 | Preinfarction syndrome |
| G311.11 | 4656 | Crescendo angina |
| G311.12 | 39655 | Impending infarction |
| G311.13 | 1431 | Unstable angina |
| G311.14 | 19655 | Angina at rest |
| G311100 | 7347 | Unstable angina |
| G311200 | 17307 | Angina at rest |
| G311300 | 34328 | Refractory angina |
| G311500 | 11983 | Acute coronary syndrome |
| G311z00 | 54251 | Preinfarction syndrome NOS |
| G31y.00 | 9413 | Other acute and subacute ischaemic heart disease |
| G31y000 | 9276 | Acute coronary insufficiency |
| G31y200 | 39693 | Subendocardial ischaemia |
| G31y300 | 21844 | Transient myocardial ischaemia |
| G31yz00 | 27977 | Other acute and subacute ischaemic heart disease NOS |
| G32..00 | 4017 | Old myocardial infarction |
| G32..11 | 16408 | Healed myocardial infarction |
| G32..12 | 17464 | Personal history of myocardial infarction |
| G33..00 | 1430 | Angina pectoris |
| G330.00 | 20095 | Angina decubitus |
| G330000 | 18125 | Nocturnal angina |
| G330z00 | 29902 | Angina decubitus NOS |
| G331.00 | 12986 | Prinzmetal's angina |
| G331.11 | 11048 | Variant angina pectoris |
| G332.00 | 36854 | Coronary artery spasm |
| G33z.00 | 25842 | Angina pectoris NOS |
| G33z000 | 66388 | Status anginosus |
| G33z100 | 54535 | Stenocardia |
| G33z200 | 7696 | Syncope anginosa |
| G33z300 | 1414 | Angina on effort |
| G33z400 | 32450 | Ischaemic chest pain |
| G33z500 | 9555 | Post infarct angina |
| G33z600 | 26863 | New onset angina |
| G33z700 | 12804 | Stable angina |
| G33zz00 | 28554 | Angina pectoris NOS |
| G34..00 | 28138 | Other chronic ischaemic heart disease |
| G340.00 | 5413 | Coronary atherosclerosis |
| G340.11 | 1655 | Triple vessel disease of the heart |
| G340.12 | 1344 | Coronary artery disease |
| G340000 | 3999 | Single coronary vessel disease |
| G340100 | 5254 | Double coronary vessel disease |
| G342.00 | 36609 | Atherosclerotic cardiovascular disease |
| G343.00 | 7320 | Ischaemic cardiomyopathy |
| G344.00 | 29421 | Silent myocardial ischaemia |
| G34y.00 | 34633 | Other specified chronic ischaemic heart disease |
| G34y000 | 24540 | Chronic coronary insufficiency |
| G34y100 | 23078 | Chronic myocardial ischaemia |
| G34yz00 | 35713 | Other specified chronic ischaemic heart disease NOS |
| G34z.00 | 15754 | Other chronic ischaemic heart disease NOS |
| G34z000 | 18889 | Asymptomatic coronary heart disease |
| G3y..00 | 22383 | Other specified ischaemic heart disease |
| G3z..00 | 1676 | Ischaemic heart disease NOS |
| Gyu3.00 | 52517 | [X]Ischaemic heart diseases |
| Gyu3200 | 68401 | [X]Other forms of acute ischaemic heart disease |
| Gyu3300 | 47637 | [X]Other forms of chronic ischaemic heart disease |

Depression and Anxiety

| readcode | medcode | readterm |
| --- | --- | --- |
| 1465 | 2716 | NA |
| 1B17.00 | 1996 | Depressed |
| 1B17.00 | 1996 | Depressed |
| 1B17.11 | 4824 | C/O - feeling depressed |
| 1B1U.00 | 9796 | Symptoms of depression |
| 1B1U.11 | 10438 | Depressive symptoms |
| 1BT..00 | 10015 | Depressed mood |
| 212S.00 | 19439 | Depression resolved |
| 212S.00 | 19439 | Depression resolved |
| 2257 | 1908 | NA |
| 62T1.00 | 2923 | Puerperal depression |
| 62T1.00 | 2923 | Puerperal depression |
| 8CAa.00 | 30483 | Patient given advice about management of depression |
| 8HHq.00 | 32841 | Referral for guided self-help for depression |
| 9H90.00 | 12399 | Depression annual review |
| 9H91.00 | 12122 | Depression medication review |
| 9H92.00 | 30405 | Depression interim review |
| 9HA0.00 | 42931 | On depression register |
| 9HA1.00 | 44936 | Removed from depression register |
| 9HA1.00 | 44936 | Removed from depression register |
| E112.00 | 10610 | Single major depressive episode |
| E112.00 | 10610 | Single major depressive episode |
| E112.11 | 5879 | Agitated depression |
| E112.11 | 5879 | Agitated depression |
| E112.12 | 6546 | Endogenous depression first episode |
| E112.12 | 6546 | Endogenous depression first episode |
| E112.13 | 6950 | Endogenous depression first episode |
| E112.13 | 6950 | Endogenous depression first episode |
| E112.14 | 595 | Endogenous depression |
| E112.14 | 595 | Endogenous depression |
| E112000 | 34390 | Single major depressive episode, unspecified |
| E112000 | 34390 | Single major depressive episode, unspecified |
| E112100 | 16506 | Single major depressive episode, mild |
| E112100 | 16506 | Single major depressive episode, mild |
| E112200 | 15155 | Single major depressive episode, moderate |
| E112200 | 15155 | Single major depressive episode, moderate |
| E112300 | 15219 | Single major depressive episode, severe, without psychosis |
| E112300 | 15219 | Single major depressive episode, severe, without psychosis |
| E112500 | 43324 | Single major depressive episode, partial or unspec remission |
| E112500 | 43324 | Single major depressive episode, partial or unspec remission |
| E112600 | 57409 | Single major depressive episode, in full remission |
| E112600 | 57409 | Single major depressive episode, in full remission |
| E112z00 | 7011 | Single major depressive episode NOS |
| E112z00 | 7011 | Single major depressive episode NOS |
| E113.00 | 15099 | Recurrent major depressive episode |
| E113.00 | 15099 | Recurrent major depressive episode |
| E113.11 | 6932 | Endogenous depression - recurrent |
| E113.11 | 6932 | Endogenous depression - recurrent |
| E113000 | 35671 | Recurrent major depressive episodes, unspecified |
| E113000 | 35671 | Recurrent major depressive episodes, unspecified |
| E113100 | 29342 | Recurrent major depressive episodes, mild |
| E113100 | 29342 | Recurrent major depressive episodes, mild |
| E113200 | 14709 | Recurrent major depressive episodes, moderate |
| E113200 | 14709 | Recurrent major depressive episodes, moderate |
| E113300 | 25697 | Recurrent major depressive episodes, severe, no psychosis |
| E113300 | 25697 | Recurrent major depressive episodes, severe, no psychosis |
| E113500 | 56273 | Recurrent major depressive episodes,partial/unspec remission |
| E113500 | 56273 | Recurrent major depressive episodes,partial/unspec remission |
| E113600 | 55384 | Recurrent major depressive episodes, in full remission |
| E113600 | 55384 | Recurrent major depressive episodes, in full remission |
| E113700 | 6482 | Recurrent depression |
| E113700 | 6482 | Recurrent depression |
| E113z00 | 25563 | Recurrent major depressive episode NOS |
| E113z00 | 25563 | Recurrent major depressive episode NOS |
| E118.00 | 10825 | Seasonal affective disorder |
| E118.00 | 10825 | Seasonal affective disorder |
| E11y200 | 27491 | Atypical depressive disorder |
| E11y200 | 27491 | Atypical depressive disorder |
| E11z200 | 9183 | Masked depression |
| E11z200 | 9183 | Masked depression |
| E135.00 | 1055 | Agitated depression |
| E135.00 | 1055 | Agitated depression |
| E200300 | 655 | Anxiety with depression |
| E200300 | 655 | Anxiety with depression |
| E204.00 | 1131 | Neurotic depression reactive type |
| E204.00 | 1131 | Neurotic depression reactive type |
| E204.11 | 2639 | Postnatal depression |
| E204.11 | 2639 | Postnatal depression |
| E211200 | 10455 | Depressive personality disorder |
| E290.00 | 1533 | Brief depressive reaction |
| E290.00 | 1533 | Brief depressive reaction |
| E290z00 | 36246 | Brief depressive reaction NOS |
| E290z00 | 36246 | Brief depressive reaction NOS |
| E291.00 | 16632 | Prolonged depressive reaction |
| E291.00 | 16632 | Prolonged depressive reaction |
| E2B..00 | 324 | Depressive disorder NEC |
| E2B..00 | 324 | Depressive disorder NEC |
| E2B0.00 | 2972 | Postviral depression |
| E2B0.00 | 2972 | Postviral depression |
| E2B1.00 | 4323 | Chronic depression |
| E2B1.00 | 4323 | Chronic depression |
| Eu32.00 | 4639 | [X]Depressive episode |
| Eu32.00 | 4639 | [X]Depressive episode |
| Eu32.11 | 9055 | [X]Single episode of depressive reaction |
| Eu32.11 | 9055 | [X]Single episode of depressive reaction |
| Eu32.12 | 18510 | [X]Single episode of psychogenic depression |
| Eu32.12 | 18510 | [X]Single episode of psychogenic depression |
| Eu32.13 | 7604 | [X]Single episode of reactive depression |
| Eu32.13 | 7604 | [X]Single episode of reactive depression |
| Eu32000 | 11717 | [X]Mild depressive episode |
| Eu32000 | 11717 | [X]Mild depressive episode |
| Eu32100 | 9211 | [X]Moderate depressive episode |
| Eu32100 | 9211 | [X]Moderate depressive episode |
| Eu32200 | 9667 | [X]Severe depressive episode without psychotic symptoms |
| Eu32200 | 9667 | [X]Severe depressive episode without psychotic symptoms |
| Eu32211 | 41989 | [X]Single episode agitated depressn w'out psychotic symptoms |
| Eu32211 | 41989 | [X]Single episode agitated depressn w'out psychotic symptoms |
| Eu32212 | 22806 | [X]Single episode major depression w'out psychotic symptoms |
| Eu32212 | 22806 | [X]Single episode major depression w'out psychotic symptoms |
| Eu32213 | 59386 | [X]Single episode vital depression w'out psychotic symptoms |
| Eu32213 | 59386 | [X]Single episode vital depression w'out psychotic symptoms |
| Eu32400 | 10667 | [X]Mild depression |
| Eu32400 | 10667 | [X]Mild depression |
| Eu32y00 | 6854 | [X]Other depressive episodes |
| Eu32y00 | 6854 | [X]Other depressive episodes |
| Eu32y11 | 10720 | [X]Atypical depression |
| Eu32y11 | 10720 | [X]Atypical depression |
| Eu32y12 | 56609 | [X]Single episode of masked depression NOS |
| Eu32y12 | 56609 | [X]Single episode of masked depression NOS |
| Eu32z00 | 2970 | [X]Depressive episode, unspecified |
| Eu32z00 | 2970 | [X]Depressive episode, unspecified |
| Eu32z11 | 543 | [X]Depression NOS |
| Eu32z11 | 543 | [X]Depression NOS |
| Eu32z12 | 3291 | [X]Depressive disorder NOS |
| Eu32z12 | 3291 | [X]Depressive disorder NOS |
| Eu32z13 | 28248 | [X]Prolonged single episode of reactive depression |
| Eu32z13 | 28248 | [X]Prolonged single episode of reactive depression |
| Eu32z14 | 5987 | [X] Reactive depression NOS |
| Eu32z14 | 5987 | [X] Reactive depression NOS |
| Eu33.00 | 3292 | [X]Recurrent depressive disorder |
| Eu33.00 | 3292 | [X]Recurrent depressive disorder |
| Eu33.11 | 8851 | [X]Recurrent episodes of depressive reaction |
| Eu33.11 | 8851 | [X]Recurrent episodes of depressive reaction |
| Eu33.12 | 19696 | [X]Recurrent episodes of psychogenic depression |
| Eu33.12 | 19696 | [X]Recurrent episodes of psychogenic depression |
| Eu33.13 | 8902 | [X]Recurrent episodes of reactive depression |
| Eu33.13 | 8902 | [X]Recurrent episodes of reactive depression |
| Eu33.14 | 28756 | [X]Seasonal depressive disorder |
| Eu33.14 | 28756 | [X]Seasonal depressive disorder |
| Eu33.15 | 8826 | [X]SAD - Seasonal affective disorder |
| Eu33.15 | 8826 | [X]SAD - Seasonal affective disorder |
| Eu33000 | 29784 | [X]Recurrent depressive disorder, current episode mild |
| Eu33000 | 29784 | [X]Recurrent depressive disorder, current episode mild |
| Eu33100 | 29520 | [X]Recurrent depressive disorder, current episode moderate |
| Eu33100 | 29520 | [X]Recurrent depressive disorder, current episode moderate |
| Eu33200 | 33469 | [X]Recurr depress disorder cur epi severe without psyc sympt |
| Eu33200 | 33469 | [X]Recurr depress disorder cur epi severe without psyc sympt |
| Eu33211 | 11329 | [X]Endogenous depression without psychotic symptoms |
| Eu33211 | 11329 | [X]Endogenous depression without psychotic symptoms |
| Eu33212 | 11252 | [X]Major depression, recurrent without psychotic symptoms |
| Eu33212 | 11252 | [X]Major depression, recurrent without psychotic symptoms |
| Eu33214 | 73991 | [X]Vital depression, recurrent without psychotic symptoms |
| Eu33214 | 73991 | [X]Vital depression, recurrent without psychotic symptoms |
| Eu33400 | 22116 | [X]Recurrent depressive disorder, currently in remission |
| Eu33400 | 22116 | [X]Recurrent depressive disorder, currently in remission |
| Eu33y00 | 47731 | [X]Other recurrent depressive disorders |
| Eu33y00 | 47731 | [X]Other recurrent depressive disorders |
| Eu33z00 | 44300 | [X]Recurrent depressive disorder, unspecified |
| Eu33z00 | 44300 | [X]Recurrent depressive disorder, unspecified |
| Eu33z11 | 36616 | [X]Monopolar depression NOS |
| Eu33z11 | 36616 | [X]Monopolar depression NOS |
| Eu34100 | 7953 | [X]Dysthymia |
| Eu34100 | 7953 | [X]Dysthymia |
| Eu34111 | 8584 | [X]Depressive neurosis |
| Eu34111 | 8584 | [X]Depressive neurosis |
| Eu34113 | 7737 | [X]Neurotic depression |
| Eu34113 | 7737 | [X]Neurotic depression |
| Eu34114 | 15220 | [X]Persistant anxiety depression |
| Eu34114 | 15220 | [X]Persistant anxiety depression |
| Eu3y111 | 19054 | [X]Recurrent brief depressive episodes |
| Eu3y111 | 19054 | [X]Recurrent brief depressive episodes |
| Eu41200 | 11913 | [X]Mixed anxiety and depressive disorder |
| Eu41200 | 11913 | [X]Mixed anxiety and depressive disorder |
| Eu41211 | 7749 | [X]Mild anxiety depression |
| Eu41211 | 7749 | [X]Mild anxiety depression |
| Eu53011 | 13307 | [X]Postnatal depression NOS |
| Eu53011 | 13307 | [X]Postnatal depression NOS |
| Eu53012 | 4979 | [X]Postpartum depression NOS |
| Eu53012 | 4979 | [X]Postpartum depression NOS |
| Eu92000 | 32845 | [X]Depressive conduct disorder |
| Eu92000 | 32845 | [X]Depressive conduct disorder |
| 2258 | 13124 | NA |
| 225J.00 | 19000 | O/E - panic attack |
| 8G52.00 | 63521 | Antiphobic therapy |
| 8G94.00 | 9125 | Anxiety management training |
| 8HHp.00 | 28925 | Referral for guided self-help for anxiety |
| E2...00 | 9686 | Neurotic, personality and other nonpsychotic disorders |
| E20..00 | 5249 | Neurotic disorders |
| E200.00 | 636 | Anxiety states |
| E200000 | 6939 | Anxiety state unspecified |
| E200100 | 4069 | Panic disorder |
| E200111 | 462 | Panic attack |
| E200200 | 4659 | Generalised anxiety disorder |
| E200300 | 655 | Anxiety with depression |
| E200400 | 1758 | Chronic anxiety |
| E200500 | 4634 | Recurrent anxiety |
| E200z00 | 4534 | Anxiety state NOS |
| E201.00 | 2188 | Hysteria |
| E201000 | 41572 | Hysteria unspecified |
| E201100 | 3438 | Hysterical blindness |
| E201200 | 44739 | Hysterical deafness |
| E201300 | 23598 | Hysterical tremor |
| E201400 | 34696 | Hysterical paralysis |
| E201500 | 16484 | Hysterical seizures |
| E201511 | 33702 | Fit - hysterical |
| E201600 | 15431 | Other conversion disorder |
| E201611 | 46399 | Astasia - abasia, hysterical |
| E201612 | 4143 | Globus hystericus |
| E201700 | 4269 | Hysterical amnesia |
| E201800 | 4775 | Hysterical fugue |
| E201900 | 43302 | Multiple personality |
| E201A00 | 23490 | Dissociative reaction unspecified |
| E201B00 | 29322 | Compensation neurosis |
| E201C00 | 24525 | Phantom pregnancy |
| E201z00 | 23354 | Hysteria NOS |
| E201z11 | 4105 | Aphonia - hysterical |
| E201z12 | 24638 | Ataxia - hysterical |
| E201z13 | 40066 | Ganser's syndrome - hysterical |
| E202.00 | 1907 | Phobic disorders |
| E202.11 | 16638 | Social phobic disorders |
| E202.12 | 9944 | Phobic anxiety |
| E202000 | 2300 | Phobia unspecified |
| E202100 | 3076 | Agoraphobia with panic attacks |
| E202200 | 12838 | Agoraphobia without mention of panic attacks |
| E202300 | 16199 | Social phobia, fear of eating in public |
| E202400 | 31957 | Social phobia, fear of public speaking |
| E202500 | 18603 | Social phobia, fear of public washing |
| E202600 | 28106 | Acrophobia |
| E202700 | 28938 | Animal phobia |
| E202800 | 1723 | Claustrophobia |
| E202900 | 31672 | Fear of crowds |
| E202A00 | 4167 | Fear of flying |
| E202B00 | 1510 | Cancer phobia |
| E202C00 | 2366 | Dental phobia |
| E202D00 | 10390 | Fear of death |
| E202E00 | 6071 | Fear of pregnancy |
| E202z00 | 14729 | Phobic disorder NOS |
| E202z11 | 38543 | Weight fixation |
| E203.00 | 3208 | Obsessive-compulsive disorders |
| E203.11 | 47365 | Anancastic neurosis |
| E203000 | 5678 | Compulsive neurosis |
| E203100 | 2030 | Obsessional neurosis |
| E203z00 | 15566 | Obsessive-compulsive disorder NOS |
| E205.00 | 3361 | Neurasthenia - nervous debility |
| E205.11 | 1582 | Nervous exhaustion |
| E206.00 | 5305 | Depersonalisation syndrome |
| E207.00 | 966 | Hypochondriasis |
| E20y.00 | 42000 | Other neurotic disorders |
| E20y000 | 15321 | Somatization disorder |
| E20y011 | 56941 | Briquet's disorder |
| E20y100 | 3685 | Writer's cramp neurosis |
| E20y200 | 39518 | Other occupational neurosis |
| E20y300 | 72171 | Psychasthenic neurosis |
| E20yz00 | 43050 | Other neurotic disorder NOS |
| E20z.00 | 14780 | Neurotic disorder NOS |
| E20z.11 | 791 | Nervous breakdown |
| E26..00 | 4199 | Physiological malfunction arising from mental factors |
| E260.00 | 44212 | Psychogenic musculoskeletal symptoms |
| E260000 | 48561 | Psychogenic paralysis |
| E260100 | 56800 | Psychogenic torticollis |
| E260z00 | 15035 | Psychogenic musculoskeletal symptoms NOS |
| E261.00 | 38134 | Psychogenic respiratory symptoms |
| E261000 | 32034 | Psychogenic air hunger |
| E261100 | 15483 | Psychogenic cough |
| E261200 | 47809 | Psychogenic hiccough |
| E261300 | 20053 | Psychogenic hyperventilation |
| E261400 | 23413 | Psychogenic yawning |
| E261500 | 41615 | Psychogenic aphonia |
| E261z00 | 34664 | Psychogenic respiratory symptom NOS |
| E262.00 | 29448 | Psychogenic cardiovascular symptoms |
| E262000 | 15292 | Cardiac neurosis |
| E262200 | 15284 | Neurocirculatory asthenia |
| E262300 | 30961 | Psychogenic cardiovascular disorder |
| E262z00 | 15034 | Psychogenic cardiovascular symptom NOS |
| E263.00 | 29461 | Psychogenic skin symptoms |
| E263000 | 15959 | Psychogenic pruritus |
| E263z00 | 15224 | Psychogenic skin symptoms NOS |
| E264.00 | 31422 | Psychogenic gastrointestinal tract symptoms |
| E264.11 | 37695 | Globus abdominalis |
| E264000 | 4963 | Psychogenic aerophagy |
| E264011 | 23774 | Air swallowing - excessive |
| E264200 | 2871 | Cyclical vomiting - psychogenic |
| E264300 | 15371 | Psychogenic diarrhoea |
| E264311 | 10158 | Spurious diarrhoea |
| E264400 | 3869 | Psychogenic dyspepsia |
| E264500 | 15939 | Psychogenic constipation |
| E264z00 | 71437 | Psychogenic gastrointestinal tract symptom NOS |
| E265.00 | 68379 | Psychogenic genitourinary tract symptoms |
| E265100 | 20109 | Psychogenic vaginismus |
| E265200 | 44547 | Psychogenic dysmenorrhea |
| E265300 | 55781 | Psychogenic dysuria |
| E265z00 | 73547 | Psychogenic genitourinary tract symptom NOS |
| E267.00 | 89237 | Psychogenic symptom of special sense organ |
| E26y.00 | 62400 | Other psychogenic malfunction |
| E26y000 | 10001 | Bruxism (teeth grinding) |
| E26yz00 | 96391 | Other psychogenic malfunction NOS |
| E26z.00 | 5067 | Psychosomatic disorder NOS |
| E278.00 | 40311 | Psychalgia |
| E278000 | 53766 | Psychogenic pain unspecified |
| E278100 | 191 | Tension headache |
| E278111 | 9999 | Muscular headache |
| E278200 | 45205 | Psychogenic backache |
| E278z00 | 54373 | Psychalgia NOS |
| E28..00 | 276 | Acute reaction to stress |
| E28..11 | 43550 | Combat fatigue |
| E280.00 | 11940 | Acute panic state due to acute stress reaction |
| E281.00 | 42737 | Acute fugue state due to acute stress reaction |
| E282.00 | 15551 | Acute stupor state due to acute stress reaction |
| E283.00 | 38640 | Other acute stress reactions |
| E283000 | 20245 | Acute situational disturbance |
| E283100 | 24847 | Acute posttrauma stress state |
| E283z00 | 29707 | Other acute stress reaction NOS |
| E284.00 | 23869 | Stress reaction causing mixed disturbance of emotion/conduct |
| E28z.00 | 26138 | Acute stress reaction NOS |
| E28z.11 | 28129 | Examination fear |
| E28z.12 | 20802 | Flying phobia |
| E28z.13 | 27742 | Stage fright |
| E29..00 | 2826 | Adjustment reaction |
| E290000 | 2775 | Grief reaction |
| E290011 | 10734 | Bereavement reaction |
| E292.00 | 24212 | Adjustment reaction, predominant disturbance other emotions |
| E292000 | 6221 | Separation anxiety disorder |
| E292100 | 23327 | Adolescent emancipation disorder |
| E292200 | 54658 | Early adult emancipation disorder |
| E292300 | 62193 | Specific academic or work inhibition |
| E292311 | 67304 | Specific academic or work inhibition |
| E292312 | 28302 | Specific work inhibition |
| E292400 | 56924 | Adjustment reaction with anxious mood |
| E292500 | 58013 | Culture shock |
| E292y00 | 48588 | Adjustment reaction with mixed disturbance of emotion |
| E292z00 | 15665 | Adjustment reaction with disturbance of other emotion NOS |
| E293.00 | 16415 | Adjustment reaction with predominant disturbance of conduct |
| E293000 | 6075 | Adjustment reaction with aggression |
| E293100 | 35914 | Adjustment reaction with antisocial behaviour |
| E293200 | 66398 | Adjustment reaction with destructiveness |
| E294.00 | 45603 | Adjustment reaction with disturbance emotion and conduct |
| E29y.00 | 41455 | Other adjustment reactions |
| E29y000 | 53362 | Concentration camp syndrome |
| E29y100 | 32387 | Other post-traumatic stress disorder |
| E29y200 | 35632 | Adjustment reaction with physical symptoms |
| E29y300 | 7716 | Elective mutism due to an adjustment reaction |
| E29y400 | 27390 | Adjustment reaction due to hospitalisation |
| E29y500 | 19921 | Other adjustment reaction with withdrawal |
| E29yz00 | 23462 | Other adjustment reactions NOS |
| E29z.00 | 37669 | Adjustment reaction NOS |
| E2y..00 | 50106 | Other specified neuroses or other mental disorders |
| E2z..00 | 42410 | Neuroses or other mental disorder NOS |
| Eu05400 | 20773 | [X]Organic anxiety disorder |
| Eu34114 | 15220 | [X]Persistant anxiety depression |
| Eu4..00 | 23808 | [X]Neurotic, stress - related and somoform disorders |
| Eu40.00 | 9386 | [X]Phobic anxiety disorders |
| Eu40000 | 2571 | [X]Agoraphobia |
| Eu40011 | 16729 | [X]Agoraphobia without history of panic disorder |
| Eu40012 | 14890 | [X]Panic disorder with agoraphobia |
| Eu40100 | 11602 | [X]Social phobias |
| Eu40112 | 42788 | [X]Social neurosis |
| Eu40200 | 9785 | [X]Specific (isolated) phobias |
| Eu40211 | 67965 | [X]Acrophobia |
| Eu40212 | 18248 | [X]Animal phobias |
| Eu40213 | 11280 | [X]Claustrophobia |
| Eu40214 | 12635 | [X]Simple phobia |
| Eu40300 | 12508 | [X]Needle phobia |
| Eu40y00 | 27685 | [X]Other phobic anxiety disorders |
| Eu40z00 | 34064 | [X]Phobic anxiety disorder, unspecified |
| Eu40z11 | 7222 | [X]Phobia NOS |
| Eu40z12 | 67898 | [X]Phobic state NOS |
| Eu41.00 | 5385 | [X]Other anxiety disorders |
| Eu41000 | 8205 | [X]Panic disorder [episodic paroxysmal anxiety] |
| Eu41011 | 6408 | [X]Panic attack |
| Eu41012 | 4081 | [X]Panic state |
| Eu41100 | 10344 | [X]Generalized anxiety disorder |
| Eu41111 | 962 | [X]Anxiety neurosis |
| Eu41112 | 35825 | [X]Anxiety reaction |
| Eu41113 | 50191 | [X]Anxiety state |
| Eu41200 | 11913 | [X]Mixed anxiety and depressive disorder |
| Eu41211 | 7749 | [X]Mild anxiety depression |
| Eu41300 | 44321 | [X]Other mixed anxiety disorders |
| Eu41y00 | 24066 | [X]Other specified anxiety disorders |
| Eu41y11 | 28167 | [X]Anxiety hysteria |
| Eu41z00 | 23838 | [X]Anxiety disorder, unspecified |
| Eu41z11 | 25638 | [X]Anxiety NOS |
| Eu42.00 | 5304 | [X]Obsessive - compulsive disorder |
| Eu42.11 | 24251 | [X]Anankastic neurosis |
| Eu42.12 | 21836 | [X]Obsessive-compulsive neurosis |
| Eu42000 | 20634 | [X]Predominantly obsessional thoughts or ruminations |
| Eu42100 | 22019 | [X]Predominantly compulsive acts [obsessional rituals] |
| Eu42200 | 18399 | [X]Mixed obsessional thoughts and acts |
| Eu42y00 | 38809 | [X]Other obsessive-compulsive disorders |
| Eu42z00 | 22721 | [X]Obsessive-compulsive disorder, unspecified |
| Eu43.00 | 11098 | [X]Reaction to severe stress, and adjustment disorders |
| Eu43000 | 11607 | [X]Acute stress reaction |
| Eu43011 | 36374 | [X]Acute crisis reaction |
| Eu43012 | 10535 | [X]Acute reaction to stress |
| Eu43013 | 70779 | [X]Combat fatigue |
| Eu43014 | 21559 | [X]Crisis state |
| Eu43015 | 7813 | [X]Psychic shock |
| Eu43100 | 4171 | [X]Post - traumatic stress disorder |
| Eu43111 | 32182 | [X]Traumatic neurosis |
| Eu43200 | 11336 | [X]Adjustment disorders |
| Eu43211 | 36228 | [X]Culture shock |
| Eu43212 | 21197 | [X]Grief reaction |
| Eu43213 | 41235 | [X]Hospitalism in children |
| Eu43300 | 101785 | [X]Acute post-traumatic stress disorder follow military comb |
| Eu43400 | 101725 | [X]Chron post-traumatic stress disorder follow military comb |
| Eu43y00 | 21753 | [X]Other reactions to severe stress |
| Eu43z00 | 31515 | [X]Reaction to severe stress, unspecified |
| Eu44.00 | 22136 | [X]Dissociative [conversion] disorders |
| Eu44.11 | 35311 | [X]Conversion hysteria |
| Eu44.12 | 52161 | [X]Conversion reaction |
| Eu44.13 | 27588 | [X]Hysteria |
| Eu44000 | 40994 | [X]Dissociative amnesia |
| Eu44100 | 39826 | [X]Dissociative fugue |
| Eu44200 | 56141 | [X]Dissociative stupor |
| Eu44300 | 39747 | [X]Trance and possession disorders |
| Eu44400 | 34978 | [X]Dissociative motor disorders |
| Eu44411 | 12147 | [X]Psychogenic aphonia |
| Eu44412 | 18801 | [X]Psychogenic dysphonia |
| Eu44500 | 56966 | [X]Dissociative convulsions |
| Eu44511 | 11354 | [X]Pseudoseizures |
| Eu44600 | 27633 | [X]Dissociative anaesthesia and sensory loss |
| Eu44611 | 50121 | [X]Psychogenic deafness |
| Eu44700 | 88758 | [X]Mixed dissociative [conversion] disorders |
| Eu44y00 | 64166 | [X]Other dissociative [conversion] disorders |
| Eu44y11 | 46567 | [X]Ganser's syndrome |
| Eu44y12 | 39919 | [X]Multiple personality |
| Eu44y13 | 16988 | [X]Psychogenic confusion |
| Eu44y14 | 68259 | [X]Psychogenic twilight state |
| Eu44z00 | 48906 | [X]Dissociative [conversion] disorder, unspecified |
| Eu45.00 | 18049 | [X]Somatoform disorders |
| Eu45000 | 24439 | [X]Somatization disorder |
| Eu45011 | 23704 | [X]Multiple psychosomatic disorder |
| Eu45012 | 59682 | [X]Briquet's syndrome |
| Eu45100 | 57877 | [X]Undifferentiated somatoform disorder |
| Eu45111 | 34735 | [X]Undifferentiated psychosomatic disorder |
| Eu45200 | 7537 | [X]Hypochondriacal disorder |
| Eu45211 | 12626 | [X]Body dysmorphic disorder |
| Eu45212 | 30680 | [X]Dysmorphophobia nondelusional |
| Eu45213 | 24264 | [X]Hypochondriacal neurosis |
| Eu45214 | 10870 | [X]Hypochondriasis |
| Eu45215 | 66806 | [X]Nosophobia |
| Eu45300 | 41038 | [X]Somatoform autonomic dysfunction |
| Eu45311 | 44269 | [X]Cardiac neurosis |
| Eu45312 | 32632 | [X]Da Costa's syndrome |
| Eu45313 | 63259 | [X]Gastric neurosis |
| Eu45314 | 50793 | [X]Neurocirculatory asthenia |
| Eu45316 | 12715 | [X]Psychogenic cough |
| Eu45317 | 43316 | [X]Psychogenic diarrhoea |
| Eu45318 | 16714 | [X]Psychogenic dyspepsia |
| Eu45319 | 53122 | [X]Psychogenic dysuria |
| Eu45320 | 47698 | [X]Psychogenic flatulence |
| Eu45321 | 53737 | [X]Psychogenic hiccough |
| Eu45322 | 12830 | [X]Psychogenic hyperventilat |
| Eu45323 | 17081 | [X]Psychogenic freq micturit |
| Eu45324 | 16560 | [X]Psychogenic IBS |
| Eu45325 | 93067 | [X]Psychogenic pylorospasm |
| Eu45400 | 30179 | [X]Persistent somatoform pain disorder |
| Eu45411 | 54382 | [X]Psychalgia |
| Eu45412 | 38521 | [X]Psychogenic backache |
| Eu45413 | 29329 | [X]Psychogenic headache |
| Eu45414 | 36040 | [X]Somatoform pain disorder |
| Eu45500 | 12453 | [X]Globus pharyngeus |
| Eu45511 | 100086 | [X]Globus hystericus |
| Eu45y00 | 62002 | [X]Other somatoform disorders |
| Eu45y11 | 46925 | [X]Psychogenic dysmenorrhoea |
| Eu45y12 | 20906 | [X]Globus hystericus |
| Eu45y13 | 16679 | [X]Psychogenic pruritis |
| Eu45y14 | 36009 | [X]Psychogenic torticollis |
| Eu45y15 | 47570 | [X]Teeth-grinding |
| Eu45z00 | 48671 | [X]Somatoform disorder, unspecified |
| Eu45z11 | 19242 | [X]Psychosomatic disorder NOS |
| Eu46.00 | 28090 | [X]Other neurotic disorders |
| Eu46000 | 16561 | [X]Neurasthenia |
| Eu46100 | 9265 | [X]Depersonalization - derealization syndrome |
| Eu46y00 | 44331 | [X]Other specified neurotic disorders |
| Eu46y11 | 100116 | [X]Briquet's disorder |
| Eu46y12 | 47367 | [X]Dhat syndrome |
| Eu46y13 | 11339 | [X]Occupational neurosis, including writer's cramp |
| Eu46y14 | 61753 | [X]Psychasthenia |
| Eu46y15 | 90597 | [X]Psychasthenia neurosis |
| Eu46y16 | 44586 | [X]Psychogenic syncope |
| Eu46z00 | 49628 | [X]Neurotic disorder, unspecified |
| Eu46z11 | 21431 | [X]Neurosis NOS |
| Eu51511 | 17687 | [X]Dream anxiety disorder |
| Eu93000 | 18032 | [X]Separation anxiety disorder of childhood |
| Eu93100 | 24351 | [X]Phobic anxiety disorder of childhood |
| Eu93200 | 29907 | [X]Social anxiety disorder of childhood |
| Eu93y12 | 61430 | [X]Childhood overanxious disorder |
| Z4L1.00 | 7999 | Anxiety counselling |
| ZS7C700 | 99609 | Post-traumatic mutism |

Heart Failure

| readcode | medcode | readterm |  |
| --- | --- | --- | --- |
| 1O1..00 | 9913 | Heart failure confirmed |  |
| G1yz100 | 22262 | Rheumatic left ventricular failure |  |
| G232.00 | 21837 | Hypertensive heart&renal dis wth (congestive) heart failure | |
| G234.00 | 57987 | Hyperten heart&renal dis+both(congestv)heart and renal fail | |
| G58..00 | 2062 | Heart failure |  |
| G58..11 | 1223 | Cardiac failure |  |
| G580.00 | 398 | Congestive heart failure |  |
| G580.11 | 2906 | Congestive cardiac failure |  |
| G580.12 | 10079 | Right heart failure |  |
| G580.13 | 10154 | Right ventricular failure |  |
| G580.14 | 9524 | Biventricular failure |  |
| G580000 | 23707 | Acute congestive heart failure |  |
| G580100 | 32671 | Chronic congestive heart failure |  |
| G580200 | 27884 | Decompensated cardiac failure |  |
| G580300 | 11424 | Compensated cardiac failure |  |
| G581.00 | 884 | Left ventricular failure |  |
| G581.11 | 23481 | Asthma - cardiac |  |
| G581.12 | 43618 | Pulmonary oedema - acute |  |
| G581.13 | 5942 | Impaired left ventricular function |  |
| G581000 | 5255 | Acute left ventricular failure |  |
| G582.00 | 27964 | Acute heart failure |  |
| G58z.00 | 4024 | Heart failure NOS |  |
| G58z.12 | 17278 | Cardiac failure NOS |  |
| Q48y100 | 20822 | Congenital cardiac failure |  |
| Q490.00 | 23566 | Neonatal cardiac failure |  |

Hypertension

| readcode | medcode | readterm |
| --- | --- | --- |
| 2126100 | 3269 | Hypertension resolved |
| 2126100 | 3269 | Hypertension resolved |
| 212K.00 | 19342 | Hypertension resolved |
| 212K.00 | 19342 | Hypertension resolved |
| 662..12 | 4444 | Hypertension monitoring |
| 662b.00 | 18590 | Moderate hypertension control |
| 662c.00 | 18482 | Hypertension six month review |
| 662d.00 | 19070 | Hypertension annual review |
| 662F.00 | 21826 | Hypertension treatm. started |
| 662G.00 | 13188 | Hypertensive treatm.changed |
| 662O.00 | 3425 | On treatment for hypertension |
| 662P.00 | 13186 | Hypertension monitoring |
| 8B26.00 | 18057 | Antihypertensive therapy |
| 8BL0.00 | 11056 | Patient on maximal tolerated antihypertensive therapy |
| 8CR4.00 | 12680 | Hypertension clinical management plan |
| 8HT5.00 | 5513 | Referral to hypertension clinic |
| 9N03.00 | 4344 | Seen in hypertension clinic |
| 9N1y200 | 27634 | Seen in hypertension clinic |
| 9OI1.00 | 45149 | Attends hypertension monitor. |
| 9OIA.00 | 36305 | Hypertension monitor.chck done |
| 9OIA.11 | 24127 | Hypertension monitored |
| F404200 | 37086 | Blind hypertensive eye |
| F421300 | 6702 | Hypertensive retinopathy |
| G2...00 | 204 | Hypertensive disease |
| G2...00 | 204 | Hypertensive disease |
| G2...11 | 8732 | BP - hypertensive disease |
| G2...11 | 8732 | BP - hypertensive disease |
| G20..00 | 799 | Essential hypertension |
| G20..00 | 799 | Essential hypertension |
| G20..11 | 351 | High blood pressure |
| G200.00 | 15377 | Malignant essential hypertension |
| G200.00 | 15377 | Malignant essential hypertension |
| G201.00 | 1894 | Benign essential hypertension |
| G201.00 | 1894 | Benign essential hypertension |
| G202.00 | 4372 | Systolic hypertension |
| G202.00 | 4372 | Systolic hypertension |
| G20z.00 | 10818 | Essential hypertension NOS |
| G20z.00 | 10818 | Essential hypertension NOS |
| G20z.11 | 3712 | Hypertension NOS |
| G20z.11 | 3712 | Hypertension NOS |
| G21..00 | 16292 | Hypertensive heart disease |
| G21..00 | 16292 | Hypertensive heart disease |
| G210.00 | 50157 | Malignant hypertensive heart disease |
| G210.00 | 50157 | Malignant hypertensive heart disease |
| G210000 | 95334 | Malignant hypertensive heart disease without CCF |
| G210000 | 95334 | Malignant hypertensive heart disease without CCF |
| G210100 | 72668 | Malignant hypertensive heart disease with CCF |
| G210100 | 72668 | Malignant hypertensive heart disease with CCF |
| G210z00 | 103046 | Malignant hypertensive heart disease NOS |
| G210z00 | 103046 | Malignant hypertensive heart disease NOS |
| G211.00 | 52427 | Benign hypertensive heart disease |
| G211.00 | 52427 | Benign hypertensive heart disease |
| G211000 | 61660 | Benign hypertensive heart disease without CCF |
| G211000 | 61660 | Benign hypertensive heart disease without CCF |
| G211100 | 52127 | Benign hypertensive heart disease with CCF |
| G211100 | 52127 | Benign hypertensive heart disease with CCF |
| G211z00 | 105938 | Benign hypertensive heart disease NOS |
| G21z.00 | 31464 | Hypertensive heart disease NOS |
| G21z.00 | 31464 | Hypertensive heart disease NOS |
| G21z000 | 61166 | Hypertensive heart disease NOS without CCF |
| G21z000 | 61166 | Hypertensive heart disease NOS without CCF |
| G21z011 | 8857 | Cardiomegaly - hypertensive |
| G21z011 | 8857 | Cardiomegaly - hypertensive |
| G21z100 | 62718 | Hypertensive heart disease NOS with CCF |
| G21z100 | 62718 | Hypertensive heart disease NOS with CCF |
| G21zz00 | 16173 | Hypertensive heart disease NOS |
| G21zz00 | 16173 | Hypertensive heart disease NOS |
| G22..00 | 4668 | Hypertensive renal disease |
| G22..00 | 4668 | Hypertensive renal disease |
| G220.00 | 39649 | Malignant hypertensive renal disease |
| G220.00 | 39649 | Malignant hypertensive renal disease |
| G221.00 | 43935 | Benign hypertensive renal disease |
| G221.00 | 43935 | Benign hypertensive renal disease |
| G222.00 | 32423 | Hypertensive renal disease with renal failure |
| G222.00 | 32423 | Hypertensive renal disease with renal failure |
| G22z.00 | 15106 | Hypertensive renal disease NOS |
| G22z.00 | 15106 | Hypertensive renal disease NOS |
| G22z.11 | 29310 | Renal hypertension |
| G22z.11 | 29310 | Renal hypertension |
| G23..00 | 63466 | Hypertensive heart and renal disease |
| G23..00 | 63466 | Hypertensive heart and renal disease |
| G230.00 | 67232 | Malignant hypertensive heart and renal disease |
| G230.00 | 67232 | Malignant hypertensive heart and renal disease |
| G231.00 | 63000 | Benign hypertensive heart and renal disease |
| G231.00 | 63000 | Benign hypertensive heart and renal disease |
| G232.00 | 21837 | Hypertensive heart&renal dis wth (congestive) heart failure |
| G232.00 | 21837 | Hypertensive heart&renal dis wth (congestive) heart failure |
| G233.00 | 28684 | Hypertensive heart and renal disease with renal failure |
| G233.00 | 28684 | Hypertensive heart and renal disease with renal failure |
| G234.00 | 57987 | Hyperten heart&renal dis+both(congestv)heart and renal fail |
| G234.00 | 57987 | Hyperten heart&renal dis+both(congestv)heart and renal fail |
| G23z.00 | 68659 | Hypertensive heart and renal disease NOS |
| G23z.00 | 68659 | Hypertensive heart and renal disease NOS |
| G24..00 | 7329 | Secondary hypertension |
| G24..00 | 7329 | Secondary hypertension |
| G240.00 | 31755 | Secondary malignant hypertension |
| G240.00 | 31755 | Secondary malignant hypertension |
| G240000 | 59383 | Secondary malignant renovascular hypertension |
| G240000 | 59383 | Secondary malignant renovascular hypertension |
| G240z00 | 73293 | Secondary malignant hypertension NOS |
| G240z00 | 73293 | Secondary malignant hypertension NOS |
| G241.00 | 57288 | Secondary benign hypertension |
| G241.00 | 57288 | Secondary benign hypertension |
| G241000 | 25371 | Secondary benign renovascular hypertension |
| G241000 | 25371 | Secondary benign renovascular hypertension |
| G241z00 | 51635 | Secondary benign hypertension NOS |
| G241z00 | 51635 | Secondary benign hypertension NOS |
| G244.00 | 34744 | Hypertension secondary to endocrine disorders |
| G244.00 | 34744 | Hypertension secondary to endocrine disorders |
| G24z.00 | 16059 | Secondary hypertension NOS |
| G24z.00 | 16059 | Secondary hypertension NOS |
| G24z000 | 31387 | Secondary renovascular hypertension NOS |
| G24z000 | 31387 | Secondary renovascular hypertension NOS |
| G24zz00 | 42229 | Secondary hypertension NOS |
| G24zz00 | 42229 | Secondary hypertension NOS |
| G2y..00 | 18765 | Other specified hypertensive disease |
| G2y..00 | 18765 | Other specified hypertensive disease |
| G2z..00 | 7057 | Hypertensive disease NOS |
| G2z..00 | 7057 | Hypertensive disease NOS |
| G672.00 | 3979 | Hypertensive encephalopathy |
| G672.11 | 31816 | Hypertensive crisis |
| Gyu2000 | 102458 | [X]Other secondary hypertension |
| Gyu2000 | 102458 | [X]Other secondary hypertension |
| Gyu2100 | 97533 | [X]Hypertension secondary to other renal disorders |
| Gyu2100 | 97533 | [X]Hypertension secondary to other renal disorders |
| L120.00 | 12604 | Benign essential hypertension in pregnancy/childbirth/puerp |
| L120.00 | 12604 | Benign essential hypertension in pregnancy/childbirth/puerp |
| L120000 | 37344 | Benign essential hypertension in preg/childb/puerp unspec |
| L120000 | 37344 | Benign essential hypertension in preg/childb/puerp unspec |
| L120100 | 44912 | Benign essential hypertension in preg/childb/puerp - deliv |
| L120100 | 44912 | Benign essential hypertension in preg/childb/puerp - deliv |
| L120300 | 61408 | Benign essential hypertension in preg/childb/puerp-not deliv |
| L120300 | 61408 | Benign essential hypertension in preg/childb/puerp-not deliv |
| L120400 | 73633 | Benign essential hypertension in preg/childb/puerp +p/n comp |
| L120400 | 73633 | Benign essential hypertension in preg/childb/puerp +p/n comp |
| L120z00 | 34136 | Benign essential hypertension in preg/childb/puerp NOS |
| L120z00 | 34136 | Benign essential hypertension in preg/childb/puerp NOS |
| L121.00 | 93143 | Renal hypertension in pregnancy/childbirth/puerperium |
| L121.00 | 93143 | Renal hypertension in pregnancy/childbirth/puerperium |
| L121000 | 64127 | Renal hypertension in pregnancy/childbirth/puerp unspecified |
| L121000 | 64127 | Renal hypertension in pregnancy/childbirth/puerp unspecified |
| L121100 | 71717 | Renal hypertension in pregnancy/childbirth/puerp - delivered |
| L121100 | 71717 | Renal hypertension in pregnancy/childbirth/puerp - delivered |
| L121200 | 108840 | Renal hypertension in preg/childb/puerp -deliv with p/n comp |
| L121300 | 97349 | Renal hypertension in preg/childbirth/puerp - not delivered |
| L121300 | 97349 | Renal hypertension in preg/childbirth/puerp - not delivered |
| L121z00 | 94718 | Renal hypertension in pregnancy/childbirth/puerperium NOS |
| L121z00 | 94718 | Renal hypertension in pregnancy/childbirth/puerperium NOS |
| L122.00 | 66567 | Other pre-existing hypertension in preg/childbirth/puerp |
| L122.00 | 66567 | Other pre-existing hypertension in preg/childbirth/puerp |
| L122000 | 73586 | Other pre-existing hypertension in preg/childb/puerp unspec |
| L122000 | 73586 | Other pre-existing hypertension in preg/childb/puerp unspec |
| L122100 | 72030 | Other pre-existing hypertension in preg/childb/puerp - deliv |
| L122100 | 72030 | Other pre-existing hypertension in preg/childb/puerp - deliv |
| L122300 | 96743 | Other pre-exist hypertension in preg/childb/puerp-not deliv |
| L122300 | 96743 | Other pre-exist hypertension in preg/childb/puerp-not deliv |
| L122400 | 113968 | Other pre-exist hypertension in preg/childb/puerp + p/n comp |
| L122z00 | 62432 | Other pre-existing hypertension in preg/childb/puerp NOS |
| L122z00 | 62432 | Other pre-existing hypertension in preg/childb/puerp NOS |
| L128.00 | 44549 | Pre-exist hypertension compl preg childbirth and puerperium |
| L128.00 | 44549 | Pre-exist hypertension compl preg childbirth and puerperium |
| L128200 | 52621 | Pre-exist 2ndry hypertens comp preg childbth and puerperium |
| L128200 | 52621 | Pre-exist 2ndry hypertens comp preg childbth and puerperium |

Chronic Kidney Disease

| medcode | description |
| --- | --- |
| 10081 | Chronic uraemia |
| 10647 | Nephritis - chronic |
| 10809 | Chronic membranous glomerulonephritis |
| 11553 | Kidney transplant failure and rejection |
| 11875 | Nephropathy - chronic |
| 12479 | Chronic kidney disease stage 4 |
| 12566 | Chronic kidney disease stage 3 |
| 12585 | Chronic kidney disease stage 5 |
| 15097 | Chronic glomerulonephritis NOS |
| 17253 | Renal transplant planned |
| 17365 | Nephrotic syndrome; diffuse crescentic glomerulonephritis |
| 1803 | Nephrotic syndrome with membranous glomerulonephritis |
| 18774 | Renal transplant with complication; without blame |
| 19316 | Nephrotic syndrome; diffuse membranous glomerulonephritis |
| 21158 | Chronic pyelitis |
| 21947 | Nephrotic syn difus mesangial prolifertiv glomerulonephritis |
| 21989 | Nephrotic syn;diffuse mesangiocapillary glomerulonephritis |
| 22205 | Lupus nephritis |
| 22852 | Nephrotic syndrome; focal and segmental glomerular lesions |
| 23913 | Nephrotic syndrome; minor glomerular abnormality |
| 2471 | Nephrotic syndrome in diabetes mellitus |
| 25055 | Chronic pyonephrosis |
| 26862 | Exploration of renal transplant |
| 27427 | Nephrotic syndrome NOS |
| 2939 | Calculous pyelonephritis |
| 29634 | Nephrotic syndrome with minimal change glomerulonephritis |
| 2999 | Nephrotic syndrome |
| 34998 | Chronic proliferative glomerulonephritis |
| 35360 | Nonobstructive reflux-associated chronic pyelonephritis |
| 40349 | Lipoid nephrosis |
| 45499 | Kimmelstiel - Wilson disease |
| 4654 | Chronic pyelonephritis |
| 4669 | Chronic focal glomerulonephritis |
| 47672 | Nephrotic syndrome in systemic lupus erythematosus |
| 47922 | Nephrotic syndrome in amyloidosis |
| 48111 | Chronic pyelonephritis NOS |
| 48855 | Chronic obstructive pyelonephritis |
| 50472 | Nephrotic syn;difus endocapilary proliftv glomerulonephritis |
| 512 | Chronic renal failure |
| 53852 | End stage renal failure |
| 54990 | Kidney transplant with complication; without blame |
| 56987 | Nephrotic syndrome; dense deposit disease |
| 57568 | Chronic pyelonephritis with medullary necrosis |
| 57926 | Steroid sensitive nephrotic syndrome |
| 58750 | Nephrotic syndrome in polyarteritis nodosa |
| 5911 | [V]Kidney transplanted |
| 60960 | Other chronic glomerulonephritis |
| 61494 | Chronic membranoproliferative glomerulonephritis |
| 63615 | Other chronic glomerulonephritis NOS |
| 63786 | Congenital nephrotic syndrome |
| 65064 | Chronic rapidly progressive glomerulonephritis |
| 65400 | Chronic diffuse glomerulonephritis |
| 6712 | End stage renal failure |
| 72303 | Finnish nephrosis syndrome |
| 7804 | Chronic glomerulonephritis |
| 8330 | End-stage renal disease |
| 94373 | Nephrotic syndrome with other pathological kidney lesions |
| 94793 | Chronic kidney disease stage 3 with proteinuria |
| 94965 | Chronic kidney disease stage 3A |
| 95122 | Chronic kidney disease stage 4 with proteinuria |
| 95123 | Chronic kidney disease stage 3 without proteinuria |
| 95175 | Chronic kidney disease stage 3A without proteinuria |
| 95177 | Chronic kidney disease stage 3B without proteinuria |
| 95178 | Chronic kidney disease stage 3B with proteinuria |
| 95179 | Chronic kidney disease stage 3B |
| 95405 | Chronic kidney disease stage 5 without proteinuria |
| 95406 | Chronic kidney disease stage 4 without proteinuria |
| 95408 | Chronic kidney disease stage 3A with proteinuria |
| 95508 | Chronic kidney disease stage 5 with proteinuria |
| 97758 | Chronic glomerulonephritis + diseases EC |
| 9840 | Nephrotic syndrome with proliferative glomerulonephritis |
| 99201 | Nephrotic syndrome in malaria |
| 99631 | Chronic pyelonephritis without medullary necrosis |
| 99644 | Nephrotic syndrome+membranoproliferative glomerulonephritis |

Osteoporosis

| readcode | medcode | readterm |
| --- | --- | --- |
| 585O.00 | 51891 | Quantitative ultrasound scan of heel - result osteoporotic |
| 58E4.00 | 13987 | Forearm DXA scan result osteoporotic |
| 58E8.00 | 37972 | Heel DXA scan T score |
| 58EA.00 | 46510 | Heel DXA scan result osteoporotic |
| 58EA.00 | 46510 | Heel DXA scan result osteoporotic |
| 58EE.00 | 40904 | Hip DXA scan T score |
| 58EG.00 | 42354 | Hip DXA scan result osteoporotic |
| 58EG.00 | 42354 | Hip DXA scan result osteoporotic |
| 58EK.00 | 11581 | Lumbar spine DXA scan T score |
| 58EM.00 | 39217 | Lumbar DXA scan result osteoporotic |
| 58EM.00 | 39217 | Lumbar DXA scan result osteoporotic |
| 58ES.00 | 97266 | Femoral neck DEXA scan T score |
| 58EV.00 | 96342 | Femoral neck DEXA scan result osteoporotic |
| N330.00 | 277 | Osteoporosis |
| N330.00 | 277 | Osteoporosis |
| N330000 | 14967 | Osteoporosis, unspecified |
| N330000 | 14967 | Osteoporosis, unspecified |
| N330100 | 16307 | Senile osteoporosis |
| N330100 | 16307 | Senile osteoporosis |
| N330200 | 9700 | Postmenopausal osteoporosis |
| N330200 | 9700 | Postmenopausal osteoporosis |
| N330300 | 40428 | Idiopathic osteoporosis |
| N330300 | 40428 | Idiopathic osteoporosis |
| N330400 | 62702 | Dissuse osteoporosis |
| N330400 | 62702 | Dissuse osteoporosis |
| N330500 | 24093 | Drug-induced osteoporosis |
| N330500 | 24093 | Drug-induced osteoporosis |
| N330600 | 70349 | Postoophorectomy osteoporosis |
| N330600 | 70349 | Postoophorectomy osteoporosis |
| N330700 | 93655 | Postsurgical malabsorption osteoporosis |
| N330700 | 93655 | Postsurgical malabsorption osteoporosis |
| N330800 | 54232 | Localized osteoporosis - Lequesne |
| N330800 | 54232 | Localized osteoporosis - Lequesne |
| N330900 | 60433 | Osteoporosis in multiple myelomatosis |
| N330900 | 60433 | Osteoporosis in multiple myelomatosis |
| N330A00 | 31580 | Osteoporosis in endocrine disorders |
| N330A00 | 31580 | Osteoporosis in endocrine disorders |
| N330B00 | 3346 | Vertebral osteoporosis |
| N330B00 | 3346 | Vertebral osteoporosis |
| N330C00 | 16857 | Osteoporosis localized to spine |
| N330C00 | 16857 | Osteoporosis localized to spine |
| N330D00 | 25650 | Osteoporosis due to corticosteroids |
| N330D00 | 25650 | Osteoporosis due to corticosteroids |
| N330z00 | 34798 | Osteoporosis NOS |
| N330z00 | 34798 | Osteoporosis NOS |
| N331200 | 39334 | Postoophorectomy osteoporosis with pathological fracture |
| N331200 | 39334 | Postoophorectomy osteoporosis with pathological fracture |
| N331300 | 33526 | Osteoporosis of disuse with pathological fracture |
| N331300 | 33526 | Osteoporosis of disuse with pathological fracture |
| N331400 | 68019 | Postsurgical malabsorption osteoporosis with path fracture |
| N331500 | 46894 | Drug-induced osteoporosis with pathological fracture |
| N331600 | 27597 | Idiopathic osteoporosis with pathological fracture |
| N331600 | 27597 | Idiopathic osteoporosis with pathological fracture |
| N331800 | 17377 | Osteoporosis + pathological fracture lumbar vertebrae |
| N331900 | 12673 | Osteoporosis + pathological fracture thoracic vertebrae |
| N331A00 | 48772 | Osteoporosis + pathological fracture cervical vertebrae |
| N331B00 | 38395 | Postmenopausal osteoporosis with pathological fracture |
| N331M00 | 11503 | Fragility fracture due to unspecified osteoporosis |
| N331N00 | 93497 | Fragility fracture |
| NyuB000 | 57301 | [X]Other osteoporosis with pathological fracture |
| NyuB000 | 57301 | [X]Other osteoporosis with pathological fracture |
| NyuB100 | 41755 | [X]Other osteoporosis |
| NyuB100 | 41755 | [X]Other osteoporosis |
| NyuB200 | 102730 | [X]Osteoporosis in other disorders classified elsewhere |
| NyuB800 | 18825 | [X]Unspecified osteoporosis with pathological fracture |
| NyuB800 | 18825 | [X]Unspecified osteoporosis with pathological fracture |

Dementia

| medcode | readcode | readterm |
| --- | --- | --- |
| 1917 | F110.00 | Alzheimer's disease |
| 7664 | Eu00.00 | [X]Dementia in Alzheimer's disease |
| 8195 | Eu00z11 | [X]Alzheimer's dementia unspec |
| 11379 | Eu00112 | [X]Senile dementia,Alzheimer's type |
| 16797 | F110000 | Alzheimer's disease with early onset |
| 25704 | Eu00011 | [X]Presenile dementia,Alzheimer's type |
| 29386 | Eu00z00 | [X]Dementia in Alzheimer's disease, unspecified |
| 32057 | F110100 | Alzheimer's disease with late onset |
| 38678 | Eu00100 | [X]Dementia in Alzheimer's disease with late onset |
| 43346 | Eu00113 | [X]Primary degen dementia of Alzheimer's type, senile onset |
| 46762 | Eu00111 | [X]Alzheimer's disease type 1 |
| 49263 | Eu00000 | [X]Dementia in Alzheimer's disease with early onset |
| 59122 | Fyu3000 | [X]Other Alzheimer's disease |
| 60059 | Eu00012 | [X]Primary degen dementia, Alzheimer's type, presenile onset |
| 61528 | Eu00013 | [X]Alzheimer's disease type 2 |
| 5095 | F21y200 | Binswanger's disease |
| 6578 | Eu01.00 | [X]Vascular dementia |
| 8634 | E004.11 | Multi infarct dementia |
| 8934 | Eu01200 | [X]Subcortical vascular dementia |
| 9565 | Eu01.11 | [X]Arteriosclerotic dementia |
| 11175 | Eu01100 | [X]Multi-infarct dementia |
| 19393 | Eu01z00 | [X]Vascular dementia, unspecified |
| 19477 | E004.00 | Arteriosclerotic dementia |
| 31016 | Eu01300 | [X]Mixed cortical and subcortical vascular dementia |
| 42279 | E004z00 | Arteriosclerotic dementia NOS |
| 43089 | E004000 | Uncomplicated arteriosclerotic dementia |
| 43292 | E004300 | Arteriosclerotic dementia with depression |
| 46488 | Eu01000 | [X]Vascular dementia of acute onset |
| 55313 | Eu01y00 | [X]Other vascular dementia |
| 55467 | E004200 | Arteriosclerotic dementia with paranoia |
| 56912 | E004100 | Arteriosclerotic dementia with delirium |
| 68194 | F21y211 | Binswanger's encephalopathy |
| 7572 | F116.00 | Lewy body disease |
| 26270 | Eu02500 | [X]Lewy body dementia |
| 11136 | F111.00 | Pick's disease |
| 28402 | Eu02000 | [X]Dementia in Pick's disease |
| 104534 | F118.00 | Frontotemporal degeneration |
| 3591 | F134.00 | Huntington's chorea |
| 37014 | Eu02200 | [X]Dementia in Huntington's disease |
| 9509 | Eu02300 | [X]Dementia in Parkinson's disease |
| 4500 | E011000 | Korsakoff's alcoholic psychosis |
| 4501 | C251.11 | Wernicke's encephalopathy |
| 11106 | E011100 | Korsakov's alcoholic psychosis with peripheral neuritis |
| 11107 | C253.00 | Wernicke's encephalopathy |
| 11670 | Eu10611 | [x]Korsakoff's psychosis, alcohol induced |
| 18636 | E011200 | Wernicke-Korsakoff syndrome |
| 23835 | E040.11 | Korsakoff's non-alcoholic psychosis |
| 26323 | Eu10711 | [X]Alcoholic dementia NOS |
| 27342 | E012.11 | Alcoholic dementia NOS |
| 37691 | Eu10712 | [X]Chronic alcoholic brain syndrome |
| 37946 | E012000 | Chronic alcoholic brain syndrome |
| 54505 | E012.00 | Other alcoholic dementia |
| 57993 | Eu03.11 | [X] Korsakoff's psychosis, nonalcoholic |
| 62132 | E02y100 | Drug-induced dementia |
| 38286 | A411.00 | Jakob-Creutzfeldt disease |
| 48531 | F11x700 | Cerebral degeneration due to Jakob - Creutzfeldt disease |
| 54106 | Eu02100 | [X]Dementia in Creutzfeldt-Jakob disease |
| 70709 | 4L49.00 | Prion protein markers for Creutzfeldt-Jakob disease |
| 109288 | A411000 | Sporadic Creutzfeldt-Jakob disease |
| 41185 | Eu02400 | [X]Dementia in human immunodef virus [HIV] disease |
| 12621 | Eu02.00 | [X]Dementia in other diseases classified elsewhere |
| 25386 | E041.00 | Dementia in conditions EC |
| 64267 | Eu02y00 | [X]Dementia in other specified diseases classif elsewhere |
| 2882 | E00z.00 | Senile or presenile psychoses NOS |
| 15249 | E00y.00 | Other senile and presenile organic psychoses |
| 27935 | Eu02z15 | [X] Senile psychosis NOS |
| 29512 | F112.00 | Senile degeneration of brain |
| 33707 | E00..00 | Senile and presenile organic psychotic conditions |
| 47619 | Eu02z12 | [X] Presenile psychosis nos |
| 47873 | Eu05z11 | [X]Organic brain syndrome NOS |
| 51494 | E00y.11 | Presbyophrenic psychosis |
| 1350 | E00..12 | Senile/presenile dementia |
| 1916 | E00..11 | Senile dementia |
| 4357 | Eu02z14 | [X] Senile dementia NOS |
| 4693 | Eu02z00 | [X] Unspecified dementia |
| 7323 | E000.00 | Uncomplicated senile dementia |
| 15165 | E001.00 | Presenile dementia |
| 18386 | E002000 | Senile dementia with paranoia |
| 21887 | E002100 | Senile dementia with depression |
| 27677 | E001300 | Presenile dementia with depression |
| 27759 | Eu02z16 | [X] Senile dementia, depressed or paranoid type |
| 30032 | E001200 | Presenile dementia with paranoia |
| 34944 | Eu02z13 | [X] Primary degenerative dementia NOS |
| 37015 | E003.00 | Senile dementia with delirium |
| 38438 | E001z00 | Presenile dementia NOS |
| 41089 | E002z00 | Senile dementia with depressive or paranoid features NOS |
| 42602 | E001000 | Uncomplicated presenile dementia |
| 44674 | E002.00 | Senile dementia with depressive or paranoid features |
| 48501 | Eu02z11 | [X] Presenile dementia NOS |
| 49513 | E001100 | Presenile dementia with delirium |
| 53446 | Eu04100 | [X]Delirium superimposed on dementia |
| 55222 | ZS7C500 | Language disorder of dementia |
| 55838 | Eu01111 | [X]Predominantly cortical dementia |
| 30706 | Eu00200 | [X]Dementia in Alzheimer's dis, atypical or mixed type |

Serious Mental Illness

| readcode | medcode | readterm |
| --- | --- | --- |
| E100.00 | 32222 | Simple schizophrenia |
| E100.11 | 73295 | Schizophrenia simplex |
| E100000 | 15733 | Unspecified schizophrenia |
| E100100 | 23616 | Subchronic schizophrenia |
| E100200 | 3984 | Chronic schizophrenic |
| E100300 | 57666 | Acute exacerbation of subchronic schizophrenia |
| E100400 | 44498 | Acute exacerbation of chronic schizophrenia |
| E100500 | 58687 | Schizophrenia in remission |
| E100z00 | 53625 | Simple schizophrenia NOS |
| E101.00 | 30619 | Hebephrenic schizophrenia |
| E101000 | 66506 | Unspecified hebephrenic schizophrenia |
| E101400 | 97919 | Acute exacerbation of chronic hebephrenic schizophrenia |
| E101500 | 67768 | Hebephrenic schizophrenia in remission |
| E101z00 | 48054 | Hebephrenic schizophrenia NOS |
| E102.00 | 25546 | Catatonic schizophrenia |
| E102000 | 58716 | Unspecified catatonic schizophrenia |
| E102100 | 99199 | Subchronic catatonic schizophrenia |
| E102500 | 102427 | Catatonic schizophrenia in remission |
| E102z00 | 63867 | Catatonic schizophrenia NOS |
| E103.00 | 1494 | Paranoid schizophrenia |
| E103000 | 33383 | Unspecified paranoid schizophrenia |
| E103200 | 31362 | Chronic paranoid schizophrenia |
| E103300 | 51322 | Acute exacerbation of subchronic paranoid schizophrenia |
| E103400 | 53032 | Acute exacerbation of chronic paranoid schizophrenia |
| E103500 | 36172 | Paranoid schizophrenia in remission |
| E103z00 | 9281 | Paranoid schizophrenia NOS |
| E104.00 | 576 | Acute schizophrenic episode |
| E105.00 | 66410 | Latent schizophrenia |
| E105000 | 102311 | Unspecified latent schizophrenia |
| E105200 | 94299 | Chronic latent schizophrenia |
| E105500 | 96883 | Latent schizophrenia in remission |
| E105z00 | 102446 | Latent schizophrenia NOS |
| E106.00 | 38063 | Residual schizophrenia |
| E107.00 | 2117 | Schizo-affective schizophrenia |
| E107.11 | 99000 | Cyclic schizophrenia |
| E107000 | 58862 | Unspecified schizo-affective schizophrenia |
| E107100 | 61098 | Subchronic schizo-affective schizophrenia |
| E107200 | 43800 | Chronic schizo-affective schizophrenia |
| E107300 | 58866 | Acute exacerbation subchronic schizo-affective schizophrenia |
| E107400 | 63478 | Acute exacerbation of chronic schizo-affective schizophrenia |
| E107500 | 56438 | Schizo-affective schizophrenia in remission |
| E107z00 | 10575 | Schizo-affective schizophrenia NOS |
| E10y.00 | 39062 | Other schizophrenia |
| E10y.11 | 92994 | Cenesthopathic schizophrenia |
| E10y000 | 33338 | Atypical schizophrenia |
| E10y100 | 99070 | Coenesthopathic schizophrenia |
| E10yz00 | 49761 | Other schizophrenia NOS |
| E10z.00 | 8407 | Schizophrenia NOS |
| E11..00 | 14656 | Affective psychoses |
| E11..12 | 2560 | Depressive psychoses |
| E110.00 | 37070 | Manic disorder, single episode |
| E110.11 | 18909 | Hypomanic psychoses |
| E110000 | 20110 | Single manic episode, unspecified |
| E110100 | 14728 | Single manic episode, mild |
| E110200 | 24640 | Single manic episode, moderate |
| E110300 | 43093 | Single manic episode, severe without mention of psychosis |
| E110400 | 50218 | Single manic episode, severe, with psychosis |
| E110600 | 70000 | Single manic episode in full remission |
| E110z00 | 36611 | Manic disorder, single episode NOS |
| E111.00 | 26227 | Recurrent manic episodes |
| E111000 | 19967 | Recurrent manic episodes, unspecified |
| E111100 | 46425 | Recurrent manic episodes, mild |
| E111200 | 27739 | Recurrent manic episodes, moderate |
| E111300 | 65811 | Recurrent manic episodes, severe without mention psychosis |
| E111400 | 32295 | Recurrent manic episodes, severe, with psychosis |
| E111500 | 58863 | Recurrent manic episodes, partial or unspecified remission |
| E111600 | 37178 | Recurrent manic episodes, in full remission |
| E111z00 | 46415 | Recurrent manic episode NOS |
| E112400 | 32159 | Single major depressive episode, severe, with psychosis |
| E113400 | 24171 | Recurrent major depressive episodes, severe, with psychosis |
| E114.00 | 3702 | Bipolar affective disorder, currently manic |
| E114.11 | 17385 | Manic-depressive - now manic |
| E114000 | 35738 | Bipolar affective disorder, currently manic, unspecified |
| E114100 | 36126 | Bipolar affective disorder, currently manic, mild |
| E114200 | 46434 | Bipolar affective disorder, currently manic, moderate |
| E114300 | 16347 | Bipolar affect disord, currently manic, severe, no psychosis |
| E114400 | 55829 | Bipolar affect disord, currently manic,severe with psychosis |
| E114500 | 59011 | Bipolar affect disord,currently manic, part/unspec remission |
| E114600 | 63784 | Bipolar affective disorder, currently manic, full remission |
| E114z00 | 57605 | Bipolar affective disorder, currently manic, NOS |
| E115.00 | 4677 | Bipolar affective disorder, currently depressed |
| E115.11 | 12831 | Manic-depressive - now depressed |
| E115000 | 15923 | Bipolar affective disorder, currently depressed, unspecified |
| E115100 | 35734 | Bipolar affective disorder, currently depressed, mild |
| E115200 | 27890 | Bipolar affective disorder, currently depressed, moderate |
| E115300 | 35607 | Bipolar affect disord, now depressed, severe, no psychosis |
| E115400 | 63701 | Bipolar affect disord, now depressed, severe with psychosis |
| E115500 | 72026 | Bipolar affect disord, now depressed, part/unspec remission |
| E115600 | 57465 | Bipolar affective disorder, now depressed, in full remission |
| E115z00 | 37296 | Bipolar affective disorder, currently depressed, NOS |
| E116.00 | 31316 | Mixed bipolar affective disorder |
| E116000 | 31535 | Mixed bipolar affective disorder, unspecified |
| E116100 | 24689 | Mixed bipolar affective disorder, mild |
| E116200 | 63150 | Mixed bipolar affective disorder, moderate |
| E116300 | 63284 | Mixed bipolar affective disorder, severe, without psychosis |
| E116400 | 54195 | Mixed bipolar affective disorder, severe, with psychosis |
| E116500 | 63651 | Mixed bipolar affective disorder, partial/unspec remission |
| E116600 | 55064 | Mixed bipolar affective disorder, in full remission |
| E116z00 | 63583 | Mixed bipolar affective disorder, NOS |
| E117.00 | 14784 | Unspecified bipolar affective disorder |
| E117000 | 49763 | Unspecified bipolar affective disorder, unspecified |
| E117100 | 63698 | Unspecified bipolar affective disorder, mild |
| E117200 | 68647 | Unspecified bipolar affective disorder, moderate |
| E117300 | 73423 | Unspecified bipolar affective disorder, severe, no psychosis |
| E117400 | 68326 | Unspecified bipolar affective disorder,severe with psychosis |
| E117500 | 70721 | Unspecified bipolar affect disord, partial/unspec remission |
| E117600 | 24230 | Unspecified bipolar affective disorder, in full remission |
| E117z00 | 27986 | Unspecified bipolar affective disorder, NOS |
| E11y.00 | 60178 | Other and unspecified manic-depressive psychoses |
| E11y000 | 11596 | Unspecified manic-depressive psychoses |
| E11y100 | 70925 | Atypical manic disorder |
| E11y300 | 70399 | Other mixed manic-depressive psychoses |
| E11yz00 | 33426 | Other and unspecified manic-depressive psychoses NOS |
| E11z.00 | 41992 | Other and unspecified affective psychoses |
| E11z000 | 54607 | Unspecified affective psychoses NOS |
| E11zz00 | 33425 | Other affective psychosis NOS |
| E120.00 | 14743 | Simple paranoid state |
| E121.00 | 3890 | Chronic paranoid psychosis |
| E122.00 | 14971 | Paraphrenia |
| E123.00 | 62680 | Shared paranoid disorder |
| E123.11 | 50868 | Folie a deux |
| E12y.00 | 31589 | Other paranoid states |
| E12y000 | 66766 | Paranoia querulans |
| E12yz00 | 31455 | Other paranoid states NOS |
| E12z.00 | 12771 | Paranoid psychosis NOS |
| E13..00 | 31984 | Other nonorganic psychoses |
| E13..11 | 20228 | Reactive psychoses |
| E130.00 | 8478 | Reactive depressive psychosis |
| E130.11 | 17770 | Psychotic reactive depression |
| E131.00 | 29937 | Acute hysterical psychosis |
| E132.00 | 7332 | Reactive confusion |
| E133.00 | 15053 | Acute paranoid reaction |
| E133.11 | 68058 | Bouffee delirante |
| E134.00 | 24345 | Psychogenic paranoid psychosis |
| E13y.00 | 16333 | Other reactive psychoses |
| E13y000 | 22117 | Psychogenic stupor |
| E13y100 | 23538 | Brief reactive psychosis |
| E13yz00 | 26119 | Other reactive psychoses NOS |
| E13z.00 | 14965 | Nonorganic psychosis NOS |
| E13z.11 | 3636 | Psychotic episode NOS |
| E1z..00 | 22188 | Non-organic psychosis NOS |
| E212200 | 61969 | Schizotypal personality |
| Eu20.00 | 34236 | [X]Schizophrenia |
| Eu20000 | 16764 | [X]Paranoid schizophrenia |
| Eu20011 | 50060 | [X]Paraphrenic schizophrenia |
| Eu20100 | 43405 | [X]Hebephrenic schizophrenia |
| Eu20111 | 53985 | [X]Disorganised schizophrenia |
| Eu20200 | 61501 | [X]Catatonic schizophrenia |
| Eu20211 | 20572 | [X]Catatonic stupor |
| Eu20212 | 64533 | [X]Schizophrenic catalepsy |
| Eu20213 | 35877 | [X]Schizophrenic catatonia |
| Eu20214 | 31493 | [X]Schizophrenic flexibilatis cerea |
| Eu20300 | 60013 | [X]Undifferentiated schizophrenia |
| Eu20311 | 91547 | [X]Atypical schizophrenia |
| Eu20400 | 20785 | [X]Post-schizophrenic depression |
| Eu20500 | 64264 | [X]Residual schizophrenia |
| Eu20511 | 24107 | [X]Chronic undifferentiated schizophrenia |
| Eu20600 | 35848 | [X]Simple schizophrenia |
| Eu20y00 | 49420 | [X]Other schizophrenia |
| Eu20y12 | 94001 | [X]Schizophreniform disord NOS |
| Eu20y13 | 18053 | [X]Schizophrenifrm psychos NOS |
| Eu20z00 | 34966 | [X]Schizophrenia, unspecified |
| Eu21.00 | 39316 | [X]Schizotypal disorder |
| Eu21.11 | 91511 | [X]Latent schizophrenic reaction |
| Eu21.12 | 54387 | [X]Borderline schizophrenia |
| Eu21.13 | 64993 | [X]Latent schizophrenia |
| Eu21.14 | 62449 | [X]Prepsychotic schizophrenia |
| Eu21.15 | 40386 | [X]Prodromal schizophrenia |
| Eu21.16 | 49852 | [X]Pseudoneurotic schizophrenia |
| Eu21.17 | 71250 | [X]Pseudopsychopathic schizophrenia |
| Eu21.18 | 26859 | [X]Schizotypal personality disorder |
| Eu22.00 | 28562 | [X]Persistent delusional disorders |
| Eu22000 | 34389 | [X]Delusional disorder |
| Eu22011 | 2113 | [X]Paranoid psychosis |
| Eu22012 | 11172 | [X]Paranoid state |
| Eu22013 | 47947 | [X]Paraphrenia - late |
| Eu22014 | 65127 | [X]Sensitiver Beziehungswahn |
| Eu22015 | 4843 | [X]Paranoia |
| Eu22100 | 62405 | [X]Delusional misidentification syndrome |
| Eu22111 | 55221 | [X]Capgras syndrome |
| Eu22200 | 98821 | [X]Cotard syndrome |
| Eu22300 | 101720 | [X]Paranoid state in remission |
| Eu22y00 | 66077 | [X]Other persistent delusional disorders |
| Eu22y11 | 40981 | [X]Delusional dysmorphophobia |
| Eu22y12 | 50248 | [X]Involutional paranoid state |
| Eu22y13 | 55236 | [X]Paranoia querulans |
| Eu22z00 | 49223 | [X]Persistent delusional disorder, unspecified |
| Eu23.00 | 25019 | [X]Acute and transient psychotic disorders |
| Eu23000 | 36720 | [X]Acute polymorphic psychot disord without symp of schizoph |
| Eu23011 | 50023 | [X]Bouffee delirante |
| Eu23012 | 21455 | [X]Cycloid psychosis |
| Eu23100 | 21595 | [X]Acute polymorphic psychot disord with symp of schizophren |
| Eu23112 | 26143 | [X]Cycloid psychosis with symptoms of schizophrenia |
| Eu23200 | 11778 | [X]Acute schizophrenia-like psychotic disorder |
| Eu23211 | 59096 | [X]Brief schizophreniform disorder |
| Eu23212 | 70884 | [X]Brief schizophrenifrm psych |
| Eu23214 | 94604 | [X]Schizophrenic reaction |
| Eu23300 | 44307 | [X]Other acute predominantly delusional psychotic disorders |
| Eu23312 | 27770 | [X]Psychogenic paranoid psychosis |
| Eu23y00 | 44503 | [X]Other acute and transient psychotic disorders |
| Eu23z00 | 34168 | [X]Acute and transient psychotic disorder, unspecified |
| Eu23z11 | 31707 | [X]Brief reactive psychosis NOS |
| Eu23z12 | 29651 | [X]Reactive psychosis |
| Eu24.00 | 51302 | [X]Induced delusional disorder |
| Eu24.12 | 47230 | [X]Induced paranoid disorder |
| Eu24.13 | 11973 | [X]Induced psychotic disorder |
| Eu25.00 | 9422 | [X]Schizoaffective disorders |
| Eu25000 | 33847 | [X]Schizoaffective disorder, manic type |
| Eu25011 | 16905 | [X]Schizoaffective psychosis, manic type |
| Eu25012 | 51903 | [X]Schizophreniform psychosis, manic type |
| Eu25100 | 11055 | [X]Schizoaffective disorder, depressive type |
| Eu25111 | 35274 | [X]Schizoaffective psychosis, depressive type |
| Eu25112 | 41022 | [X]Schizophreniform psychosis, depressive type |
| Eu25200 | 33693 | [X]Schizoaffective disorder, mixed type |
| Eu25212 | 37580 | [X]Mixed schizophrenic and affective psychosis |
| Eu25y00 | 58532 | [X]Other schizoaffective disorders |
| Eu25z00 | 37681 | [X]Schizoaffective disorder, unspecified |
| Eu25z11 | 33410 | [X]Schizoaffective psychosis NOS |
| Eu26.00 | 101987 | [X]Nonorganic psychosis in remission |
| Eu2y.00 | 30985 | [X]Other nonorganic psychotic disorders |
| Eu2y.11 | 31738 | [X]Chronic hallucinatory psychosis |
| Eu2z.00 | 11244 | [X]Unspecified nonorganic psychosis |
| Eu2z.11 | 694 | [X]Psychosis NOS |
| Eu30.00 | 12173 | [X]Manic episode |
| Eu30.11 | 9521 | [X]Bipolar disorder, single manic episode |
| Eu30000 | 2741 | [X]Hypomania |
| Eu30100 | 13024 | [X]Mania without psychotic symptoms |
| Eu30200 | 21065 | [X]Mania with psychotic symptoms |
| Eu30211 | 37102 | [X]Mania with mood-congruent psychotic symptoms |
| Eu30212 | 48632 | [X]Mania with mood-incongruent psychotic symptoms |
| Eu30y00 | 32088 | [X]Other manic episodes |
| Eu30z00 | 44513 | [X]Manic episode, unspecified |
| Eu30z11 | 4678 | [X]Mania NOS |
| Eu31.00 | 6874 | [X]Bipolar affective disorder |
| Eu31.11 | 1531 | [X]Manic-depressive illness |
| Eu31.12 | 6710 | [X]Manic-depressive psychosis |
| Eu31.13 | 66153 | [X]Manic-depressive reaction |
| Eu31000 | 16808 | [X]Bipolar affective disorder, current episode hypomanic |
| Eu31100 | 26299 | [X]Bipolar affect disorder cur epi manic wout psychotic symp |
| Eu31200 | 28277 | [X]Bipolar affect disorder cur epi manic with psychotic symp |
| Eu31300 | 16562 | [X]Bipolar affect disorder cur epi mild or moderate depressn |
| Eu31400 | 23713 | [X]Bipol aff disord, curr epis sev depress, no psychot symp |
| Eu31500 | 4732 | [X]Bipolar affect dis cur epi severe depres with psyc symp |
| Eu31600 | 44693 | [X]Bipolar affective disorder, current episode mixed |
| Eu31700 | 27584 | [X]Bipolar affective disorder, currently in remission |
| Eu31800 | 104065 | [X]Bipolar affective disorder type I |
| Eu31900 | 103915 | [X]Bipolar affective disorder type II |
| Eu31911 | 104051 | [X]Bipolar II disorder |
| Eu31y00 | 53840 | [X]Other bipolar affective disorders |
| Eu31y11 | 73924 | [X]Bipolar II disorder |
| Eu31y12 | 51032 | [X]Recurrent manic episodes |
| Eu31z00 | 33751 | [X]Bipolar affective disorder, unspecified |
| Eu32300 | 12099 | [X]Severe depressive episode with psychotic symptoms |
| Eu32311 | 24117 | [X]Single episode of major depression and psychotic symptoms |
| Eu32312 | 52678 | [X]Single episode of psychogenic depressive psychosis |
| Eu32313 | 24112 | [X]Single episode of psychotic depression |
| Eu32314 | 28863 | [X]Single episode of reactive depressive psychosis |
| Eu32800 | 98417 | [X]Major depression, severe with psychotic symptoms |
| Eu33213 | 29451 | [X]Manic-depress psychosis,depressd,no psychotic symptoms |
| Eu33300 | 47009 | [X]Recurrent depress disorder cur epi severe with psyc symp |
| Eu33311 | 23731 | [X]Endogenous depression with psychotic symptoms |
| Eu33312 | 28677 | [X]Manic-depress psychosis,depressed type+psychotic symptoms |
| Eu33313 | 32941 | [X]Recurr severe episodes/major depression+psychotic symptom |
| Eu33314 | 31757 | [X]Recurr severe episodes/psychogenic depressive psychosis |
| Eu33315 | 16861 | [X]Recurrent severe episodes of psychotic depression |
| Eu33316 | 37764 | [X]Recurrent severe episodes/reactive depressive psychosis |
| Eu3z.11 | 31633 | [X]Affective psychosis NOS |
| Eu44.14 | 28168 | [X]Hysterical psychosis |

Epilepsy

| readcode | medcode | readterm |
| --- | --- | --- |
| 1B1W.00 | 38919 | Transient epileptic amnesia |
| 2126000 | 8385 | Epilepsy resolved |
| 212J.00 | 12848 | Epilepsy resolved |
| 667..00 | 6983 | Epilepsy monitoring |
| 667A.00 | 20566 | Epilepsy treatment stopped |
| 667B.00 | 4602 | Nocturnal epilepsy |
| 667C.00 | 19550 | Epilepsy control good |
| 667D.00 | 13220 | Epilepsy control poor |
| 667E.00 | 19551 | Epilepsy care arrangement |
| 667F.00 | 11015 | Seizure free >12 months |
| 667G.00 | 26620 | Epilepsy restricts employment |
| 667H.00 | 50702 | Epilepsy prevents employment |
| 667J.00 | 40863 | Epilepsy impairs education |
| 667K.00 | 26619 | Epilepsy limits activities |
| 667L.00 | 19552 | Epilepsy does not limit activities |
| 667M.00 | 55706 | Epilepsy management plan given |
| 667N.00 | 22991 | Epilepsy severity |
| 667P.00 | 13219 | No seizures on treatment |
| 667Q.00 | 26618 | 1 to 12 seizures a year |
| 667R.00 | 13221 | 2 to 4 seizures a month |
| 667S.00 | 19549 | 1 to 7 seizures a week |
| 667T.00 | 18899 | Daily seizures |
| 667V.00 | 39160 | Many seizures a day |
| 667W.00 | 46603 | Emergency epilepsy treatment since last appointment |
| 667X.00 | 52632 | No epilepsy drug side effects |
| 8BIF.00 | 9326 | Epilepsy medication review |
| 8BL3.00 | 11752 | Patient on maximal tolerated anticonvulsant therapy |
| 9N0r.00 | 47117 | Seen in epilepsy clinic |
| 9N4V.00 | 35217 | DNA - Did not attend epilepsy clinic |
| Eu05212 | 31877 | [X]Schizophrenia-like psychosis in epilepsy |
| Eu05y11 | 6709 | [X]Epileptic psychosis NOS |
| Eu06013 | 48462 | [X]Limbic epilepsy personality |
| Eu80300 | 43679 | [X]Acquired aphasia with epilepsy [Landau - Kleffner] |
| F132100 | 37644 | Progressive myoclonic epilepsy |
| F25..00 | 573 | Epilepsy |
| F250.00 | 11186 | Generalised nonconvulsive epilepsy |
| F250000 | 2907 | Petit mal (minor) epilepsy |
| F250011 | 1715 | Epileptic absences |
| F250100 | 99548 | Pykno-epilepsy |
| F250200 | 24309 | Epileptic seizures - atonic |
| F250300 | 31830 | Epileptic seizures - akinetic |
| F250500 | 34792 | Lennox-Gastaut syndrome |
| F250y00 | 59185 | Other specified generalised nonconvulsive epilepsy |
| F250z00 | 44252 | Generalised nonconvulsive epilepsy NOS |
| F251.00 | 26144 | Generalised convulsive epilepsy |
| F251000 | 988 | Grand mal (major) epilepsy |
| F251011 | 22804 | Tonic-clonic epilepsy |
| F251111 | 49340 | Otohara syndrome |
| F251200 | 18471 | Epileptic seizures - clonic |
| F251300 | 4801 | Epileptic seizures - myoclonic |
| F251400 | 5152 | Epileptic seizures - tonic |
| F251500 | 8187 | Tonic-clonic epilepsy |
| F251y00 | 45927 | Other specified generalised convulsive epilepsy |
| F251z00 | 40806 | Generalised convulsive epilepsy NOS |
| F252.00 | 9886 | Petit mal status |
| F253.00 | 5117 | Grand mal status |
| F253.11 | 4093 | Status epilepticus |
| F254.00 | 32288 | Partial epilepsy with impairment of consciousness |
| F254000 | 3175 | Temporal lobe epilepsy |
| F254100 | 23634 | Psychomotor epilepsy |
| F254200 | 36203 | Psychosensory epilepsy |
| F254300 | 55665 | Limbic system epilepsy |
| F254400 | 34079 | Epileptic automatism |
| F254500 | 11394 | Complex partial epileptic seizure |
| F254z00 | 31920 | Partial epilepsy with impairment of consciousness NOS |
| F255.00 | 26015 | Partial epilepsy without impairment of consciousness |
| F255000 | 9569 | Jacksonian, focal or motor epilepsy |
| F255011 | 5525 | Focal epilepsy |
| F255012 | 65699 | Motor epilepsy |
| F255100 | 48134 | Sensory induced epilepsy |
| F255200 | 37592 | Somatosensory epilepsy |
| F255300 | 73542 | Visceral reflex epilepsy |
| F255311 | 98870 | Partial epilepsy with autonomic symptoms |
| F255400 | 55739 | Visual reflex epilepsy |
| F255500 | 68946 | Unilateral epilepsy |
| F255600 | 40105 | Simple partial epileptic seizure |
| F255y00 | 26733 | Partial epilepsy without impairment of consciousness OS |
| F255z00 | 27526 | Partial epilepsy without impairment of consciousness NOS |
| F257.00 | 71719 | Kojevnikov's epilepsy |
| F25B.00 | 30604 | Alcohol-induced epilepsy |
| F25C.00 | 30816 | Drug-induced epilepsy |
| F25D.00 | 56359 | Menstrual epilepsy |
| F25E.00 | 65673 | Stress-induced epilepsy |
| F25F.00 | 30635 | Photosensitive epilepsy |
| F25X.00 | 6271 | Status epilepticus, unspecified |
| F25y.00 | 38307 | Other forms of epilepsy |
| F25y000 | 55260 | Cursive (running) epilepsy |
| F25y100 | 53483 | Gelastic epilepsy |
| F25y200 | 9887 | Locl-rlt(foc)(part)idiop epilep&epilptic syn seiz locl onset |
| F25y300 | 25330 | Complex partial status epilepticus |
| F25y400 | 19170 | Benign Rolandic epilepsy |
| F25yz00 | 9979 | Other forms of epilepsy NOS |
| F25z.00 | 9747 | Epilepsy NOS |
| F25z.11 | 3607 | Fit (in known epileptic) NOS |
| SC20000 | 4109 | Traumatic epilepsy |
| 6672 | 26511 | Follow-up epilepsy assessment |
| 6674 | 50012 | Epilepsy associated problems |
| 6675 | 8262 | Fit frequency |
| 6676 | 7807 | Last fit |
| 6678 | 26512 | Epilepsy treatment changed |
| 6679 | 34473 | Epilepsy treatment started |

Diabetes

| readcode | medcode | readterm |
| --- | --- | --- |
| 66A3.00 | 7563 | Diabetic on diet only |
| 66A4.00 | 1684 | Diabetic on oral treatment |
| 66A5.00 | 8842 | Diabetic on insulin |
| 66AI.00 | 13071 | Diabetic - good control |
| 66AJ.00 | 2378 | Diabetic - poor control |
| 66AJ.11 | 9013 | Unstable diabetes |
| 66AJ100 | 2478 | Brittle diabetes |
| 66AJz00 | 22023 | Diabetic - poor control NOS |
| 66AK.00 | 43951 | Diabetic - cooperative patient |
| 66AL.00 | 17869 | Diabetic-uncooperative patient |
| 66AV.00 | 28769 | Diabetic on insulin and oral treatment |
| C10..00 | 711 | Diabetes mellitus |
| C100.00 | 38986 | Diabetes mellitus with no mention of complication |
| C100000 | 24490 | Diabetes mellitus, juvenile type, no mention of complication |
| C100011 | 1038 | Insulin dependent diabetes mellitus |
| C100100 | 14803 | Diabetes mellitus, adult onset, no mention of complication |
| C100111 | 14889 | Maturity onset diabetes |
| C100112 | 506 | Non-insulin dependent diabetes mellitus |
| C100z00 | 50972 | Diabetes mellitus NOS with no mention of complication |
| C101.00 | 1682 | Diabetes mellitus with ketoacidosis |
| C101000 | 53200 | Diabetes mellitus, juvenile type, with ketoacidosis |
| C101100 | 54856 | Diabetes mellitus, adult onset, with ketoacidosis |
| C101y00 | 38617 | Other specified diabetes mellitus with ketoacidosis |
| C101z00 | 42505 | Diabetes mellitus NOS with ketoacidosis |
| C102.00 | 21482 | Diabetes mellitus with hyperosmolar coma |
| C102000 | 40023 | Diabetes mellitus, juvenile type, with hyperosmolar coma |
| C102100 | 43139 | Diabetes mellitus, adult onset, with hyperosmolar coma |
| C102z00 | 72345 | Diabetes mellitus NOS with hyperosmolar coma |
| C103.00 | 15690 | Diabetes mellitus with ketoacidotic coma |
| C103000 | 42567 | Diabetes mellitus, juvenile type, with ketoacidotic coma |
| C103100 | 68843 | Diabetes mellitus, adult onset, with ketoacidotic coma |
| C103y00 | 59288 | Other specified diabetes mellitus with coma |
| C103z00 | 65062 | Diabetes mellitus NOS with ketoacidotic coma |
| C104.00 | 16502 | Diabetes mellitus with renal manifestation |
| C104000 | 93922 | Diabetes mellitus, juvenile type, with renal manifestation |
| C104100 | 35105 | Diabetes mellitus, adult onset, with renal manifestation |
| C104y00 | 13279 | Other specified diabetes mellitus with renal complications |
| C104z00 | 35107 | Diabetes mellitus with nephropathy NOS |
| C105.00 | 33254 | Diabetes mellitus with ophthalmic manifestation |
| C105000 | 69748 | Diabetes mellitus, juvenile type, + ophthalmic manifestation |
| C105100 | 41389 | Diabetes mellitus, adult onset, + ophthalmic manifestation |
| C105y00 | 47377 | Other specified diabetes mellitus with ophthalmic complicatn |
| C105z00 | 34283 | Diabetes mellitus NOS with ophthalmic manifestation |
| C106.00 | 16230 | Diabetes mellitus with neurological manifestation |
| C106.12 | 7795 | Diabetes mellitus with neuropathy |
| C106.13 | 16491 | Diabetes mellitus with polyneuropathy |
| C106000 | 67853 | Diabetes mellitus, juvenile, + neurological manifestation |
| C106100 | 39317 | Diabetes mellitus, adult onset, + neurological manifestation |
| C106y00 | 61523 | Other specified diabetes mellitus with neurological comps |
| C106z00 | 22573 | Diabetes mellitus NOS with neurological manifestation |
| C107.00 | 35399 | Diabetes mellitus with peripheral circulatory disorder |
| C107.11 | 32403 | Diabetes mellitus with gangrene |
| C107.12 | 32556 | Diabetes with gangrene |
| C107000 | 70448 | Diabetes mellitus, juvenile +peripheral circulatory disorder |
| C107100 | 63357 | Diabetes mellitus, adult, + peripheral circulatory disorder |
| C107200 | 33807 | Diabetes mellitus, adult with gangrene |
| C107300 | 69124 | IDDM with peripheral circulatory disorder |
| C107400 | 56803 | NIDDM with peripheral circulatory disorder |
| C107z00 | 65025 | Diabetes mellitus NOS with peripheral circulatory disorder |
| C108.00 | 1647 | Insulin dependent diabetes mellitus |
| C108.11 | 18505 | IDDM-Insulin dependent diabetes mellitus |
| C108.12 | 17858 | Type 1 diabetes mellitus |
| C108.13 | 24423 | Type I diabetes mellitus |
| C108000 | 46963 | Insulin-dependent diabetes mellitus with renal complications |
| C108011 | 61344 | Type I diabetes mellitus with renal complications |
| C108012 | 21983 | Type 1 diabetes mellitus with renal complications |
| C108100 | 49276 | Insulin-dependent diabetes mellitus with ophthalmic comps |
| C108200 | 52283 | Insulin-dependent diabetes mellitus with neurological comps |
| C108211 | 49146 | Type I diabetes mellitus with neurological complications |
| C108212 | 61829 | Type 1 diabetes mellitus with neurological complications |
| C108300 | 52104 | Insulin dependent diabetes mellitus with multiple complicatn |
| C108400 | 26855 | Unstable insulin dependent diabetes mellitus |
| C108411 | 60107 | Unstable type I diabetes mellitus |
| C108500 | 44443 | Insulin dependent diabetes mellitus with ulcer |
| C108511 | 51957 | Type I diabetes mellitus with ulcer |
| C108600 | 60499 | Insulin dependent diabetes mellitus with gangrene |
| C108700 | 6509 | Insulin dependent diabetes mellitus with retinopathy |
| C108711 | 38161 | Type I diabetes mellitus with retinopathy |
| C108712 | 41049 | Type 1 diabetes mellitus with retinopathy |
| C108800 | 6791 | Insulin dependent diabetes mellitus - poor control |
| C108811 | 46850 | Type I diabetes mellitus - poor control |
| C108812 | 45914 | Type 1 diabetes mellitus - poor control |
| C108900 | 31310 | Insulin dependent diabetes maturity onset |
| C108911 | 63017 | Type I diabetes mellitus maturity onset |
| C108A00 | 56448 | Insulin-dependent diabetes without complication |
| C108B00 | 24694 | Insulin dependent diabetes mellitus with mononeuropathy |
| C108B11 | 99231 | Type I diabetes mellitus with mononeuropathy |
| C108C00 | 41716 | Insulin dependent diabetes mellitus with polyneuropathy |
| C108D00 | 57621 | Insulin dependent diabetes mellitus with nephropathy |
| C108D11 | 66872 | Type I diabetes mellitus with nephropathy |
| C108E00 | 44440 | Insulin dependent diabetes mellitus with hypoglycaemic coma |
| C108E11 | 42729 | Type I diabetes mellitus with hypoglycaemic coma |
| C108E12 | 70766 | Type 1 diabetes mellitus with hypoglycaemic coma |
| C108F00 | 44260 | Insulin dependent diabetes mellitus with diabetic cataract |
| C108F11 | 17545 | Type I diabetes mellitus with diabetic cataract |
| C108G00 | 64446 | Insulin dependent diab mell with peripheral angiopathy |
| C108H00 | 65616 | Insulin dependent diabetes mellitus with arthropathy |
| C108H11 | 62352 | Type I diabetes mellitus with arthropathy |
| C108J00 | 39809 | Insulin dependent diab mell with neuropathic arthropathy |
| C108J12 | 18230 | Type 1 diabetes mellitus with neuropathic arthropathy |
| C108y00 | 46290 | Other specified diabetes mellitus with multiple comps |
| C108z00 | 64449 | Unspecified diabetes mellitus with multiple complications |
| C109.00 | 4513 | Non-insulin dependent diabetes mellitus |
| C109.11 | 5884 | NIDDM - Non-insulin dependent diabetes mellitus |
| C109.12 | 17859 | Type 2 diabetes mellitus |
| C109.13 | 18219 | Type II diabetes mellitus |
| C109000 | 52303 | Non-insulin-dependent diabetes mellitus with renal comps |
| C109011 | 50225 | Type II diabetes mellitus with renal complications |
| C109012 | 18209 | Type 2 diabetes mellitus with renal complications |
| C109100 | 50429 | Non-insulin-dependent diabetes mellitus with ophthalm comps |
| C109111 | 59725 | Type II diabetes mellitus with ophthalmic complications |
| C109112 | 70316 | Type 2 diabetes mellitus with ophthalmic complications |
| C109200 | 55842 | Non-insulin-dependent diabetes mellitus with neuro comps |
| C109211 | 67905 | Type II diabetes mellitus with neurological complications |
| C109212 | 45919 | Type 2 diabetes mellitus with neurological complications |
| C109300 | 62146 | Non-insulin-dependent diabetes mellitus with multiple comps |
| C109400 | 34912 | Non-insulin dependent diabetes mellitus with ulcer |
| C109411 | 55075 | Type II diabetes mellitus with ulcer |
| C109412 | 65704 | Type 2 diabetes mellitus with ulcer |
| C109500 | 40401 | Non-insulin dependent diabetes mellitus with gangrene |
| C109511 | 62107 | Type II diabetes mellitus with gangrene |
| C109600 | 17262 | Non-insulin-dependent diabetes mellitus with retinopathy |
| C109611 | 58604 | Type II diabetes mellitus with retinopathy |
| C109612 | 42762 | Type 2 diabetes mellitus with retinopathy |
| C109700 | 8403 | Non-insulin dependent diabetes mellitus - poor control |
| C109711 | 24458 | Type II diabetes mellitus - poor control |
| C109712 | 45913 | Type 2 diabetes mellitus - poor control |
| C109900 | 29979 | Non-insulin-dependent diabetes mellitus without complication |
| C109A00 | 72320 | Non-insulin dependent diabetes mellitus with mononeuropathy |
| C109A11 | 50813 | Type II diabetes mellitus with mononeuropathy |
| C109B00 | 45467 | Non-insulin dependent diabetes mellitus with polyneuropathy |
| C109B11 | 47409 | Type II diabetes mellitus with polyneuropathy |
| C109C00 | 59365 | Non-insulin dependent diabetes mellitus with nephropathy |
| C109C11 | 64571 | Type II diabetes mellitus with nephropathy |
| C109C12 | 24836 | Type 2 diabetes mellitus with nephropathy |
| C109D00 | 43785 | Non-insulin dependent diabetes mellitus with hypoglyca coma |
| C109D11 | 56268 | Type II diabetes mellitus with hypoglycaemic coma |
| C109D12 | 61071 | Type 2 diabetes mellitus with hypoglycaemic coma |
| C109E00 | 69278 | Non-insulin depend diabetes mellitus with diabetic cataract |
| C109E11 | 48192 | Type II diabetes mellitus with diabetic cataract |
| C109E12 | 44779 | Type 2 diabetes mellitus with diabetic cataract |
| C109F00 | 54212 | Non-insulin-dependent d m with peripheral angiopath |
| C109F11 | 54899 | Type II diabetes mellitus with peripheral angiopathy |
| C109F12 | 60699 | Type 2 diabetes mellitus with peripheral angiopathy |
| C109G00 | 24693 | Non-insulin dependent diabetes mellitus with arthropathy |
| C109G11 | 18143 | Type II diabetes mellitus with arthropathy |
| C109G12 | 49869 | Type 2 diabetes mellitus with arthropathy |
| C109H00 | 40962 | Non-insulin dependent d m with neuropathic arthropathy |
| C109H11 | 47816 | Type II diabetes mellitus with neuropathic arthropathy |
| C109H12 | 66965 | Type 2 diabetes mellitus with neuropathic arthropathy |
| C109J00 | 18278 | Insulin treated Type 2 diabetes mellitus |
| C109J11 | 37648 | Insulin treated non-insulin dependent diabetes mellitus |
| C109J12 | 18264 | Insulin treated Type II diabetes mellitus |
| C109K00 | 36633 | Hyperosmolar non-ketotic state in type 2 diabetes mellitus |
| C10C.00 | 43453 | Diabetes mellitus autosomal dominant |
| C10C.11 | 46624 | Maturity onset diabetes in youth |
| C10D.00 | 36695 | Diabetes mellitus autosomal dominant type 2 |
| C10D.11 | 59991 | Maturity onset diabetes in youth type 2 |
| C10E.00 | 1549 | Type 1 diabetes mellitus |
| C10E.11 | 12455 | Type I diabetes mellitus |
| C10E.12 | 51261 | Insulin dependent diabetes mellitus |
| C10E000 | 47582 | Type 1 diabetes mellitus with renal complications |
| C10E100 | 47649 | Type 1 diabetes mellitus with ophthalmic complications |
| C10E200 | 42831 | Type 1 diabetes mellitus with neurological complications |
| C10E300 | 47650 | Type 1 diabetes mellitus with multiple complications |
| C10E312 | 45276 | Insulin dependent diabetes mellitus with multiple complicat |
| C10E400 | 43921 | Unstable type 1 diabetes mellitus |
| C10E411 | 49949 | Unstable type I diabetes mellitus |
| C10E412 | 54600 | Unstable insulin dependent diabetes mellitus |
| C10E500 | 18683 | Type 1 diabetes mellitus with ulcer |
| C10E600 | 69993 | Type 1 diabetes mellitus with gangrene |
| C10E700 | 18387 | Type 1 diabetes mellitus with retinopathy |
| C10E800 | 35288 | Type 1 diabetes mellitus - poor control |
| C10E812 | 72702 | Insulin dependent diabetes mellitus - poor control |
| C10E900 | 40682 | Type 1 diabetes mellitus maturity onset |
| C10EA00 | 69676 | Type 1 diabetes mellitus without complication |
| C10EA11 | 62613 | Type I diabetes mellitus without complication |
| C10EB00 | 68105 | Type 1 diabetes mellitus with mononeuropathy |
| C10EC00 | 46301 | Type 1 diabetes mellitus with polyneuropathy |
| C10ED00 | 10418 | Type 1 diabetes mellitus with nephropathy |
| C10EE00 | 39070 | Type 1 diabetes mellitus with hypoglycaemic coma |
| C10EF00 | 49554 | Type 1 diabetes mellitus with diabetic cataract |
| C10EG00 | 93468 | Type 1 diabetes mellitus with peripheral angiopathy |
| C10EH00 | 18642 | Type 1 diabetes mellitus with arthropathy |
| C10EJ00 | 54008 | Type 1 diabetes mellitus with neuropathic arthropathy |
| C10EK00 | 30323 | Type 1 diabetes mellitus with persistent proteinuria |
| C10EL00 | 30294 | Type 1 diabetes mellitus with persistent microalbuminuria |
| C10EM00 | 10692 | Type 1 diabetes mellitus with ketoacidosis |
| C10EM11 | 62209 | Type I diabetes mellitus with ketoacidosis |
| C10EN00 | 40837 | Type 1 diabetes mellitus with ketoacidotic coma |
| C10EN11 | 66145 | Type I diabetes mellitus with ketoacidotic coma |
| C10EP00 | 22871 | Type 1 diabetes mellitus with exudative maculopathy |
| C10EQ00 | 55239 | Type 1 diabetes mellitus with gastroparesis |
| C10F.00 | 758 | Type 2 diabetes mellitus |
| C10F.11 | 22884 | Type II diabetes mellitus |
| C10F000 | 18777 | Type 2 diabetes mellitus with renal complications |
| C10F011 | 57278 | Type II diabetes mellitus with renal complications |
| C10F100 | 47321 | Type 2 diabetes mellitus with ophthalmic complications |
| C10F200 | 34268 | Type 2 diabetes mellitus with neurological complications |
| C10F300 | 65267 | Type 2 diabetes mellitus with multiple complications |
| C10F311 | 43227 | Type II diabetes mellitus with multiple complications |
| C10F400 | 49074 | Type 2 diabetes mellitus with ulcer |
| C10F500 | 12736 | Type 2 diabetes mellitus with gangrene |
| C10F511 | 104323 | Type II diabetes mellitus with gangrene |
| C10F600 | 18496 | Type 2 diabetes mellitus with retinopathy |
| C10F611 | 49655 | Type II diabetes mellitus with retinopathy |
| C10F700 | 25627 | Type 2 diabetes mellitus - poor control |
| C10F711 | 47315 | Type II diabetes mellitus - poor control |
| C10F900 | 47954 | Type 2 diabetes mellitus without complication |
| C10F911 | 53392 | Type II diabetes mellitus without complication |
| C10FA00 | 62674 | Type 2 diabetes mellitus with mononeuropathy |
| C10FB00 | 18425 | Type 2 diabetes mellitus with polyneuropathy |
| C10FB11 | 50527 | Type II diabetes mellitus with polyneuropathy |
| C10FC00 | 12640 | Type 2 diabetes mellitus with nephropathy |
| C10FC11 | 102201 | Type II diabetes mellitus with nephropathy |
| C10FD00 | 46917 | Type 2 diabetes mellitus with hypoglycaemic coma |
| C10FE00 | 44982 | Type 2 diabetes mellitus with diabetic cataract |
| C10FF00 | 37806 | Type 2 diabetes mellitus with peripheral angiopathy |
| C10FG00 | 59253 | Type 2 diabetes mellitus with arthropathy |
| C10FH00 | 35385 | Type 2 diabetes mellitus with neuropathic arthropathy |
| C10FJ00 | 1407 | Insulin treated Type 2 diabetes mellitus |
| C10FJ11 | 64668 | Insulin treated Type II diabetes mellitus |
| C10FK00 | 34450 | Hyperosmolar non-ketotic state in type 2 diabetes mellitus |
| C10FL00 | 26054 | Type 2 diabetes mellitus with persistent proteinuria |
| C10FL11 | 60796 | Type II diabetes mellitus with persistent proteinuria |
| C10FM00 | 18390 | Type 2 diabetes mellitus with persistent microalbuminuria |
| C10FN00 | 32627 | Type 2 diabetes mellitus with ketoacidosis |
| C10FP00 | 51756 | Type 2 diabetes mellitus with ketoacidotic coma |
| C10FQ00 | 25591 | Type 2 diabetes mellitus with exudative maculopathy |
| C10FR00 | 63690 | Type 2 diabetes mellitus with gastroparesis |
| C10G.00 | 51697 | Secondary pancreatic diabetes mellitus |
| C10G000 | 96506 | Secondary pancreatic diabetes mellitus without complication |
| C10y.00 | 33343 | Diabetes mellitus with other specified manifestation |
| C10y100 | 63371 | Diabetes mellitus, adult, + other specified manifestation |
| C10yy00 | 10098 | Other specified diabetes mellitus with other spec comps |
| C10yz00 | 70821 | Diabetes mellitus NOS with other specified manifestation |
| C10z.00 | 45491 | Diabetes mellitus with unspecified complication |
| C10z000 | 68792 | Diabetes mellitus, juvenile type, + unspecified complication |
| C10z100 | 63762 | Diabetes mellitus, adult onset, + unspecified complication |
| C10zy00 | 64283 | Other specified diabetes mellitus with unspecified comps |
| C10zz00 | 64357 | Diabetes mellitus NOS with unspecified complication |
| Cyu2.00 | 52212 | [X]Diabetes mellitus |
| Cyu2000 | 41686 | [X]Other specified diabetes mellitus |
| Cyu2300 | 100292 | [X]Unspecified diabetes mellitus with renal complications |
| L180500 | 50960 | Pre-existing diabetes mellitus, insulin-dependent |
| L180600 | 50609 | Pre-existing diabetes mellitus, non-insulin-dependent |
| L180X00 | 55431 | Pre-existing diabetes mellitus, unspecified |

Chemotherapy or radiotherapy

| medcode | readcode | readterm |
| --- | --- | --- |
| 783 | 8BAD.00 | Chemotherapy |
| 320 | 7M37100 | Radiotherapy NEC |
| 14887 | 8BA5.00 | Oral chemotherapy |
| 5019 | 8BAD000 | Cancer chemotherapy |
| 30942 | 9N1yC00 | Seen in radiotherapy clinic |
| 18079 | 8HB7.00 | Chemotherapy follow-up |
| 9706 | 5154 | Radiotherapy completed |
| 21318 | 8BA5.11 | Oral cytotoxic drug therapy |
| 8729 | 8J0..00 | Brachytherapy |
| 10932 | 8H67.00 | Referred for radiotherapy |
| 5527 | ZV58000 | [V]Radiotherapy session |
| 17048 | 863..12 | Radiation therapy |
| 49464 | 863..00 | Phototherapy/radiation therapy |
| 38466 | 5151 | Radiotherapy started |
| 21576 | TJ31D00 | Adverse reaction to methotrexate |
| 51781 | ZVu3L00 | [X]Other chemotherapy |
| 31489 | ZV58800 | [V]Chemotherapy session for neoplasm |
| 20443 | ZV67200 | [V]Chemotherapy follow-up |
| 36981 | 5155 | Awaiting radiotherapy |
| 16935 | 515..00 | Progress of radiotherapy |
| 29285 | 5149 | Radiotherapy-tumour palliation |
| 40490 | 7046200 | Intrathecal chemotherapy |
| 10542 | 5A16.00 | Radioactive drug therapy |
| 59684 | 7M0c.00 | Radiotherapy procedures |
| 28712 | 8J01.00 | Iodine seed radiotherapy |
| 18715 | 5AB..00 | Stereotactic radiotherapy |
| 20381 | ZV58100 | [V]Maintenance chemotherapy |
| 31804 | TB12100 | Radiotherapy procedure with complication, without blame |
| 10776 | 5A...11 | Radiotherapy - internal |
| 46824 | 8H2G.00 | Admit radiotherapy emergency |
| 22472 | 59Z..00 | External radiotherapy NOS |
| 38662 | 7M0P000 | Introduction of radioactive caesium into organ NOC |
| 29679 | 5146 | Radiotherapy - post-op.control |
| 40424 | 8HB5.00 | Combined therapy follow-up |
| 29301 | 514Z.00 | Radiotherapy purpose - NOS |
| 15362 | 7E0C000 | Introduction of radioactive substance into uterine cavity |
| 5404 | 7M0P.00 | Introduction removable radioactive material into organ NOC |
| 41044 | 8J...00 | Radiotherapy treatment groups |
| 97412 | 7M0P400 | Intro radioactive substance into organ for brachytherapy NOC |
| 25479 | ZV67800 | [V]Follow-up examin after chemotherapy for malign neoplasm |
| 48690 | 863Z.00 | Radiation/phototherapy NOS |
| 16662 | ZV66100 | [V]Convalescence after radiotherapy |
| 23589 | 7M0Pz00 | Introduction removable radioactive material to organ NOC NOS |
| 92316 | TJ31C00 | Adverse reaction to mercaptopurine |
| 39951 | 5A9..00 | Selectron therapy |
| 40070 | 7101100 | Implantation of radioactive substance into pituitary gland |
| 91102 | 7M0cz00 | Radiotherapy procedures NOS |
| 94247 | TJ31z00 | Adverse reaction to antineoplastic/immunosuppress drugs NOS |
| 35597 | 5147 | Radiotherapy for analgesia |
| 45954 | TJ31.00 | Adverse reaction to antineoplastic & immunosuppressive drugs |
| 64100 | TJ07300 | Adverse reaction to mitomycin |
| 21241 | TJ31500 | Adverse reaction to lomustine |
| 60176 | TJ31J00 | Adverse reaction to carboplatin |
| 36810 | 5AC..00 | Strontium 89 therapy |
| 44831 | 5153 | Radiotherapy stopped |
| 62202 | 8J00.00 | High dose brachytherapy |
| 43261 | 5144 | Radiotherapy - pre-op. control |
| 44148 | 5914.11 | Deep X-ray therapy |
| 20336 | 7272200 | Radiotherapy to lesion of retina |
| 87860 | 7M0P300 | Intro radioactive substance org interstit brachytherapy NOC |
| 52368 | TJ07100 | Adverse reaction to bleomycin |
| 45099 | 515Z.00 | Radiotherapy progress NOS |
| 54919 | 5A8Z.00 | Other radiotherapy NOS |
| 60674 | 7052200 | Radiotherapy to lesion of peripheral nerve |
| 49240 | TJ31300 | Adverse reaction to cyclophosphamide |
| 54828 | 7D15400 | Implantation of radioactive substance into vagina |
| 53180 | 5A46.00 | Radioth.: temp. pelvic implant |
| 36225 | 5A4..11 | Radium needles |
| 20282 | 7M0Py00 | Introduction removable radioactive material to organ NOC OS |
| 60467 | TJ31200 | Adverse reaction to chlorambucil |
| 71837 | U603300 | [X]Other antineoplast drugs caus adverse eff in therap use |
| 38773 | 5A...00 | Other nuclear therapy |
| 53477 | ZV67811 | [V]Follow-up examination after chemotherapy for leukaemia |
| 42351 | 591..00 | X-ray beam therapy |
| 52059 | TJ31K00 | Adverse reaction to cisplatin |
| 56407 | TJ31L00 | Adverse reaction to hydroxyurea |
| 51563 | TJ31B00 | Adverse reaction to fluorouracil |
| 43755 | TJ31H00 | Adverse reaction to vincristine sulphate |
| 51787 | 5975 | Ext.beam + chemotherapy |
| 61955 | 5AA..00 | Iridium wire therapy |
| 70386 | TA32.00 | Overdose of radiation in therapy |
| 55832 | 5143 | Radioth. for lymphat.irradiat. |
| 98625 | U603100 | [X]Antineoplast antimetabs caus adverse eff in therap use |
| 57591 | 591Z.00 | X-ray beam therapy NOS |
| 58036 | 7220100 | Radiotherapy to lacrimal gland |
| 73173 | 5A13.00 | Bone marrow suppres.-irradiat. |
| 69979 | 5AZ..00 | Other nuclear therapy NOS |
| 100390 | 7M0P500 | Intro non-remov radioact subst into organ for brachyther NOC |
| 93937 | TJ31A00 | Adverse reaction to cytarabine |
| 59796 | 5914 | Deep X-ray therapy 150-400 Kv |
| 67248 | 5A12.00 | Thyroid tumour/metast irradiat |
| 62951 | 5A15.00 | Bone tumour/metast.irradiat. |
| 97793 | TJ31F00 | Adverse reaction to etoposide |
| 96446 | U613200 | [X]Overdose of radiation given during therapy |
| 85984 | 5941 | Proton therapy |
| 73172 | 5A7Z.00 | Radio-chemotherapy NOS |
| 70478 | 5A4..00 | Radioth.: temporary implant |
| 73692 | 5A4Z.00 | Radioth.:temporary implant NOS |
| 50731 | 7H2C000 | Introduction of radioactive substance into peritoneal cavity |
| 93599 | SL07300 | Daunorubicin poisoning |
| 64440 | TJ31600 | Adverse reaction to melphalan |
| 60399 | TJ31400 | Adverse reaction to estramustine phosphate |
| 108751 | TJ31S11 | Adverse reaction to ciclosporin |
| 55828 | 5145 | Radiotherapy -intra-op.control |
| 94306 | 593..00 | Fast-electron therapy |
| 95066 | 5961 | Radium contact therapy |
| 94305 | 596..00 | Short dis.+contact radiotherap |
| 64143 | 597Z.00 | Combined radiotherapy NOS |
| 72978 | 597..00 | Combined radiotherapy |
| 71598 | 5A45.00 | Radioth.: temp. abdom. implant |
| 106334 | 5A22.00 | Radioth.: infuse - head/neck |
| 70246 | 5A1..00 | Internal metabolic radiotherap |
| 65739 | 5A53.00 | Preload radioth.- nose |
| 103372 | 5A2..00 | Intern.radioth-unsealed source |
| 64801 | 5A3..00 | Intern.radioth-permanent seeds |
| 98826 | 5A58.00 | Preload radioth-female genital |
| 71599 | 5A1Z.00 | Internal metabolic radioth.NOS |
| 104811 | TJ07000 | Adverse reaction to actinomycin D |
| 68859 | TJ07200 | Adverse reaction to doxorubicin hydrochloride |
| 113181 | TJ07.00 | Adverse reaction to antineoplastic antibiotics |
| 112264 | TJ31100 | Adverse reaction to carmustine |

Cough

| medcode | readcode | readterm |
| --- | --- | --- |
| 92 | 171..00 | Cough |
| 1273 | 171..11 | C/O - cough |
| 3068 | 1717 | Night cough present |
| 292 | 1719 | Chesty cough |
| 1160 | R062.00 | [D]Cough |
| 4931 | 1712 | Dry cough |
| 7773 | 1714 | Productive cough -green sputum |
| 1234 | 1716 | Productive cough NOS |
| 3628 | 171B.00 | Persistent cough |
| 7706 | 1713 | Productive cough -clear sputum |
| 7708 | 1715 | Productive cough-yellow sputum |
| 9807 | 171..12 | Sputum - symptom |
| 7707 | 171Z.00 | Cough symptom NOS |
| 1612 | 171A.00 | Chronic cough |
| 3645 | 1716.11 | Coughing up phlegm |
| 1025 | 1719.11 | Bronchial cough |
| 4070 | 171C.00 | Morning cough |
| 6475 | R06..00 | [D]Respiratory system and chest symptoms |
| 1251 | R064.00 | [D]Abnormal sputum |
| 19470 | 171J.00 | Reflux cough |
| 15430 | R064100 | [D]Sputum abnormal - colour |
| 18907 | 171F.00 | Cough with fever |
| 9799 | R062000 | [D]Cough syncope |
| 29318 | 171D.00 | Evening cough |
| 22318 | 171H.00 | Difficulty in coughing up sputum |
| 104163 | R062100 | [D]Episodic dry cough |
| 90332 | 171K.00 | Barking cough |
| 100333 | 171L.00 | Cough on exercise |
| 16468 | R06zz00 | [D]Respiratory system and chest symptoms NOS |
| 43795 | 171E.00 | Unexplained cough |
| 47638 | R06z.00 | [D]Other respiratory system and chest symptoms |
| 23582 | R064z00 | [D]Abnormal sputum NOS |
| 26211 | 171G.00 | Bovine cough |
| 44214 | R064200 | [D]Sputum abnormal - odour |

Dyspnoea

| medcode | readcode | readterm |
| --- | --- | --- |
| 4822 | 1739 | Shortness of breath |
| 741 | R060800 | [D]Shortness of breath |
| 19427 | 173I.00 | MRC Breathlessness Scale: grade 2 |
| 1429 | 173..00 | Breathlessness |
| 19426 | 173J.00 | MRC Breathlessness Scale: grade 3 |
| 19432 | 173H.00 | MRC Breathlessness Scale: grade 1 |
| 5349 | 173..13 | Shortness of breath symptom |
| 5175 | 173..11 | Breathlessness symptom |
| 19430 | 173K.00 | MRC Breathlessness Scale: grade 4 |
| 3092 | R060A00 | [D]Dyspnoea |
| 2575 | 173C.00 | Short of breath on exertion |
| 5896 | 173..12 | Dyspnoea - symptom |
| 6326 | 1732 | Breathless - moderate exertion |
| 2931 | 1738 | Difficulty breathing |
| 7932 | 1733 | Breathless - mild exertion |
| 12474 | 173C.12 | SOBOE |
| 19429 | 173L.00 | MRC Breathlessness Scale: grade 5 |
| 57903 | 388H.00 | CLASP shortness of breath score |
| 735 | R060D00 | [D]Breathlessness |
| 2737 | Q30..00 | Respiratory distress syndrome |
| 6434 | 1736 | Paroxysmal nocturnal dyspnoea |
| 7000 | 2322 | O/E - dyspnoea |
| 31143 | 1734 | Breathless - at rest |
| 11451 | R060200 | [D]Orthopnoea |
| 7683 | 1735 | Breathless - lying flat |
| 21801 | 173Z.00 | Breathlessness NOS |
| 2563 | R060600 | [D]Respiratory distress |
| 18116 | 173D.00 | Nocturnal dyspnoea |
| 24889 | 173G.00 | Breathless - strenuous exertion |
| 7534 | 2324 | O/E - respiratory distress |
| 9089 | 1735.11 | Orthopnoea symptom |
| 37704 | 2323 | O/E - orthopnoea |
| 53771 | 173C.11 | Dyspnoea on exertion |
| 24848 | H585300 | Adult respiratory distress syndrome |
| 22094 | 173F.00 | Short of breath dressing/undressing |
| 9297 | R060700 | [D]Respiratory insufficiency |
| 57193 | 173R.00 | Borg Breathlessness Score: 3 moderate |
| 57759 | 173Q.00 | Borg Breathlessness Score: 2 slight |
| 59860 | 173S.00 | Borg Breathlessness Score: 4 somewhat severe |
| 68707 | 173P.00 | Borg Breathlessness Score: 1 very slight |
| 64049 | 173T.00 | Borg Breathlessness Score: 5 severe |
| 70818 | 173N.00 | Borg Breathlessness Score: 0.5 very, very slight |
| 60096 | ZR3Q.00 | CLASP shortness of breath score |
| 57678 | H585.11 | Adult respiratory distress syndrome |
| 70061 | 173W.00 | Borg Breathlessness Score: 7 very severe |
| 72334 | 173X.00 | Borg Breathlessness Score: 8 very severe (+) |
| 42287 | 173V.00 | Borg Breathlessness Score: 6 severe (+) |
| 101843 | 173a.00 | Borg Breathlessness Score: 10 maximal |
| 67566 | 173Y.00 | Borg Breathlessness Score: 9 very, very sev (almost maximal) |
| 36347 | R060000 | [D]Respiratory symptom, unspecified |
| 39958 | R060.00 | [D]Respiratory abnormalities |
| 101421 | R060H00 | [D]Very severe wheeze |
| 101073 | R060G00 | [D]Severe wheeze |
| 2395 | R060z00 | [D]Respiratory abnormalities NOS |
| 820 | R060300 | [D]Tachypnoea |
| 100954 | R060E00 | [D]Mild wheeze |
| 3092 | R060A00 | [D]Dyspnoea |
| 1270 | R065800 | [D]Chest tightness |
| 2210 | R060900 | [D]Wheezing |
| 982 | R060400 | [D]Apnoea |

Chest infection

| medcode | readcode | readterm |
| --- | --- | --- |
| 68 | H06z011 | Chest infection |
| 148 | H30..00 | Bronchitis unspecified |
| 152 | H302.00 | Wheezy bronchitis |
| 293 | H06z111 | Respiratory tract infection |
| 312 | H060.00 | Acute bronchitis |
| 556 | NA | influenza |
| 978 | H51..00 | Pleurisy |
| 1019 | H061.00 | Acute bronchiolitis |
| 1382 | NA | acute viral bronchitis unspecified |
| 1934 | H301.00 | Laryngotracheobronchitis |
| 2157 | NA | flu like illness |
| 2375 | H50..00 | Empyema |
| 2476 | NA | chest cold |
| 2581 | H06z000 | Chest infection NOS |
| 3163 | H300.00 | Tracheobronchitis NOS |
| 3358 | H06z100 | Lower resp tract infection |
| 3480 | H30z.00 | Bronchitis NOS |
| 5947 | NA | influenza like illness |
| 5978 | H060.11 | Acute wheezy bronchitis |
| 6124 | H062.00 | Acute lower respiratory tract infection |
| 6181 | NA | obliterating fibrous bronchiolitis |
| 8980 | NA | influenza-like symptoms |
| 9043 | H060600 | Acute pneumococcal bronchitis |
| 11072 | H060300 | Acute purulent bronchitis |
| 11101 | H060500 | Acute tracheobronchitis |
| 14791 | NA | influenza with gastrointestinal tract involvement |
| 15774 | NA | influenza with laryngitis |
| 16388 | NA | influenza nos |
| 17185 | H061200 | Acute bronchiolitis with bronchospasm |
| 17359 | H30..11 | Chest infection - unspecified bronchitis |
| 17917 | H061z00 | Acute bronchiolitis NOS |
| 18451 | NA | acute bronchiolitis due to respiratory syncytial virus |
| 19207 | H510300 | Acute dry pleurisy |
| 20198 | H060z00 | Acute bronchitis NOS |
| 21145 | H060400 | Acute croupous bronchitis |
| 21492 | H060800 | Acute haemophilus influenzae bronchitis |
| 23488 | NA | influenza with respiratory manifestations nos |
| 24316 | H24..11 | Chest infection with infectious disease EC |
| 24800 | H060x00 | Acute bacterial bronchitis unspecified |
| 29273 | NA | acute bronchitis due to parainfluenza virus |
| 29617 | NA | influenza with pharyngitis |
| 29669 | H06..00 | Acute bronchitis and bronchiolitis |
| 31363 | NA | influenza with other manifestations nos |
| 31886 | H060A00 | Acute bronchitis due to mycoplasma pneumoniae |
| 32818 | H510900 | Pneumococcal pleurisy |
| 34651 | H500100 | Empyema with bronchopleural fistula |
| 37447 | H06z112 | Acute lower respiratory tract infection |
| 41137 | H06z.00 | Acute bronchitis or bronchiolitis NOS |
| 41589 | NA | acute obliterating bronchiolitis |
| 43362 | H060700 | Acute streptococcal bronchitis |
| 43625 | NA | influenza with other respiratory manifestation |
| 44425 | H501200 | Pleural empyema |
| 46157 | NA | influenza with encephalopathy |
| 47472 | NA | influenza with other manifestations |
| 48593 | NA | acute bronchitis due to respiratory syncytial virus |
| 49452 | H501400 | Purulent pleurisy |
| 49794 | NA | acute neisseria catarrhalis bronchitis |
| 50396 | H060000 | Acute fibrinous bronchitis |
| 54533 | H061000 | Acute capillary bronchiolitis |
| 64890 | NA | acute bronchitis due to rhinovirus |
| 65916 | NA | acute bronchitis due to echovirus |
| 66228 | NA | acute bronchiolitis due to other specified organisms |
| 66397 | NA | [x]other acute lower respiratory infections |
| 69192 | H061300 | acute exudative bronchiolitis |
| 71370 | NA | acute pseudomembranous bronchitis |
| 73100 | NA | [x]acute bronchitis due to other specified organisms |
| 101775 | NA | acute membranous bronchitis |
| 106650 | H583200 | Eosinophilic bronchitis |

| medcode | readcode | readterm |
| --- | --- | --- |
| 374 | 182..00 | Chest pain |
| 3518 | R065A00 | [D]Musculoskeletal chest pain |
| 2519 | 182B.00 | Rib pain |
| 1059 | 1825 | Pleuritic pain |
| 726 | 1828 | Atypical chest pain |
| 2584 | R065.00 | [D]Chest pain |
| 7878 | R065600 | [D]Chest discomfort |
| 14823 | R065200 | [D]Anterior chest wall pain |
| 3796 | R065z00 | [D]Chest pain NOS |
| 9698 | 1824 | Anterior chest wall pain |
| 1228 | 1829 | Retrosternal pain |
| 24704 | 182C.00 | Chest wall pain |
| 10370 | 182Z.00 | Chest pain NOS |
| 7346 | 1822 | Central chest pain |
| 9340 | R065B00 | [D]Non cardiac chest pain |
| 18134 | 182A.00 | Chest pain on exertion |
| 544 | R065000 | [D]Chest pain, unspecified |
| 7844 | R065B14 | [D]Non-cardiac chest pain |
| 8264 | 8HTG.00 | Referred to acute chest pain clinic |
| 8349 | 182B000 | Costal margin chest pain |
| 1865 | 1823 | Precordial pain |
| 18183 | R065400 | [D]Pleuritic pain |
| 24321 | 1D22000 | Chest wall tenderness |
| 1283 | 1827.11 | Pleurodynia |
| 20481 | R065100 | [D]Precordial pain |
| 21082 | R065C00 | [D]Retrosternal chest pain |
| 14819 | R065300 | [D]Painful respiration NOS |
| 15528 | R065700 | [D]Chest pressure |
| 19199 | R065900 | [D]Parasternal chest pain |
| 20490 | 1827 | Painful breathing -pleurodynia |
| 32612 | 1826 | Parasternal pain |
| 50477 | R065D00 | [D]Central chest pain |
| 53806 | Ryu0400 | [X]Other chest pain |
| 24761 | R065500 | [D]Pleurodynia |
| 29490 | R065011 | [D] Retrosternal chest pain |
| 55943 | 182a.00 | Chest pain on exertion |

Fatigue

| medcode | readcode | readterm |
| --- | --- | --- |
| 5794 | 168..00 | Tiredness symptom |
| 5751 | 1683 | Tired all the time |
| 1147 | R007500 | [D]Tiredness |
| 5583 | 168..12 | Lethargy - symptom |
| 15516 | 1683.11 | C/O - 'tired all the time' |
| 1404 | 1682 | Fatigue |
| 5658 | R007000 | [D]Malaise |
| 1371 | R007300 | [D]Lethargy |
| 1816 | 168..13 | Malaise - symptom |
| 2495 | 13CA.00 | Housebound |
| 7235 | E205.12 | Tired all the time |
| 6242 | 168..11 | Fatigue - symptom |
| 6029 | 1B3..12 | Weakness symptoms |
| 1900 | R2y3.00 | [D]Debility, unspecified |
| 3361 | E205.00 | Neurasthenia - nervous debility |
| 1688 | R007100 | [D]Fatigue |
| 2855 | 1B32.00 | Weakness present |
| 5049 | R007200 | [D]Asthenia NOS |
| 17736 | 1684 | Malaise/lethargy |
| 9823 | 1684.11 | C/O - debility - malaise |
| 5814 | R007z11 | [D]Lassitude |
| 5418 | 168..14 | C/O 'Muzzy head' |
| 29292 | 168Z.00 | Tiredness symptom NOS |
| 9220 | 1688 | Exhaustion |
| 1582 | E205.11 | Nervous exhaustion |
| 9889 | R007211 | [D]General weakness |
| 15339 | 13C6.11 | Bedbound |
| 13027 | 1686 | Heavy legs |
| 23932 | R007z00 | [D]Malaise and fatigue NOS |
| 21460 | 1687 | Heavy feeling |
| 44215 | R007.00 | [D]Malaise and fatigue |
| 16561 | Eu46000 | [X]Neurasthenia |
| 16790 | 1684.12 | C/O - overwork |
| 17083 | SN45.00 | Excessive exertion exhaustion |
| 94051 | 13CV.00 | Temporarily housebound |
| 17526 | L39y500 | Maternal exhaustion |
| 24382 | R204.00 | [D]Senile exhaustion |
| 24354 | R007z12 | [D]Overwork |
| 43047 | R202.00 | [D]Senile asthenia |
| 61753 | Eu46y14 | [X]Psychasthenia |
| 90597 | Eu46y15 | [X]Psychasthenia neurosis |

Thrombocytosis

| medcode | readcode | readterm |
| --- | --- | --- |
| 103622 | D3y1.00 | Reactive thrombocytosis |
| 103910 | D3y1.11 | Secondary thrombocytosis |
| 6307 | D3y0.00 | Essential thrombocytosis |

Haemoptysis

| medcode | readcode | readterm |
| --- | --- | --- |
| 4135 | 172..00 | Blood in sputum - haemoptysis |
| 2244 | R063.00 | [D]Haemoptysis |
| 107548 | 1720 | Massive haemoptysis |
| 33742 | R063z00 | [D]Haemoptysis NOS |
| 1610 | 172..12 | Haemoptysis - symptom |
| 10013 | 172..11 | Blood in sputum - symptom |
| 8239 | R063000 | [D]Cough with haemorrhage |
| 7285 | R063100 | [D]Pulmonary haemorrhage NOS |
| 64096 | 4E24.00 | Sputum: contains blood |
| 102351 | 4E2G.00 | Bloodstained sputum |

Weight loss

| medcode | readcode | readterm |
| --- | --- | --- |
| 61420 | ZC2CN00 | Dietary advice for weight gain |
| 26473 | 22A1.00 | O/E - weight > 20% below ideal |
| 104002 | 22A9.00 | Percentage weight loss |
| 29029 | 22A2.00 | O/E -weight 10-20% below ideal |
| 102563 | 1627 | Unintentional weight loss |
| 37937 | 22A8.00 | Weight loss from baseline weight |
| 4663 | 1625 | Abnormal weight loss |
| 12398 | 1D1A.00 | Complaining of weight loss |
| 654 | 1623 | Weight decreasing |
| 3647 | R032.00 | [D]Abnormal loss of weight |
| 5812 | 1625.11 | Abnormal weight loss - symptom |

Finger clubbing

| medcode | readcode | readterm |
| --- | --- | --- |
| 51048 | 2G28.00 | O/E - hands - finger clubbing |
| 7575 | 2G28.11 | O/E - finger clubbing |
| 14752 | R015.00 | [D]Clubbing of fingers |

Lymphadenopathy

| medcode | readcode | readterm |
| --- | --- | --- |
| 51048 | 2G28.00 | O/E - hands - finger clubbing |
| 7575 | 2G28.11 | O/E - finger clubbing |
| 14752 | R015.00 | [D]Clubbing of fingers |

Appetite loss

| medcode | readcode | readterm |
| --- | --- | --- |
| 1855 | R030000 | [D]Appetite loss |
| 6607 | 1612.12 | Loss of appetite - symptom |
| 17203 | Eu50y12 | [X]Psychogenic loss of appetite |
| 22820 | E275600 | Non-organic loss of appetite |
| 13081 | 1615 | Reduced appetite |
| 6099 | 161..00 | Appetite symptom |
| 110527 | 1616 | Observation of appetite |
| 7608 | 1612 | Appetite loss - anorexia |
| 113569 | 39C4A00 | Waterlow appetite score |
| 14717 | 161Z.00 | Appetite symptom NOS |
| 7744 | 1612.11 | Anorexia symptom |
| 53746 | R030z00 | [D]Anorexia NOS |
| 912 | R030.00 | [D]Anorexia |
